# HO-1 impairs the efficacy of radiotherapy by redistributing cGAS and STING in tumors

**DOI:** 10.1172/JCI181044

**Published:** 2024-12-02

**Authors:** Chuqing Zhang, Zhenji Deng, Jiawei Wu, Cong Ding, Zhe Li, Zhimin Xu, Weipeng Chen, Kaibin Yang, Hanmiao Wei, Tingxiang He, Liufen Long, Jun Ma, Cheng Xu, Xiaoyu Liang

**Affiliations:** 1 State Key Laboratory of Oncology in South China, Collaborative Innovation Center of Cancer Medicine, Guangdong Key Laboratory of Nasopharyngeal Carcinoma Diagnosis and Therapy, Guangdong Provincial Clinical Research Center for Cancer,; 2 Department of Radiation Oncology,; 3 Department of Pathology,; 4 Department of Ultrasound and Electrocardiography,; 5 Department of Nuclear Medicine, and; 6 Department of Medical Imaging, Sun Yat-sen University Cancer Center, Guangzhou, China.

**Keywords:** Immunology, Oncology, Cellular immune response, Radiation therapy

## Abstract

Type I IFNs (IFN-Is) induced by radiotherapy (RT) are critical for its efficacy, while the mechanism by which tumor cells inhibit IFN-I production remains largely unsolved. By an unbiased CRISPR screen, we identified hemeoxygenase 1 (HO-1) as an RT-related regulator of IFN-I production. Mechanistically, the ER-anchored, full-length HO-1 disrupted stimulator of IFN genes (STING) polymerization and subsequent coat protein complex II–mediated (COPII-mediated) ER-Golgi transportation, leading to hampered activation of downstream signaling. This process was exacerbated by the upregulation of HO-1 expression under RT. Importantly, RT also induced HO-1 cleavage. Cleaved HO-1 underwent nuclear translocation, interacted with cyclic GMP-AMP synthase (cGAS), and inhibited its nuclear export upon irradiation, leading to suppressed 2′3′-cyclic GMP-AMP (cGAMP) production. Furthermore, we revealed that HO-1 inhibitors could enhance local and distant tumor control of RT in vivo. Clinically, higher HO-1 expression was associated with a poorer prognosis and earlier tumor relapse after RT in multiple types of patient tumors. Collectively, through comprehensive inhibition of the cGAS/STING pathway, HO-1 strongly inhibited RT-induced IFN-I production, and targeting HO-1 was shown to be a promising RT-sensitizing therapeutic strategy.

## Abstract 2

By redistributing both cGAS and STING, HO-1 is a key negative radiotherapy (RT)-related regulator of IFN-Is production.

## Graphical abstract

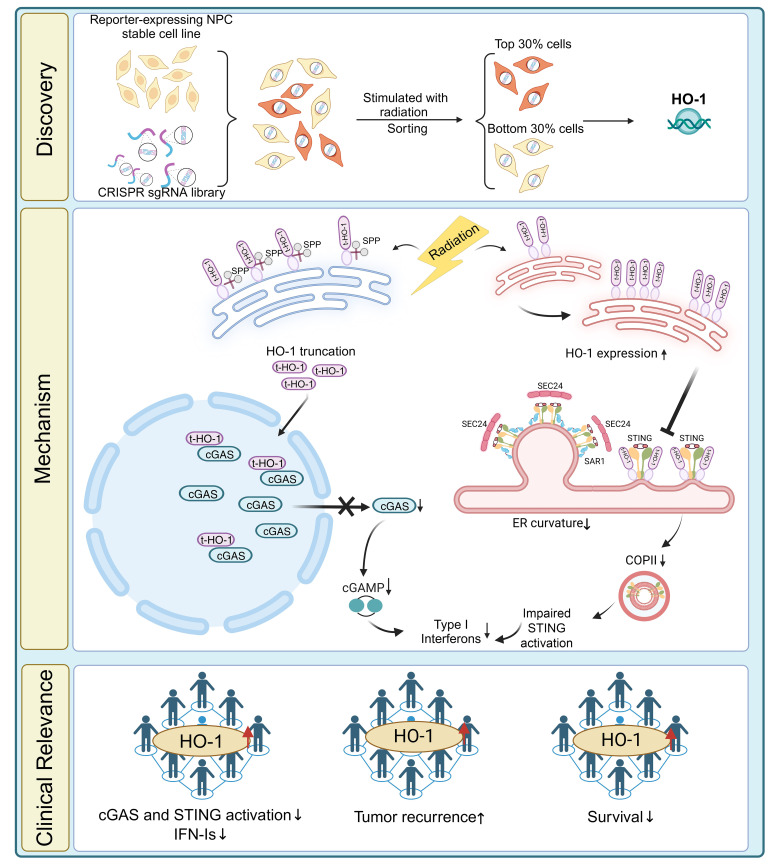

## Introduction

The mechanism of radiotherapy (RT) against cancer is thought to rely mainly on its cytotoxic effect on tumor cells (1). However, increasing evidence indicates that effective evocation of antitumor immunity is crucial for determining RT efficacy. Nevertheless, the regulatory effect of RT on immunity is 2 sided, which induces 2 completely opposite effects: activating or suppressing antitumor immunity (2–5). Type I IFNs (IFN-Is) induced by RT are key factors in activating antitumor immunity (6). The production of IFN-Is depends on the activation of pattern recognition receptors (PRRs), which detect unique pathogen-associated molecular patterns (PAMPs) or damage-associated molecular patterns (DAMPs) to trigger intracellular signaling cascades (7). In the context of RT, DNA breakage yields a large amount of DNA fragments, acting as DAMPs to activate cyclic GMP-AMP synthase/stimulator of IFN genes (cGAS/STING) signaling and IFN-I production (8, 9). Multiple negative feedback mechanisms restrain cGAS/STING signaling to maintain immune homeostasis; however, these findings are mainly derived from studies regarding infection or immune cells (10, 11). For tumor cells, excessive inhibition of this signaling is one of the important factors leading to immune escape. In tumor treatment, especially RT, the unique inhibitory regulatory pattern of cGAS/STING signaling in tumor cells remains to be further elucidated.

The subcellular localization of cGAS and STING plays a decisive role in their functions (12, 13). cGAS is considered a cytosolic DNA sensor, but recent studies have uncovered its nuclear location (14). Nuclear cGAS has effects other than sensing DNA, and its catalytic activity is suppressed (13, 15). Inhibition of nuclear export markedly inhibits the catalytic activity of cGAS, suggesting that nuclear export of cGAS critically regulates the transition between its noncanonical and canonical functions as a nuclear protein and a cytoplasmic protein, but the regulatory mechanism remains largely unknown. STING, in its resting state, is an ER-resident protein (16, 17). Activated STING polymerizes and translocates from the ER to the Golgi apparatus; polymerized STING then serves as a platform to recruit and activate critical signaling cascades to transcribe IFN-Is (18, 19). STING activation is a stabilized state of STING oligomerization led by ligands, typically 2′3′-cyclic GMP-AMP (cGAMP) (20). Essentially, STING oligomerization also directly affects its own transport and distribution (21). Therefore, inhibiting STING oligomerization disrupts its function and signaling, but the underlying mechanisms remain largely unexplored.

In this study, we conducted an unbiased CRISPR/Cas9 screen targeting metabolism related genes and discovered that heme oxygenase 1 (HO-1) was a key negative regulator of RT-induced IFN-I signaling. HO-1 is a type II detoxifying enzyme that catalyzes the rate-limiting step in heme degradation, producing carbon monoxide (CO), free iron, and biliverdin (22). However, we demonstrated that HO-1 inhibited IFN-I production by comprehensively disturbing the distribution of cGAS and STING during RT, independent of its enzymatic activity. Furthermore, irradiation induced HO-1 expression and promoted its cleavage, thereby establishing the inherent connection between RT and HO-1. Thus, targeting HO-1 to boost RT-induced IFN-Is holds promises for more effective and comprehensive RT-based therapy.

## Results

### A metabolic CRISPR/Cas9 screen identifies HO-1 as a potent IFN-I production inhibitor in response to RT.

**Figure 1:**
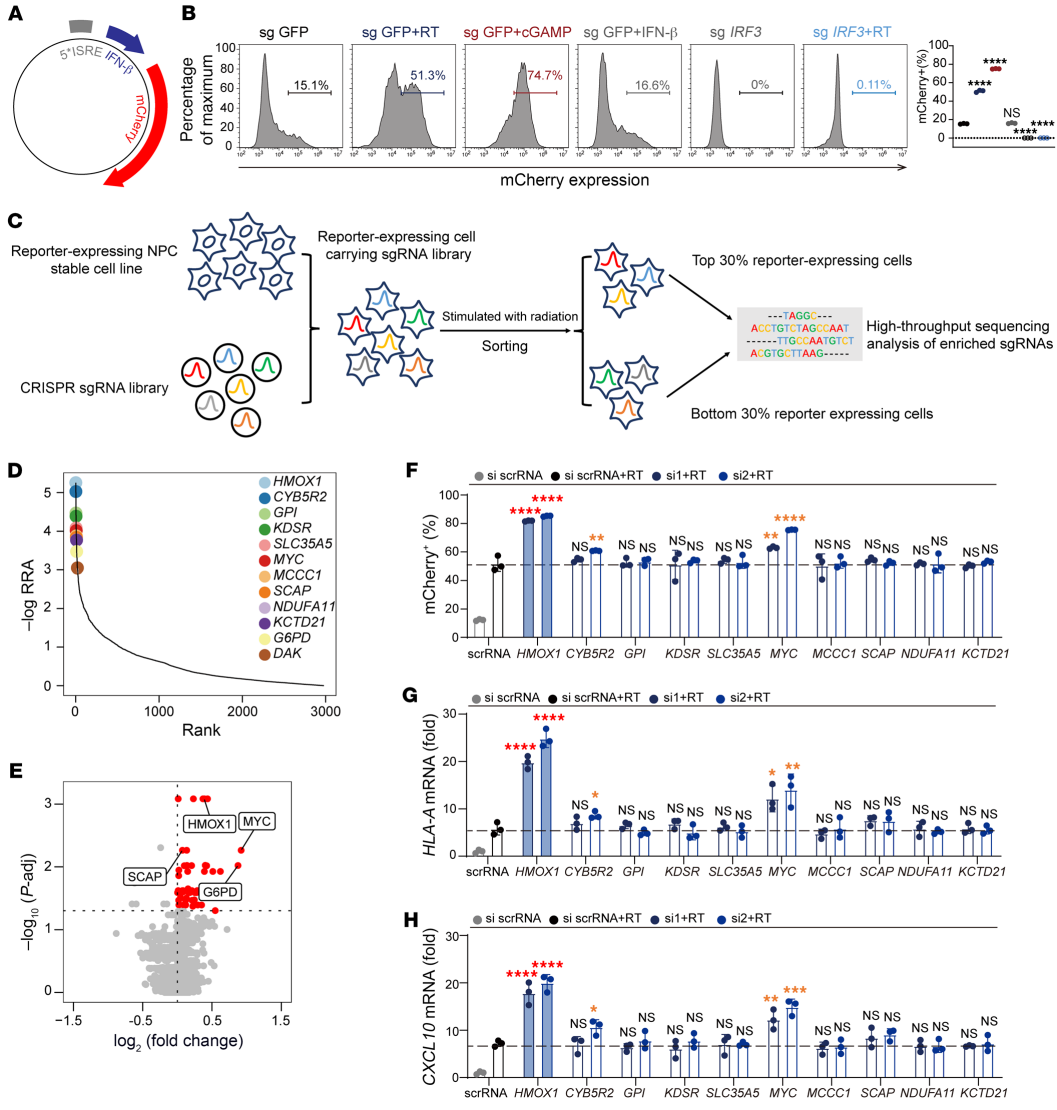
A metabolic CRISPR/Cas9 screen identifies HO-1 as a potent IFN-I production inhibitor in response to RT. (**A**) Schematic overview of the mCherry reporter construct. mCherry expression is driven by ISREs followed by the IFN-β promoter. (**B**) Control HK1 cells and *IRF3*-KO HK1 cells were stimulated with radiation, cGAMP (10 μM), or IFN-β (100 ng/mL). mCherry reporter expression was further analyzed by flow cytometry. (**C**) Overview of the CRISPR screen. Reporter-expressing HK1 cells were transduced with the sgRNA library. After radiation, the cells were sorted by flow cytometer according to mCherry expression and divided into the highest 30% and the lowest 30% mCherry-expressing populations. Genomic DNA from the sorted cells was deep sequenced to reveal gRNA enrichment. (**D**) Distribution of the RRA score of the top hits enriched in the mCherry high expression group versus the low expression group. (**E**) Volcano plot illustrating the important candidates based on the comparison of high mCherry-expressing group versus the low mCherry-expressing group. *P*-adj, adjusted *P* value. (**F**–**H**) Reporter expression (**F**), *HLAA* (**G**), and *CXCL10* (**H**) mRNA levels after knocking down the top 10 candidates in a post-RT CRISPR screen. *P < 0.05, **P < 0.01, ***P < 0.001, and ****P < 0.0001, by 1-way ANOVA (**B** and **F**–**H**). Data are shown as the mean ± SD (*n* = 3 biologically independent samples).

Tumor metabolism and related enzymes are intimately connected with the IFN-mediated immune response (23, 24). To identify key metabolic genes regulating IFN-I production during RT, we used a metabolic gene-KO library targeting 2,981 genes with 29,790 single-guide RNAs (sgRNA) for a systematic screen (25). First, the nasopharyngeal carcinoma (NPC) cell line HK1 was stably integrated with a reporter cassette containing mCherry driven by IFN-stimulatory response elements (ISREs) and the IFN-β promoter to visualize and quantify IFN-I production (Figure 1A). Then, the reporter cells were treated with RT or cGAMP, demonstrating the high specificity and efficiency of this system (Figure 1B). Moreover, as a negative control, KO of *IRF3* almost abolished mCherry expression induced by irradiation (Figure 1B). Next, reporter-expressing cells were transduced with the metabolic CRISPR/Cas9-KO library, treated with RT, and sorted by flow cytometry on the basis of the highest and lowest 30% mCherry fluorescence signals for deep sequencing (Figure 1C and Supplemental Figure 1A; supplemental material available online with this article; https://doi.org/10.1172/JCI181044DS1).

We used the robust rank aggregation (RRA) algorithm and identified several genes in the hyperresponsive population (Figure 1, D and E), among which *MYC*, *SCAP*, *G6PD*, and *DAK* were previously reported to inhibit IFN-I production and downstream signaling (26–29), supporting the validity of our screening strategy. We then validated the top 10 genes with a siRNA (Supplemental Figure 1B). *HMOX1* (HO-1), which ranked as the top gene, had the most potent effect on inhibition of IFN-β production after RT, whereas the other genes, except *MYC*, showed weak effects (Figure 1, F–H, and Supplemental Figure 1C).

Meanwhile, HO-1 had higher expression in patients with NPC who had tumor recurrence after RT, while none of the other top 10 genes in the CRISPR screen were upregulated in the RNA-Seq analysis (Supplemental Figure 1D). Thus, we selected HO-1 for further investigation.

### HO-1 inhibits RT-mediated IFN-I production independent of its enzymatic activity.

**Figure 2:**
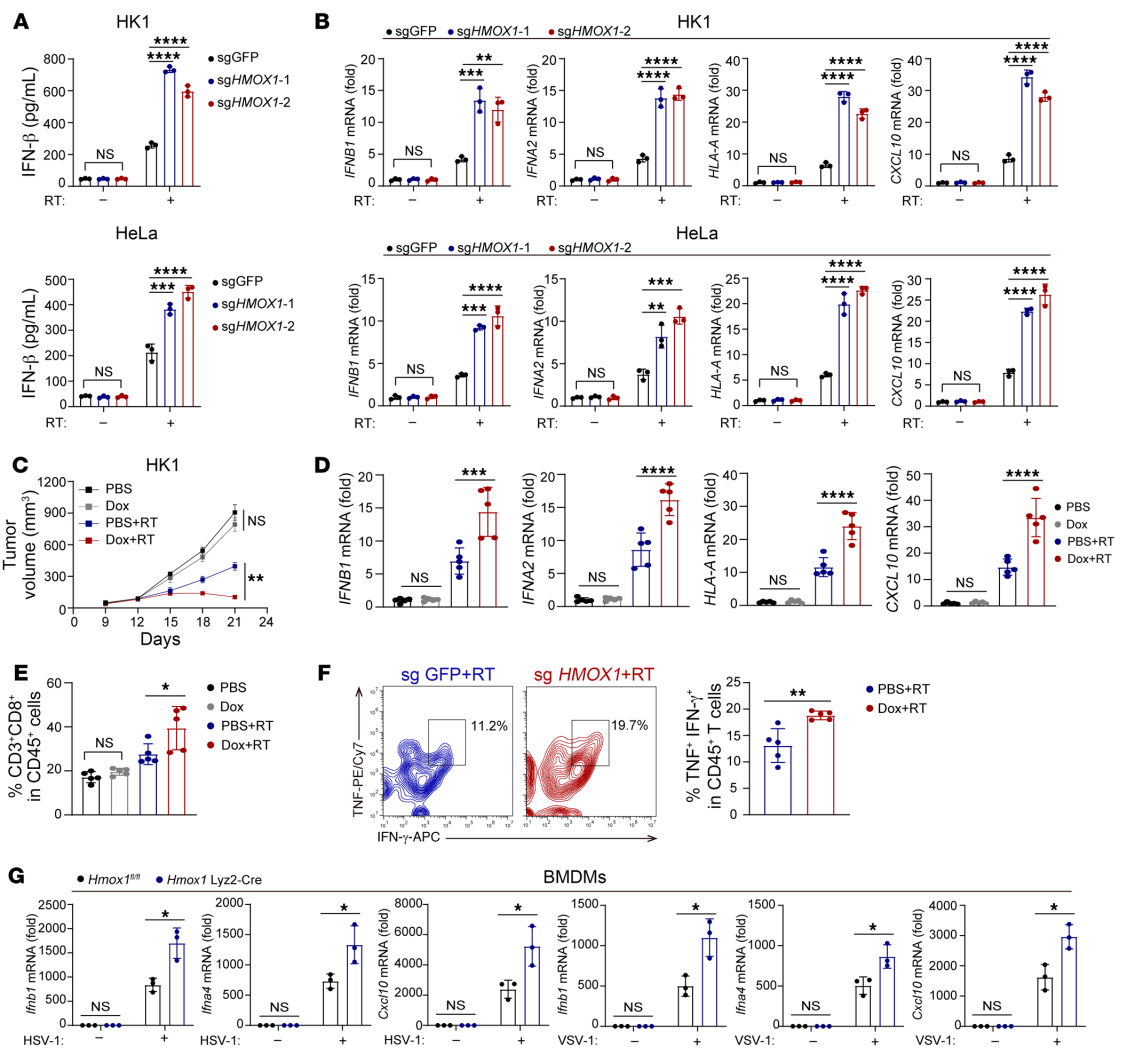
HO-1 inhibits RT-mediated IFN-I production. (**A**) ELISA for IFN-β content in the supernatant of control or *HMOX1*-KO cells before and after RT. (**B**) Typical IFN-Is and ISGs mRNA levels of control or *HMOX1*-KO cells before and after RT. (**C**) Tumor growth of *HMOX1*-inducible knockdown HK1 tumors in HuHSC-NCG mice, following with or without RT (10 Gy) (*n* = 5 in each group). (**D**–**F**) RT-qPCR analysis for mRNA levels of typical IFN-I and ISG genes (**D**) and flow cytometric analysis of CD8^+^ T cell infiltration (**E**) and TNF-α and IFN-γ expression of CD8^+^ T cells (**F**) in the HK1 model (*n* = 5 in each group). APC, allophycocyanin. (**G**) *Ifnb1, Ifna4*, and *Cxcl10* mRNA levels in BMDMs from *Hmox1^fl/fl^* and *Hmox1^fl/fl^* Lyz^Cre/Cre^ mice. BMDMs were infected with HSV-1 or VSV. Data are shown as the mean ± SD (**A**, **B**, and **D**–**G**) and the mean ± SEM (**C**). *P < 0.05, **P < 0.01, ***P < 0.001, and ****P < 0.0001, by 1-way ANOVA (**A**, **B**, **D**, and **E**), 2-way ANOVA (**C**), and unpaired, 2-tailed, Student’s *t* test (**F** and **G**). *n* = 3 biologically independent experiments, unless otherwise stated. Dox, doxycycline.

To further explore the role of HO-1 in inhibiting IFN-I production under RT, we constructed *HMOX1*-KO HK1 and HeLa cell lines using CRISPR/Cas9 (Supplemental Figure 2A). After RT, compared with control cells, we observed increased IFN-β levels in *HMOX1*-KO cells (Figure 2A). Similar results were observed in the human prostate cancer, breast cancer, and fibrosarcoma cell lines DU145, MDM-MB-231, and HT-1080, respectively (Supplemental Figure 2B). Additionally, the mRNA levels of typical IFN-Is (*IFNB1* and *IFNA2*) and IFN-stimulated genes (ISGs) (*HLAA* and *CXCL10*) were upregulated in *HMOX1*-KO cells after RT (Figure 2B).

Next, we sought to verify the in vivo effect of HO-1 on the regulation of IFN-I production and correlated RT efficacy. To this end, we constructed 2 cold tumor models (low CD8^+^ T cell infiltration) with the murine melanoma cell line B16 and the breast cancer cell line 4T1, and then a hot tumor model (high CD8^+^ T cell infiltration) with the colon adenocarcinoma cell line MC38. To eliminate confounding effects of stable *HMOX1* KO on tumor progression, we established *Hmox1*-inducible knockdown cell lines based on a doxycycline-inducible shRNA expression system (Supplemental Figure 2C). We found that the induction of *Hmox1* knockdown enhanced RT efficacy, leading to decreased tumor volumes in the B16, MC38, and 4T1 models (Supplemental Figure 2D). Moreover, knockdown of *Hmox1* also promoted intratumoral IFN-I production in vivo (Supplemental Figure 2E). More important, we also validated our findings in a model based on a human cancer cell line and a reconstructed immune system. Transgenic HK1 cells with inducible *HMOX1* knockdown were injected into HuHSC-NCG mice, which were constructed by implanting human hematopoietic stem cells after bone marrow ablation. Under RT, *HMOX1* knockdown resulted not only in delayed tumor growth (Figure 2C) but also elevated intratumoral IFN-I and ISG expression (Figure 2D). CD8^+^T infiltration and function were also boosted after knocking down *HMOX1* (Figure 2, E and F).

To further validate the role of HO-1 in other scenarios except for RT, we used virus infection as another essential IFN-I inducer and generated *Hmox1^fl/fl^* mice and *Hmox1^fl/fl^* Lyz^Cre/Cre^ mice. HO-1–deficient bone marrow–derived macrophages (BMDMs) exhibited upregulated IFN-I (*Ifnb1* and *Ifna4*) and ISG (*Cxcl10*) expression levels in response to both herpes simplex virus type 1 (HSV-1) and vesicular stomatitis virus (VSV) (Figure 2G and Supplemental Figure 2F). Besides, HO-1 deficiency enhanced the expression of canonical inflammation molecules (*Tnfa* and *Il6*) induced by dsDNA (HSV-1 and HT-DNA) in BMDMs (Supplemental Figure 2, G and H).

**Figure 3:**
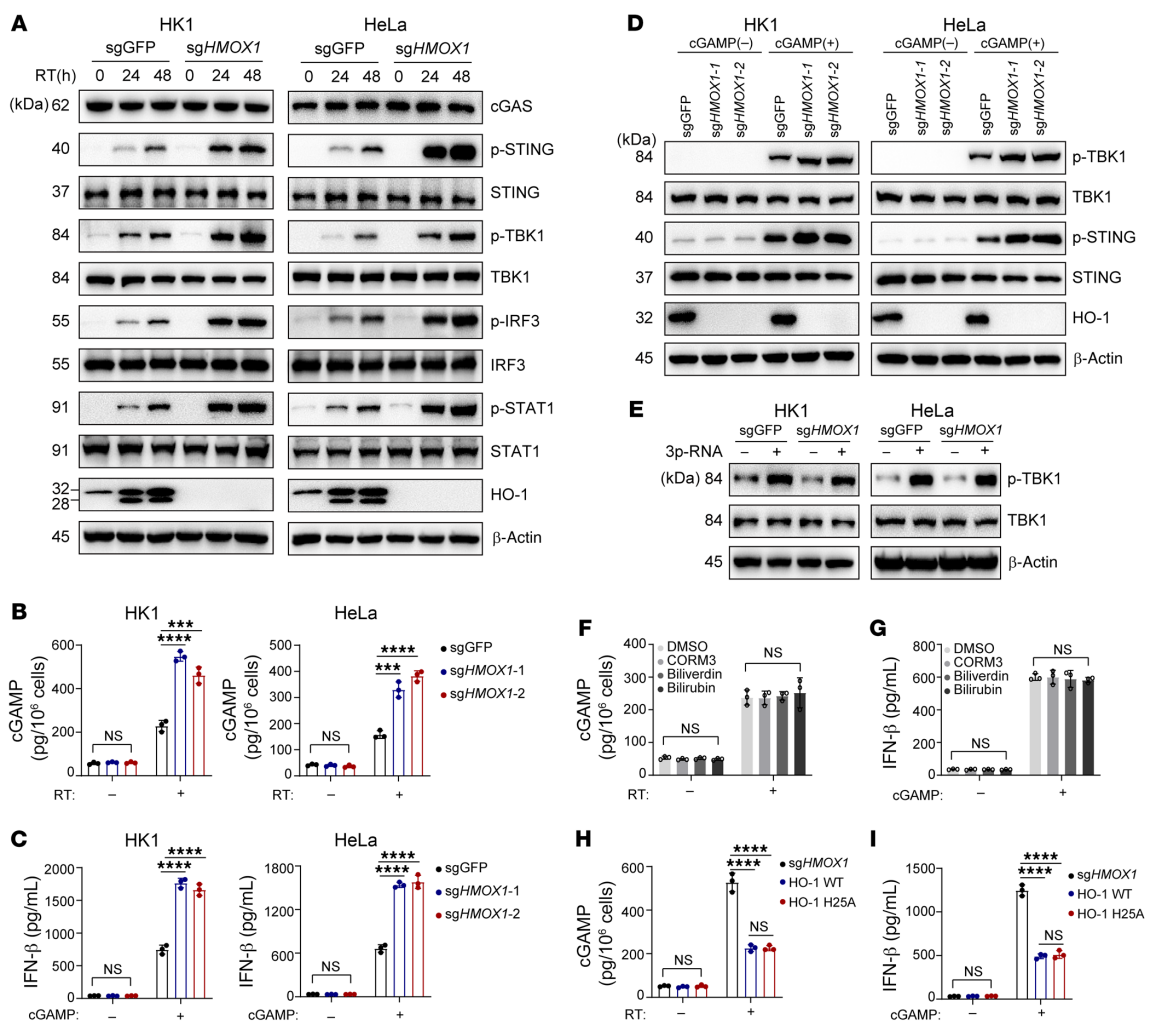
HO-1 inhibits the activity of cGAS and STING under RT independent of its enzymatic activity. (**A**) Immunoblot analysis of essential molecules in IFN-I signaling from control or *HMOX1*-KO cells before and after RT. (**B**) ELISA of cGAMP production of control or *HMOX1*-KO cells before and after RT. (**C**) ELISA of IFN-β production in the supernatant of control or *HMOX1*-KO cells with or without cGAMP stimulation. (**D** and **E**) Immunoblot analysis of the indicated proteins from control or *HMOX1*-KO cells with the indicated treatment. (**F** and **G**) ELISA of cGAMP (**F**) or IFN-β (**G**) production in HK1 cells treated with the indicated metabolites. (**H** and **I**) *HMOX1*-KO HK1 cells were stably transfected with WT HO-1 or HO-1H25A. ELISA of cGAMP (**H**) or IFN-β (**I**) production with or without the indicated stimulation. Data are shown as the mean ± SD. ***P < 0.001 and ****P < 0.0001, by 1-way ANOVA (**B**, **C**, and **F**–**I**). *n* = 3 biologically independent experiments. p-, phosphorylated.

IFN-Is can be induced by various signaling pathways. Specifically, cytosolic RNA/DNA or LPS activates the retinoic acid–inducible gene I–like (RIG-I–like) receptors (RLR/mitochondrial antiviral signaling protein [MAVS]), cGAS/STING, or TLR/ TIR domain–containing adaptor inducing IFN-β (TLR/TRIF) pathways, respectively, to promote IFN-I production (30). Silencing key adaptor proteins (STING, MAVS, and TRIF) in the 3 pathways mentioned above revealed that HO-1 impaired STING-mediated, but not MAVS- or TRIF-mediated, IFN-β production upon irradiation (Supplemental Figure 3, A and B). To further confirm the involvement of cGAS/STING signaling, we examined the functional state of important molecules in the pathway after knocking out *HMOX1* under RT, and found that HO-1 deficiency enhanced the phosphorylation of STING, TBK1, IRF3, and STAT1 (Figure 3A).

The induction of IFN-Is by cGAS/STING signaling involves a chain of protein-protein interactions, the core of which includes cGAS, STING, and TANK-binding kinase 1 (TBK1). In this regard, we further explored the exact molecule inhibited by HO-1 in this pathway. Notably, HO-1 deficiency not only elevated cGAMP production under RT (Figure 3B and Supplemental Figure 3C) but also enhanced IFN-β production under cGAMP treatment (Figure 3C). cGAMP is generated by cGAS from ATP and GTP, with its activity being regulated by dsDNA (31). However, *HMOX1* KO did not affect the amount of cytosolic dsDNA after RT or various types of chemotherapeutics (cisplatin, oxaliplatin, etoposide, or doxorubicin), indicating that HO-1 might directly influence cGAS function (Supplemental Figure 3, D and E). Moreover, *HMOX1* KO markedly promoted cGAMP-mediated STING and TBK1 phosphorylation (Figure 3D and Supplemental Figure 3F), both of which were critical for activating IRF3 to mediate IFN-β transcription. To further clarify the molecule affected by HO-1, we used 5′ppp-RNA, a typical microbe-associated molecular pattern (MAMP) activating TBK1 via the RIG-I/MAVS pathway and found that HO-1 did not affect TBK1 activation in this scenario (Figure 3E), which suggested that STING might be another direct target of HO-1.

We subsequently investigated whether the effect of HO-1 depended on its enzymatic activity. We found that adding exogenous HO-1 metabolites (bilirubin, biliverdin, or CORM3) into the medium of HK1 cells did not affect cGAMP or IFN-β production after RT (Figure 3, F and G). Consistently, the enzymatically inactive HO-1 mutant (HO-1H25A) (32) showed no obvious difference compared with WT HO-1 in affecting cGAMP or IFN-β production under RT or cGAMP treatment (Figure 3, H and I, and Supplemental Figure 3G).

Taken together, these data suggest that HO-1 inhibited the function of both cGAS and STING in tumor cells under RT independent of its enzymatic activity.

### RT induces HO-1 expression and promotes its cleavage.

**Figure 4:**
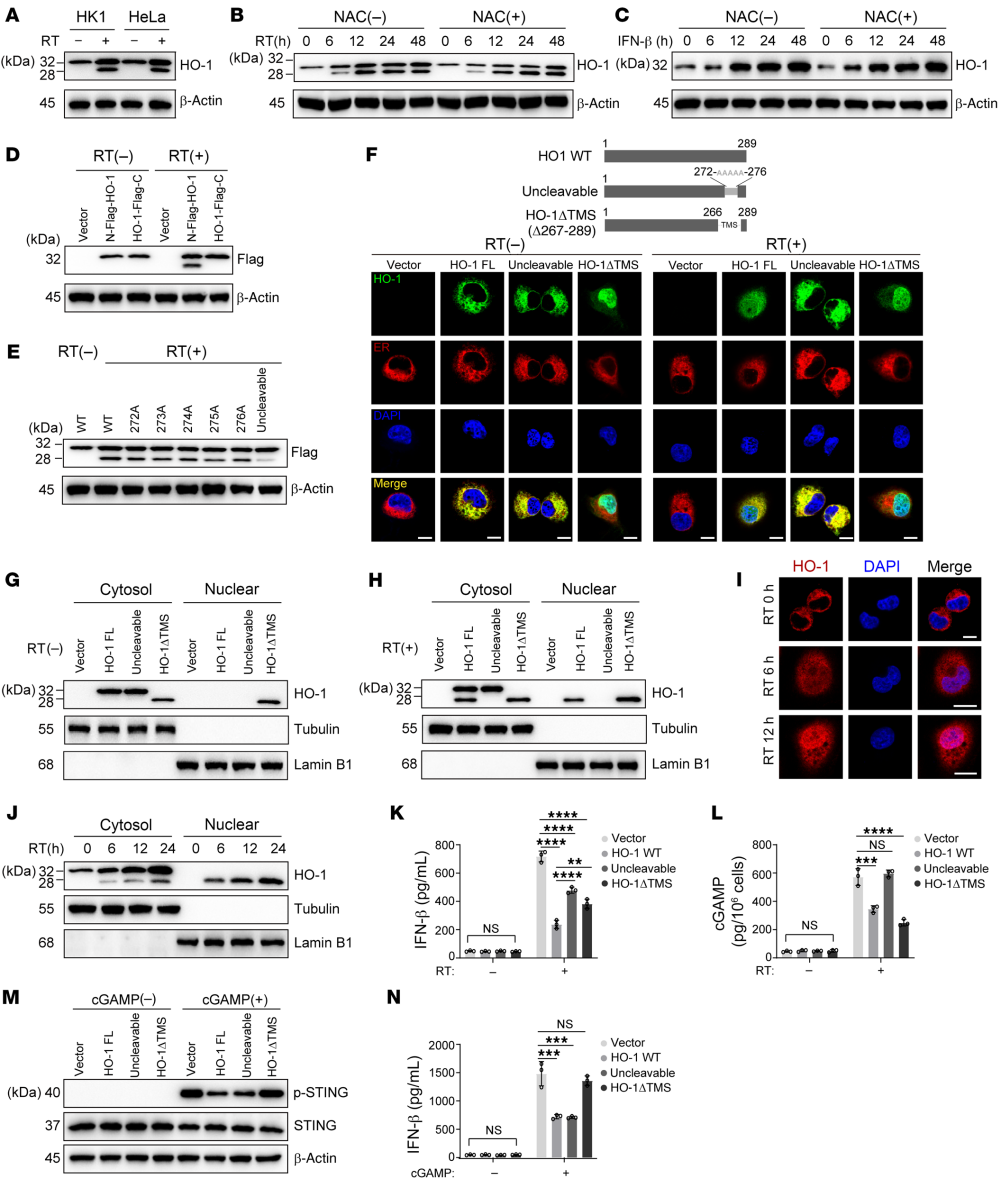
RT induces HO-1 and promotes its cleavage. (**A**) Immunoblot analysis of HO-1 expression and truncation in the indicated cells before and after RT. (**B** and **C**) Immunoblot analysis of HO-1 expression and truncation in HK1 cells after RT (**B**) or IFN-β treatment (**C**) combined with or without NAC treatment. (**D** and **E**) Immunoblot analysis of Flag–HO-1 expression in HK1 cells before and after RT. (**D**) Flag tag was fused to N-terminus or C-terminus of HO-1, respectively. (**E**) Mutating S272-F276 of HO-1 individually or mutating all 5 amino acids between S272 and F276. (**F**) Subcellular distribution (ER and nucleus) of full-length HO-1, uncleavable HO-1 mutant, cleaved HO-1 (HO-1ΔTMS) in HK1 cells with or without RT. Calreticulin staining for the ER; DAPI staining for the nucleus (scale bars: 10 μm). FL, full-length. (**G** and **H**) Nuclear and cytoplasmic protein extraction experiment was performed to determine the cellular localization of exogenous HO-1 or its mutants before (**G**) and after (**H**) RT in HK1 cells. (**I**) Subcellular distribution of endogenous HO-1 was determined with immunofluorescence staining in HK1 cells stimulated with RT (scale bars: 10 μm). (**J**) Nuclear and cytoplasmic protein extraction experiment was performed to determine the cellular localization of endogenous HO-1 at the indicated time point of RT in HK1 cells. (**K** and **L**) *HMOX1*-KO HK1 cells were stably transfected with the indicated HO-1 mutants. With or without RT, cGAMP (**K**) and IFN-β (**L**) production was determined with ELISA. (**M** and **N**) *HMOX1*-KO HK1 cells were stably transfected with indicated HO-1 mutants. With or without cGAMP, STING activation was determined with immunoblot analysis (**M**), and IFN-β production was determined by ELISA (**N**). Representative data from 1 experiment are shown (*n* = 3 biologically independent experiments). *P < 0.05, **P < 0.01, ***P < 0.001, and ****P < 0.0001, by 1-way ANOVA (**K**, **L**, and **N**). Data are shown as the mean ± SD.

Notably, we observed that RT had a direct effect on HO-1, resulting in increased HO-1 expression and cleavage in all 5 tumor cell lines (Figure 4A and Supplemental Figure 4A); and the induction of HO-1 expression by RT was dose dependent below 10 Gy (Supplemental Figure 4B). Among the 5 tumor cell lines, we selected HK1 and MDM-MB-231, along with their corresponding normal epithelial cell lines (NP69 and MCF10A), for further investigation. RT also induced the expression and cleavage of HO-1 in NP69 and MCF10A, suggesting the universality of this phenomenon in both tumor and normal cell lines. Nevertheless, the effect of RT on HO-1 in the normal epithelial cells were weaker than those observed in the paired tumor cell lines (Supplemental Figure 4C).

Then, we explored the mechanism by which RT upregulated HO-1 expression. Considering that RT induces abundant ROS production in tumor cells, we interrogated whether the upregulation of HO-1 was attributed to ROS. Interestingly, adding *N*-acetyl-d-cysteine (NAC) almost abolished HO-1 upregulation in the early phase of RT (0–6 hours after RT); however, HO-1 was still markedly upregulated in the late phase of RT (24–48 hours after RT) (Figure 4B). This prompted us to identify other HO-1–inducing factors besides ROS. To this end, we used pharmacological inhibitors to block several signaling pathways activated by RT (33, 34), including PI3K/AKT, MAPK, NF-κB, Jak/STAT1, and ATR, and found that only the STAT1 inhibitor fludarabine notably abrogated HO-1 upregulation in the late phase of RT (Supplemental Figure 4D). The Jak/STAT1 pathway was activated mainly by IFN signaling. As expected, IFN-β treatment also led to notable HO-1 upregulation (Figure 4C). To further verify the role of IFN-Is, we cotreated tumor cells with NAC and IFN-β and found that NAC did not modulate the effect of IFN-β on HO-1 upregulation (Figure 4C). Therefore, we speculated that HO-1 might exist as an inherent, inflammation-limiting feedback mechanism under RT.

In addition, after RT exposure, 2 immunoreactive bands were unexpectedly observed when using anti–HO-1 antibody, with 1 migrating at 28 kDa and the other migrating at 32 kDa (Figure 4A and Supplemental Figure 4A), implying that RT not only upregulated HO-1 expression, but also induced its cleavage. To identify the sites at which HO-1 was cleaved, a Flag tag was fused to either the N-terminus or C-terminus of HO-1 (N-Flag-HO-1/HO-1-Flag-C). After transfection and RT, immunoblotting showed 2 closely positioned bands when Flag was fused to the N-terminus, while only 1 band appeared when Flag was fused to the C-terminus (Figure 4D), suggesting that HO-1 was cleaved near the C-terminus. Consistently, previous studies have shown that HO-1 has multiple cleavage sites at the C-terminus, primarily between S272-F276 (32, 35). However, mutating any single site was not sufficient to eliminate the cleavage led by RT, and only mutating all amino acids between S272-F276 resulted in an uncleavable state (Figure 4E). Structurally, the S272-F276 segment is located within the transmembrane domain (266–288 aa) of HO-1, which is responsible for its ER location. We observed that whether HO-1 was truncated from S272 or F276, the distribution for either one was similar to the transmembrane domain fully truncated mutant. Specifically, cleaved HO-1 no longer resided on the ER; instead, it was mostly distributed in the nucleus, with a small portion found in the cytoplasm (Supplemental Figure 4E). To clarify the relationship between RT and the changes in HO-1 distribution, we further analyzed the distribution of 3 forms of HO-1, including WT, uncleavable, and cleaved (HO-1ΔTMS), before and after RT. Before RT, WT and uncleavable HO-1 were only located on the ER, whereas the cleaved counterpart was distributed both in the nucleus and the cytosol but not on the ER (Figure 4F). After RT, WT HO-1 appeared to have nuclear distribution and simultaneously displayed 3 distributions: nucleus, cytosol, and ER, while the distribution of uncleavable and cleaved HO-1 (HO-1ΔTMS) did not change and was consistent with that observed before RT (Figure 4F). These observations were further confirmed by nuclear and cytoplasmic protein extraction experiments (Figure 4, G and H). Consistently, endogenous nucleus-located HO-1 was observed only after RT in the truncated form (Figure 4, I and J, and Supplemental Figure 4, F and G). Together, these data indicated that RT-mediated HO-1 cleavage destroyed its transmembrane domain and led to redistribution.

Rhomboid serine proteases, γ-secretases, and signal peptide peptidase (SPP) are 3 major intramembrane protease families (36). By pretreatment with specific inhibitors [DCI, DAPT and (Z-LL)_2_-ketone], we discovered that only the SPP inhibitor suppressed the cleavage of HO-1 under RT (Supplemental Figure 4H), which was accompanied by increased cGAMP and IFN-β production (Supplemental Figure 4I). In line with this, both cleaved and uncleavable HO-1 inhibited IFN-β production under RT (Figure 4K). Previously, we had already identified that WT HO-1 inhibited both cGAS and STING activity. With a similar method, we found that cleaved HO-1 (HO-1ΔTMS) suppressed cGAMP production (Figure 4L), while uncleavable HO-1 inhibited IFN-β production and STING activation after cGAMP treatment, without affecting cGAMP production under RT (Figure 4, M and N). This indicated that cleaved HO-1 acted on cGAS and uncleavable, ER-locked HO-1 acted on STING.

To conclude, RT induced HO-1 expression and promoted its cleavage. Different forms (cleaved or WT) of HO-1 suppressed the function of cGAS or STING.

### Cleaved HO-1 directly interacts with cGAS and inhibits its nuclear export.

**Figure 5:**
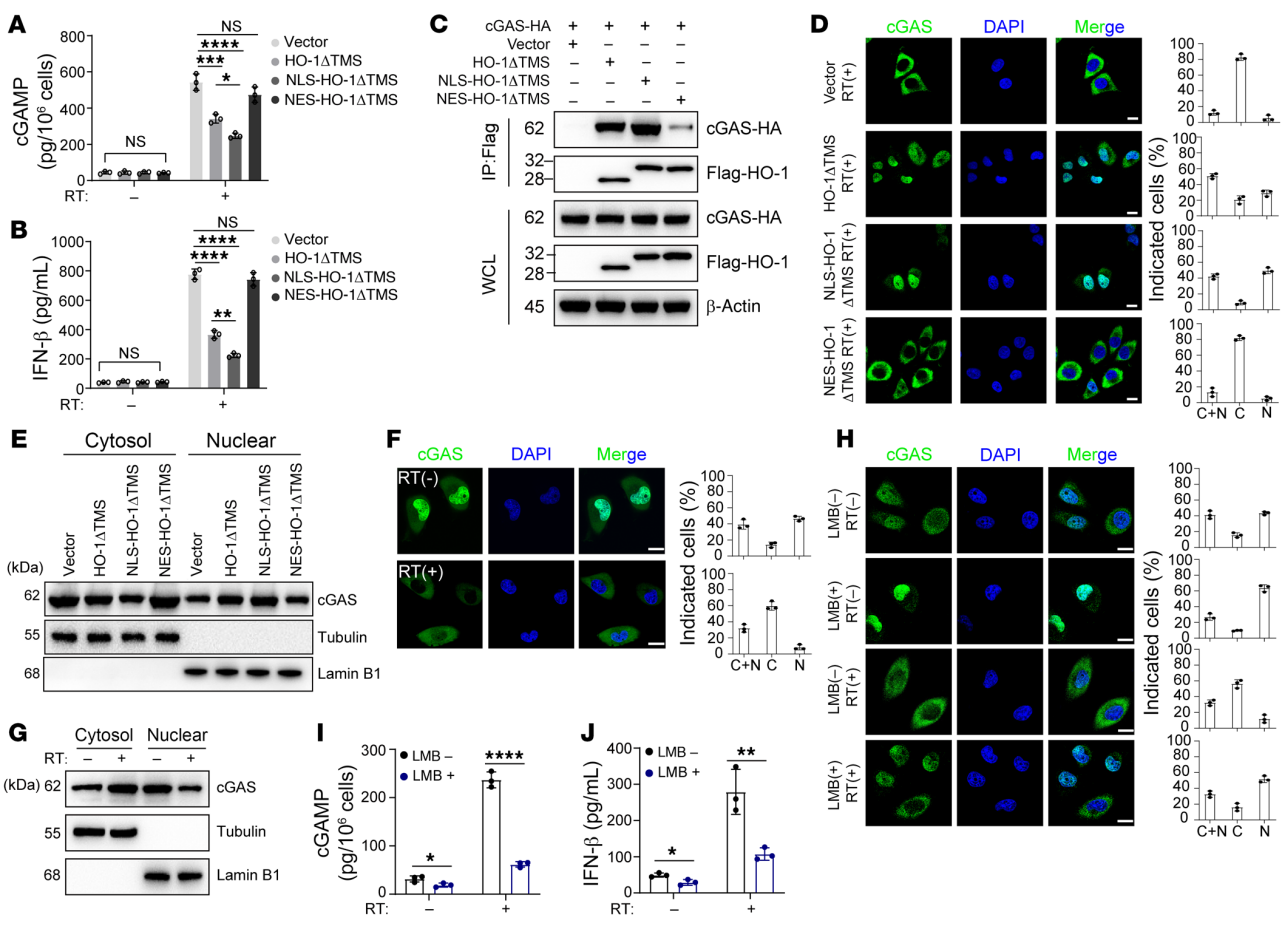
Cleaved HO-1 inhibits the nuclear export of cGAS. (**A**–**E**) *HMOX1*-KO HK1 cells were stably transfected with cleaved HO-1 (HO-1ΔTMS), exclusively nucleus-located cleaved HO-1 (NLS-HO-1ΔTMS), or exclusively cytoplasm-located cleaved HO-1 (NES-HO-1ΔTMS) individually. (**A** and **B**) ELISA of cGAMP (**A**) or IFN-β (**B**) production before and after RT. (**C**) The interaction of Flag-tagged HO-1 mutants and HA-tagged cGAS in HEK293T cells was analyzed by immunoprecipitation under RT. WCL, whole-cell lysate. (**D**, **F**, and **H**) Subcellular distribution (cytoplasm and nucleus) of cGAS was determined by immunofluorescence staining of HK1 cells with the indicated mutants or RT stimulation (scale bars: 10 μm). The percentages of cells (*n* = 200) in the nucleus, cytoplasm, or both the cytoplasm and nucleus were calculated. (**E** and **G**) The cytoplasmic and nuclear protein fractions were extracted for immunoblot analysis to determine the subcellular localization of cGAS in HK1 cells with the indicated mutants or RT stimulation. (**I** and **J**) ELISA of cGAMP (**I**) or IFN-β (**J**) production before and after RT (related to Figure 5H). Data are shown as the mean ± SD. *P < 0.05, **P < 0.01, ***P < 0.001, and ****P < 0.0001, by 1-way ANOVA (**A** and **B**) and unpaired, 2-tailed Student’s *t* test (**I** and **J**). All representative data from 1 experiment are shown (*n* = 3 biologically independent experiments). N, predominantly in the nucleus; C, predominately in the cytoplasm; C+N, evenly distributed in the nucleus and cytoplasm.

Next, we sought to elucidate the mechanism by which cleaved HO-1 regulates cGAS activity. Redistribution of cellular positions was a prominent change in cleaved HO-1 compared with the WT counterpart. Meanwhile, cGAS is distributed to both the nucleus and cytoplasm (37), so we first explored whether distribution is important for HO-1 in regulating cGAS. To this end, the nuclear location signal (NLS) peptide and nuclear export signal (NES) peptide were respectively fused to cleaved HO-1 (HO-1ΔTMS) to change its distribution (Supplemental Figure 5A). We found that increased nuclear distribution of cleaved HO-1 (HO-1ΔTMS) led to stronger inhibitory effects on cGAMP and IFN-β production under RT, while cytoplasm-located cleaved HO-1 (NES-HO-1ΔTMS) nearly abolished these effects (Figure 5, A and B). Furthermore, immunoprecipitation revealed that cleaved HO-1 fused with NLS (NLS-HO-1ΔTMS) showed strong interaction with cGAS under RT (Figure 5C). Additionally, as more cleaved HO-1 entered the nucleus, the nucleus-located cGAS increased after RT (Figure 5, D and E, and Supplemental Figure 5B). Unexpectedly, RT dramatically enhanced cGAS nuclear export (Figure 5, F and G, and Supplemental Figure 5, C and D). Cytoplasmic localization of cGAS is critical for its role as a DNA sensor and in cGAMP synthesis. In our scenario, when cGAS was confined to the nucleus by leptomycin B (LMB), a nuclear export inhibitor, production of both cGAMP and IFN-β was markedly suppressed under RT (Figure 5, H–J).

**Figure 6:**
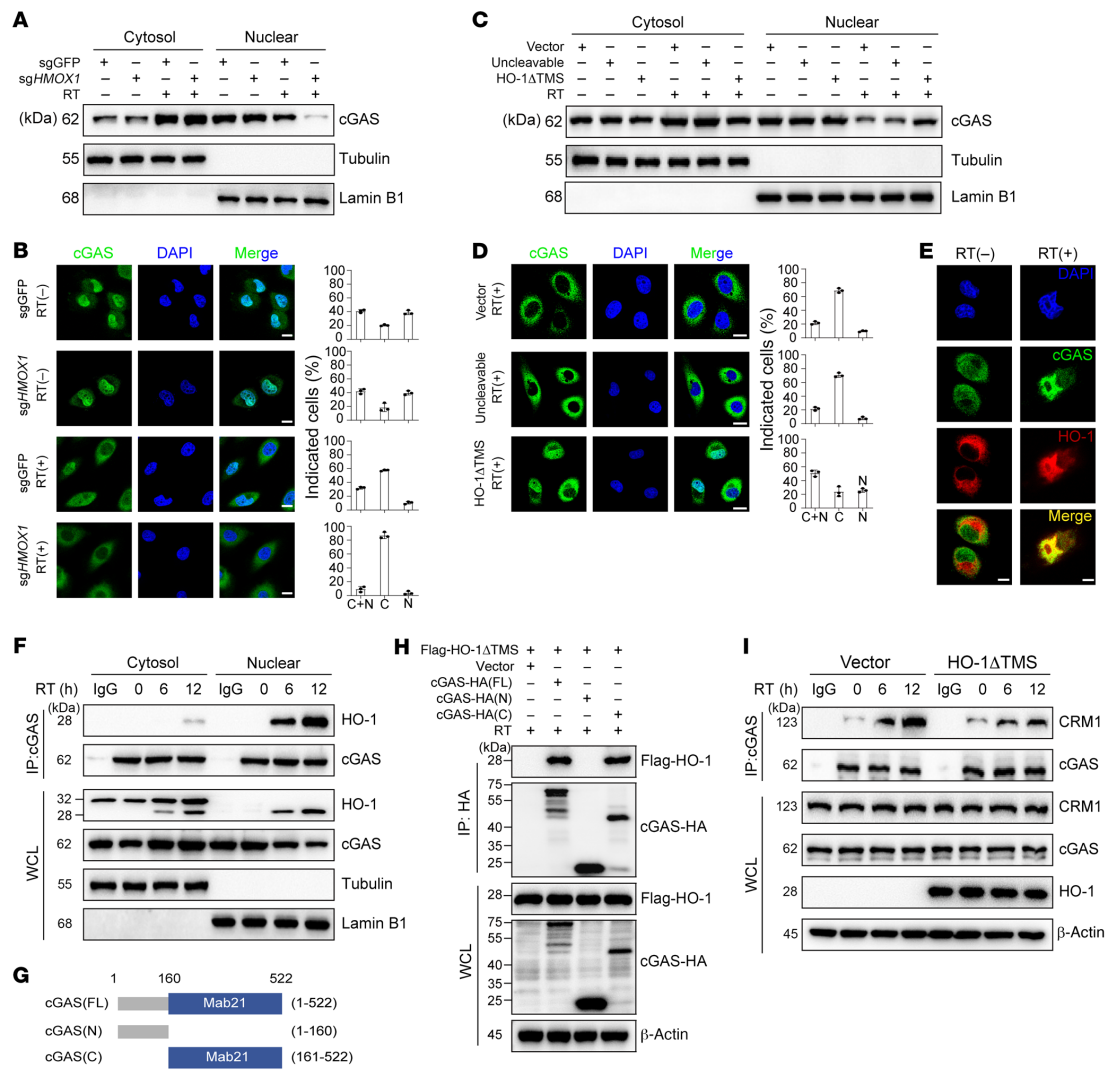
Cleaved HO-1 directly interacts with cGAS in the nucleus. (**A** and **C**) Cytoplasmic and nuclear protein fractions were extracted for immunoblot analysis to determine the subcellular localization of cGAS in HK1 cells with the indicated cell lines and stimulation. (**B** and **D**) Subcellular distribution (cytoplasm and nucleus) of cGAS was determined with immunofluorescence staining of HK1 cells with the indicated cell lines and stimulation (scale bars: 10 μm). The percentages of cells (*n* = 200) in the nucleus, cytoplasm, or both the cytoplasm and nucleus were calculated (**E**) Confocal microscopy images of cGAS and HO-1 in HK1 cells before and after RT (scale bars: 10 μm). (**F**) The cytoplasmic and nuclear protein fractions of HK1 cells at the indicated RT time points were extracted for coimmunoprecipitation. (**G** and **H**) The interaction of HA-tagged full-length cGAS (aa 1–522), N-terminus of cGAS (aa 1–160), C-terminus of cGAS (aa 161–522), and Flag-tagged HO-1ΔTMS in HEK293T cells was analyzed by immunoprecipitation. (**I**) *HMOX1*-KO HK1 cells were stably transfected with cleaved HO-1 (HO-1ΔTMS). The interaction of endogenous cGAS and CRM1 in HK1 cells was analyzed by immunoprecipitation. All representative data from 1 experiment are shown (*n* = 3 biologically independent experiments).

On the basis of the above findings, we speculated that cleaved HO-1 exerts its function by inhibiting cGAS nuclear export. As expected, in HK1 or HeLa cells, we found that before RT, *HMOX1* KO did not influence cGAS nuclear translocation; after RT, HO-1 deficiency increased cGAS nuclear export (Figure 6, A and B, and Supplemental Figure 5, E and F). Consistently, cleaved HO-1 (HO-1ΔTMS) inhibited cGAS nuclear export, but uncleavable HO-1 had a faint effect compared with HO-1–deficient cells after RT (Figure 6, C and D). We previously showed that exogenous HO-1 interacted with cGAS (Figure 5C), and similarly, we found that in endogenous conditions, cleaved HO-1 entered the nucleus and directly interacted with cGAS, and the interaction was gradually enhanced within 12 hours after RT (Figure 6, E and F). To further elucidate the detailed interaction between cleaved HO-1 and cGAS, we generated truncated cGAS mutants by separating cGAS into the N-terminus (1–160 aa) and the C-terminus (161–522 aa) (38) (Figure 6G). Immunoprecipitation revealed that the C-terminus was required for the interaction between cGAS and cleaved HO-1 under RT (Figure 6H). The NES sequence of cGAS resided in the C-terminus (39). CRM1 is the main nuclear export receptor that binds to the NES on target proteins, including cGAS, to mediate their nuclear export. Notably, the interaction between cGAS and CRM1 was enhanced under RT, while reexpression of cleaved HO-1 (HO-1ΔTMS) impaired the interaction (Figure 6I). Collectively, these data suggest that RT induces HO-1 cleavage, which then translocates into the nucleus and disturbs the cGAS-CRM1 interaction to impair RT-induced nuclear export of cGAS.

### HO-1 inhibits STING oligomerization and consecutive ER-to-Golgi translocation by direct interaction.

**Figure 7:**
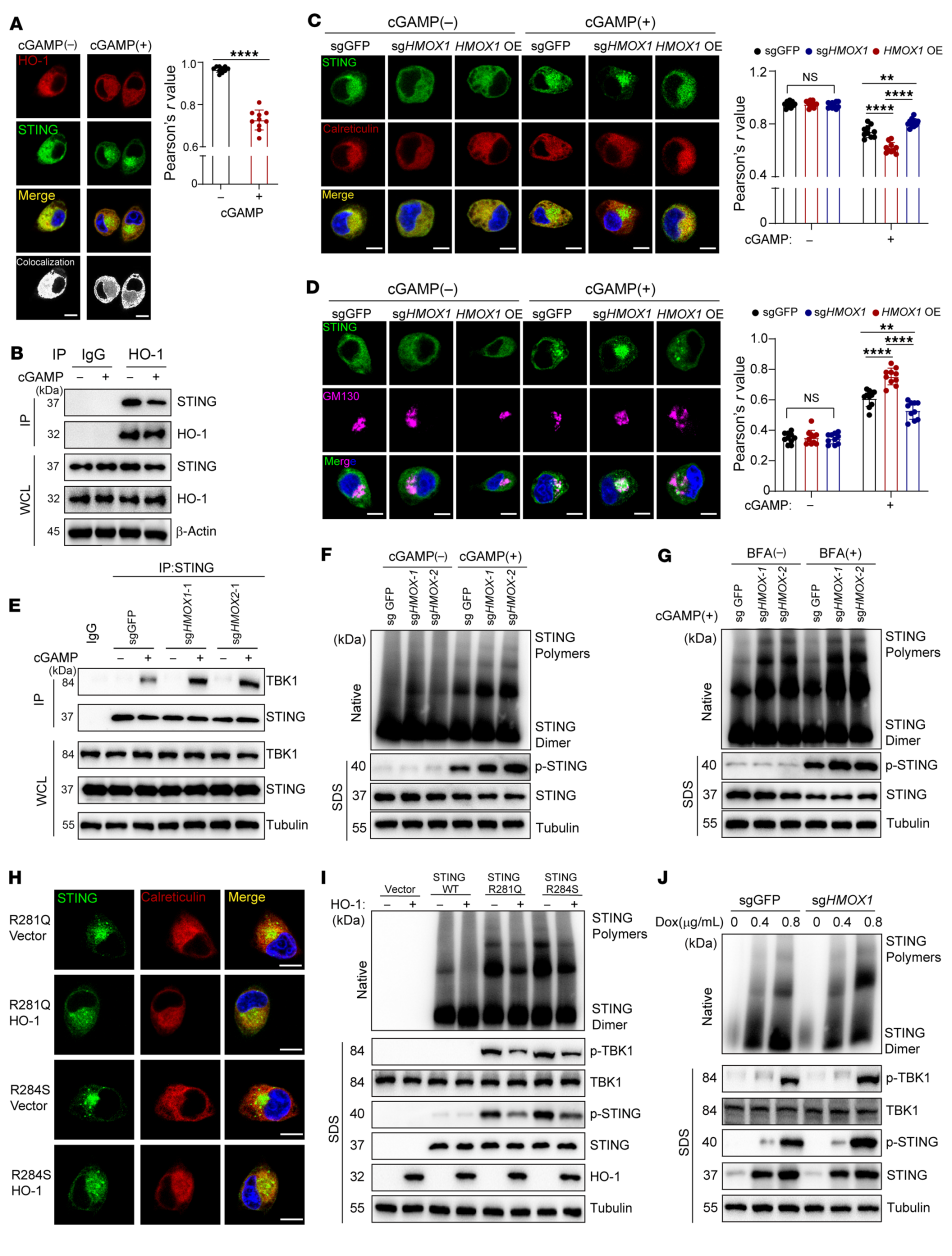
HO-1 inhibits STING oligomerization and consecutive ER-to-Golgi translocation by direct interaction. (**A**) Confocal microscopy images of STING and HO-1 in HK1 cells with the indicated treatment. Pearson’s *r* value was used as a statistical measure to determine the extent of colocalization between HO-1 and STING. (**B**) The interaction of endogenous HO-1 and STING in HK1 cells was analyzed by immunoprecipitation with the indicated treatment. (**C** and **D**) Control, *HMOX1*-KO, and *HMOX1*-overexpressing HK1 cells were stained with anti-STING (**C** and **D**), anti-calreticulin (**C**), and anti-GM130 (**D**) antibodies. Pearson’s *r* value was used as a statistical measure to determine the extent of colocalization between STING and calreticulin or GM130. (**E**) The interaction of endogenous STING and TBK1 in HK1 cells was analyzed by immunoprecipitation with the indicated treatment. (**F** and **G**) STING polymerization in control and *HMOX1*-KO HK1 cells with the indicated treatments, followed by native PAGE and SDS-PAGE. (**H**) HEK293T cells were transfected with the indicated STING mutant plus vector or STING mutant plus HO-1, followed by confocal imaging. (**I**) HEK293T cells were cotransfected with plasmids expressing HO-1 and STING, or its mutants, followed by native PAGE and SDS-PAGE. (**J**) HK1 cells were stably transfected with doxycycline-induced (Dox) STING expression plasmids. After doxycycline treatment at the indicated dose, native PAGE for detection of STING polymers and SDS-PAGE were performed. (**A**) Imaging data were analyzed with Fuji software to reveal colocalization as white dots. (**A**, **C**, and **D**) Pearson’s correlation coefficient was quantified using ImageJ (NIH). *n* = 10 cells (quantified in a blinded manner). Data are shown as the mean ± SD. Scale bars: 10 μm. **P < 0.01 and ****P < 0.0001 by unpaired, 2-tailed Student’s *t* test (**A**) and 1-way ANOVA (**C** and **D**).

We next investigated the mechanism by which HO-1 regulates STING activation. As demonstrated previously, ER-locked, uncleavable HO-1 selectively suppressed STING-mediated IFN-β production (Figure 4, M and N). Immunofluorescence indicated that, in the resting state, STING was completely colocalized with HO-1; after cGAMP treatment, a part of STING aggregated into puncta and lost colocalization with HO-1, while the remaining part still colocalized with HO-1 (Figure 7A). Since resting STING is located in the ER as a dimer, we next explored whether HO-1 exerts its effects by directly interacting with STING. Consistently, immunoprecipitation revealed that HO-1 interacted with STING with or without cGAMP, and the amount of STING bound to HO-1 was reduced after cGAMP addition (Figure 7B). cGAMP-activated STING is transported from the ER to the Golgi apparatus. In this regard, we found that knocking out *HMOX1* further decreased ER-located STING and increased its distribution in the Golgi apparatus (Figure 7, C and D), which could be reversed by reexpression of *HMOX1* (Figure 7, C and D). Therefore, we speculated that HO-1 played a role in restricting STING translocation. To confirm this, we analyzed the interaction between TBK1 and STING, because TBK1 binds mainly to ER-detached, Golgi-located, and oligomeric STING and acts as the key factor to turn on downstream signaling. As anticipated, the interaction between TBK1 and STING was enhanced after knocking out *HMOX1* (Figure 7E).

The formation of STING oligomers is the key step in recruiting the coat protein complex II (COPII), which facilitates the translocation of STING and its spatial redistribution. Therefore, we explored whether HO-1 influences STING oligomerization and found that, essentially, the way cGAMP triggered the STING signaling cascade was by inducing STING oligomerization. In this regard, we found that cGAMP treatment induced stronger STING oligomerization in *HMOX1*-KO cells (Figure 7F). Considering that translocated oligomeric STING would undergo degradation, pretreatment with brefeldin A (BFA) to inhibit ER-Golgi translocation was adopted to avoid potential confounding. When STING was restrained on the ER, KO of *HMOX1* also enhanced its oligomerization (Figure 7G); notably, HO-1 also inhibited baseline STING oligomerization even without cGAMP (Figure 7F). To further verify that HO-1 inhibited STING on the ER, and to rule out the confounding effect of cGAMP uptake, we used 2 other cellular models, in which STING oligomerization and activation occurred automatically. In 1 model, we mutated R281 or R284 amino acids that resided in the polymerization interface of STING (20). After confirming autoactivation (Supplemental Figure 6, A and B), we found that HO-1 overexpression increased ER distribution and weakened the activation and polymerization of these 2 mutants (Figure 7, H and I, and Supplemental Figure 6C). In another model, we developed a doxycycline-induced STING expression system in cGAS-deficient cells, as elevated STING protein on the ER would be advantageous for STING polymerization. Autoactivation was induced when the doxycycline concentration reached 0.6–0.8 μg/mL (Supplemental Figure 6D). We discovered that HO-1 depletion also enhanced STING autoactivation and polymerization (Figure 7J).

Successful COPII vesicle formation is critical for STING to undergo ER-Golgi translocation (21). Here, we found that the interaction between STING and key members of the COPII complex (SAR1A, SEC24C) (40) was enhanced by cGAMP treatment, but impaired by HO-1 (Supplemental Figure 6, E and F). The curvature of the ER membrane is critical for recruiting SAR1 and highly affected by protein aggregation on the membrane. Using a GFP133 membrane curvature probe (21, 41) alongside ER-specific staining, we found that HO-1 deletion promoted ER membrane curvature under cGAMP treatment (Supplemental Figure 6G). Therefore, the presence of HO-1 decreased STING aggregation as well as impaired membrane curvature of the ER, thereby resulting in an unpolymerized, ER-locked state of STING.

**Figure 8:**
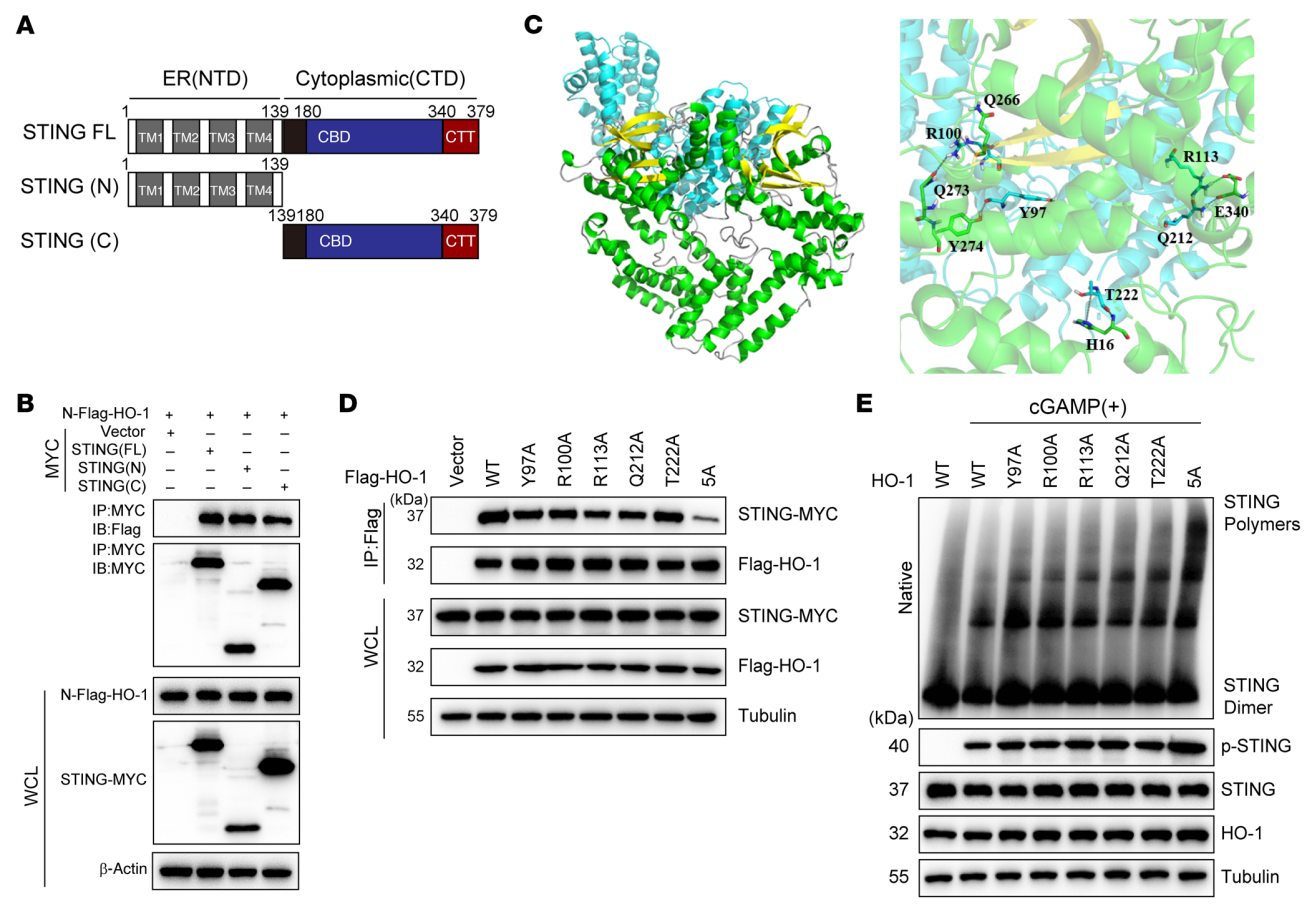
Molecular docking of HO-1 and STING. (**A** and **B**) The interaction of MYC-tagged full-length STING (aa 1–379), N-terminus of STING (aa 1–139), C-terminus of STING (aa 140–379) and Flag-tagged HO-1 in HEK293T cells was analyzed by immunoprecipitation. (**C**) View of binding modes between the STING dimer and the HO-1 dimer based on MD simulations. (**D**) The interaction of MYC-tagged full-length STING and Flag-tagged WT HO-1 or its mutants in HEK293T cells was analyzed by immunoprecipitation. (**E**) HEK293T cells were cotransfected with plasmids expressing STING and HO-1, or its mutants and stimulated or not with cGAMP, followed by native PAGE and SDS-PAGE. (**B**, **D**, and **E**) All representative data from 1 experiment are shown (*n* = 3 biologically independent experiments).

Then, we sought to explore a detailed mechanism by which HO-1 inhibits STING oligomerization. First, by generating truncated STING mutants (N-terminus, 1–139 aa; C-terminus, 140–379 aa; the N-terminus contains the transmembrane segment and the C-terminus facilitates its aggregation; ref. 12), we found that the interaction between HO-1 and STING was not domain selective (Figure 8, A and B). Second, we performed molecular docking and molecular dynamics (MD) simulations. Considering that the basic unit of the STING oligomer was the STING dimer (42), we constructed a STING dimer using Alphafold2 (DeepMind), and the complexes of the STING dimer and HO-1 dimer were constructed with the HDOCK web server (http://hdock.phys.hust.edu.cn/). Through modeling and calculations, we found that HO-1 occupied the interface between 2 STING dimers (residues 273–280 in human STING were verified to be located at the tetramer interface of STING), which was essential for supporting STING tetramerization or hyperpolymerization (Figure 8C). Specifically, residues Thr222, Arg100, Arg100, Tyr97, and Gln212 in HO-1 formed a hydrogen bond with residues His16, Gln266, Gln273, Tyr274, and Glu340 in STING, respectively, and Arg113 in HO-1 formed a salt bridge with residue Glu340 in STING (Supplemental Table 1). To verify the effects of residues in the above docking model, Tyr97, Arg100, Arg113, Gln212, and Thr222 in HO-1 were mutated into alanine to disrupt the formation of a hydrogen bond or a salt bridge. Mutation of each residue variably weakened the binding between HO-1 and STING; and simultaneous mutation of all 5 residues almost abolished the binding (Figure 8D). Corresponding to the strength of each mutant’s binding to STING, STING oligomerization was stronger when the interaction was weaker (Figure 8E); and the variation of binding energy was consistent with the immunoprecipitation (Supplemental Table 2). In line with this finding, the binding energy between homogenous STING dimers was higher than that between HO-1 dimer and the homogenous STING dimer (HO-1 dimer+STING dimer vs. STING dimer+STING dimer: –280.23 kcal/mol vs. –243.68 kcal/mol), implying that the HO-1 dimer+STING dimer had more stable binding (Supplemental Table 3). STING tetramerization formed by the aggregation of 2 STING dimers is the first step in subsequent hyperpolymerization. This prompted us to analyze the effect of HO-1 on STING tetramerization. The binding modes between the STING tetramer and the HO-1 dimer after MD simulations are shown in Supplemental Figure 7A. In all 3 binding systems, the STING tetramer became unstable after binding to the HO-1 dimer according to the analysis of root mean square deviation (RMSD) and root mean square fluctuation (RMSF) (Supplemental Figure 7, B and C). The elevated radius of gyration (Rg) values further illustrated that the dimer-dimer interaction became less tight than the pure STING tetramer after HO-1 binding (Supplemental Figure 7D). Taken together, these data suggest that HO-1 inhibits STING polymerization on the ER and subsequent COPII-mediated translocation from the ER to the Golgi, ultimately impairing STING activation.

### HO-1 inhibition enhances the efficacy and abscopal effect of RT in vivo.

**Figure 9:**
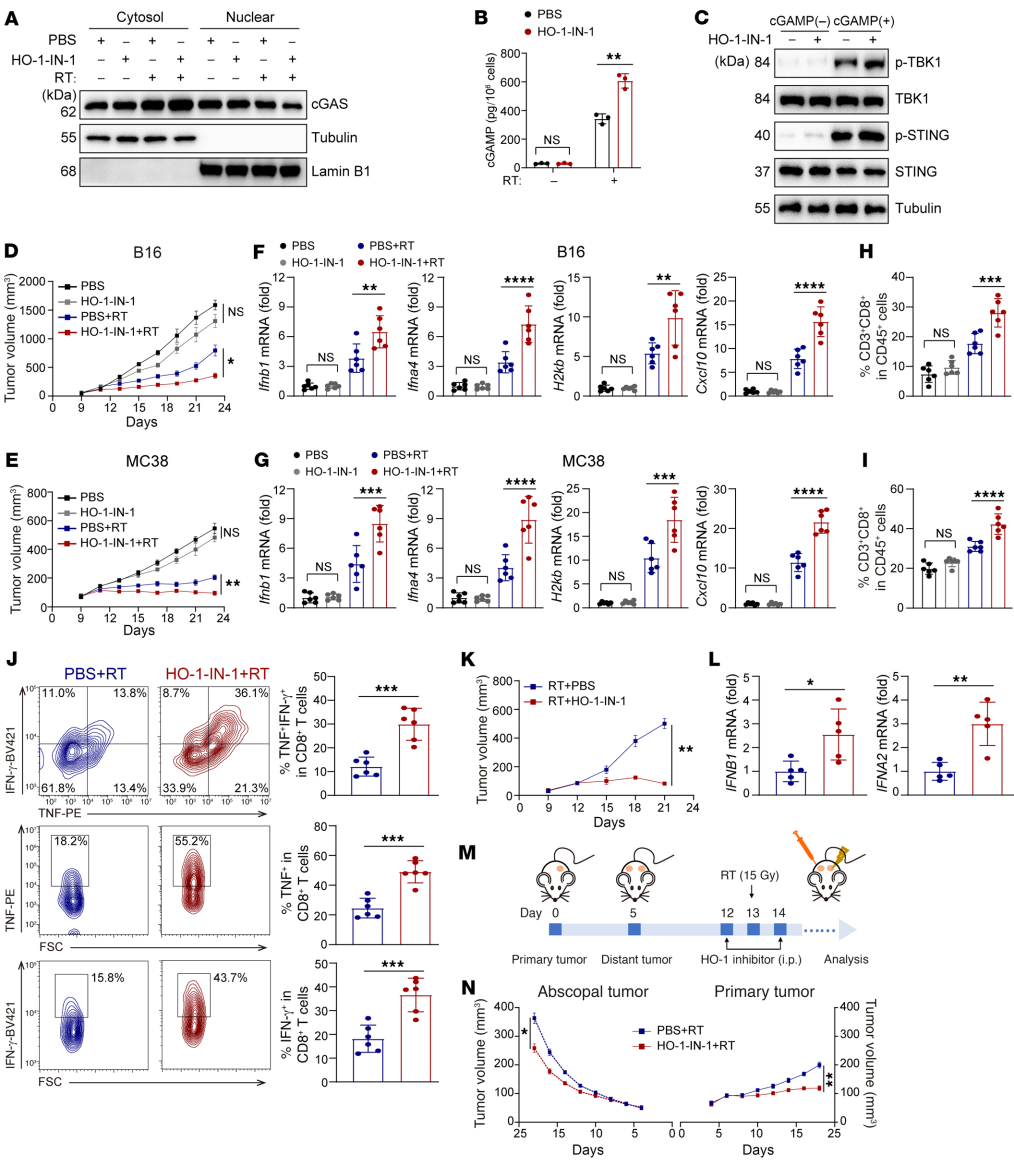
HO-1 inhibitor enhances the efficacy and abscopal effect of RT in vivo. (**A**) The cytoplasmic and nuclear protein fractions were extracted for immunoblot analysis to determine the subcellular localization of cGAS in MC38 cells treated as indicated. (**B**) ELISA of cGAMP production in MC38 cells treated as indicated. (**C**) Immunoblot analysis of STING and TBK1 phosphorylation in MC38 cells treated as indicated. (**D**–**J**) Effect of the HO-1 inhibitor combined with RT on tumor growth (**D** and **E**), mRNA levels of typical IFN-Is and ISGs (**F** and **G**), CD8^+^ T cell infiltration (**H** and **I**), and IFN-γ and TNF-α expression in CD8^+^ T cells (**J**) from B16 (**D**, **F**, and **H**) and MC38 (**E**, **G**, **I**, and **J**) tumors (*n* = 6 in each group). (**K** and **L**) Effect of the HO-1 inhibitor combined with RT on tumor growth (**K**), mRNA levels of typical IFN-Is (**L**) of HK1 tumors implanted into HuHSC-NCG mice (*n* = 5 in each group). (**M** and **N**) Schematic illustration and tumor growth of nonirradiated abscopal tumors and irradiated primary tumors with the indicated treatment (*n* = 6 in each group). Data are shown as the mean ± SD (**B**, **F**–**I**, **J**, and **L**) and the mean ± SEM (**D**, **E**, **K**, and **N**). *P < 0.05, **P < 0.01, ***P < 0.001, and ****P < 0.0001, by unpaired, 2-tailed Student’s *t* test (**B**, **J**, and **L**), 2-way ANOVA (**D**, **E**, **K**, and **N**), and 1-way ANOVA (**F**–**I**).

We subsequently sought to clarify whether inhibition of HO-1 improved the efficacy of RT in vivo. For a long time, HO-1 inhibitor screening was based on the extent of enzyme inhibition. However, here, we revealed that HO-1 exerted its effects via a nonenzymatic mode, and thus we first screened existing HO-1 inhibitors. Imidazole HO-1 inhibitors [including Zn-(II)-protoporphyrin IX and Tin-protoporphyrin IX] increased HO-1 expression and decreased IFN-β production under RT (Supplemental Figure 8, A and B), whereas a recently discovered inhibitor HO-1–IN-1 had an inhibitory effect on both in vitro and in vivo HO-1 expression and promoted IFN-β production (Supplemental Figure 8, A–C). More important, HO-1–IN-1 disrupted the endogenous interaction between cGAS and cleaved HO-1 as well as STING and full-length HO-1, leading to enhanced nuclear export, cGAMP production, and STING activation (Figure 9, A–C, and Supplemental Figure 8, D and E).

Next, we investigated the effect of HO-1–IN-1 in vivo, with or without regional RT, and found that HO-1–IN-1 combined with RT suppressed tumor growth compared with RT alone (Figure 9, D and E, and Supplemental Figure 8F). Further assays revealed that intratumoral IFN-I production, ISG levels (*H2kb* and *Cxcl10*), CD8^+^ T cell infiltration, and function (TNF-α and IFN-γ secretion) were elevated in the presence of HO-1–IN-1 (Figure 9, F–J, and Supplemental Figure 8, G–I). Consistently, in humanized mice, HO-1–IN-1 also further decreased the tumor volumes formed by HK1 cells and promoted intratumoral IFN-I production (Figure 9, K and L). Except for local control of irradiated sites, we also examined the effects of HO-1 inhibition on abscopal tumors (Figure 9M). RT combined with HO-1–IN-1 not only delayed substantial progression of the primary tumor, but also improved tumor control at the abscopal sites (Figure 9N).

To fully clarify the involvement of cGAS and STING, we generated *cGas*- or *Sting*-KO MC38 cells (Supplemental Figure 8J). We found that knocking out either *cGas* or *Sting* weakened the sensitizing effect of HO-1–IN-1 on RT, but still showed some effect (Supplemental Figure 8, K and L). Moreover, in a parallel experiment, anti-CD8–depleting antibody almost abolished the RT-sensitizing effect induced by HO-1 inhibition (Supplemental Figure 8M).

Taken together, these data demonstrate that inhibition of HO-1 has promising RT-sensitizing effects in multiple preclinical in vivo models.

### High expression of HO-1 correlates with unfavorable RT prognosis.

**Figure 10:**
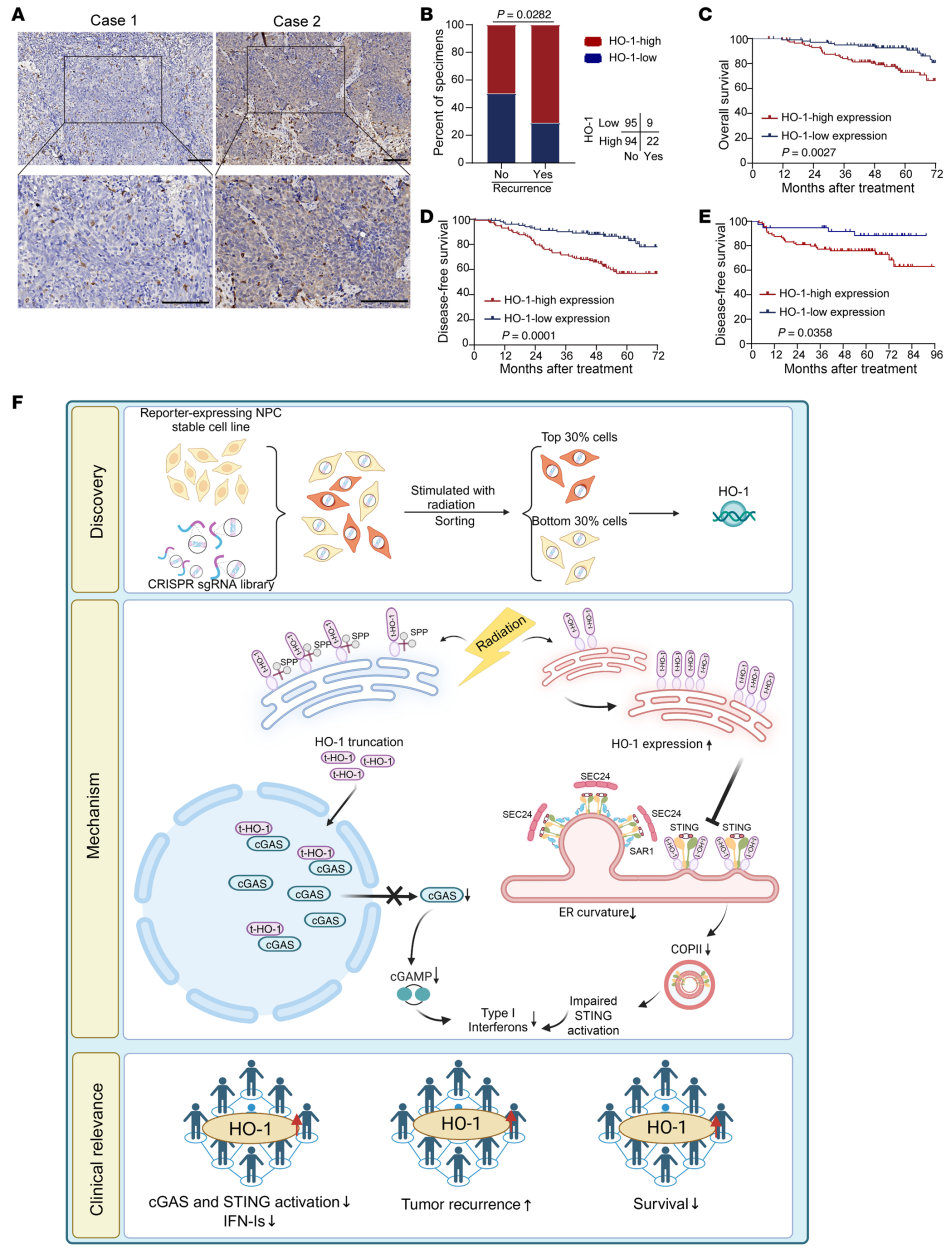
High expression of HO-1 correlates with unfavorable RT prognosis. (**A**) Representative images of immunohistochemical staining for HO-1 protein expression, which is graded according to the staining intensity in 220 NPC tissues. Scale bars: 100 μm. (**B**) Correlations of the locoregional recurrence status with HO-1 expression detected by IHC. *P* value was determined by 2-tailed χ^2^ test. (**C** and **D**) Kaplan-Meier analysis of OS (**C**) and DFS (**D**) according to HO-1 expression. (**E**) Kaplan-Meier analysis of DFS based on HO-1 expression in the published bulk RNA-Seq dataset. (**C**–**E**) *P* values were determined using the log-rank test. (**F**) Proposed working model of HO-1. By an unbiased CRISPR screen, we identified HO-1 as an irradiation-related regulator of IFN-I production. Mechanistically, irradiation induced HO-1 expression and promoted its cleavage. Cleaved HO-1 underwent nuclear translocation, interacted with cGAS, inhibited its nuclear export upon radiation, and suppressed its enzymatic activity. ER-anchored full-length HO-1 disturbed STING polymerization and subsequent COPII-mediated ER-Golgi transportation, leading to impaired activation of downstream signaling.

To determine the clinical significance of HO-1 expression in RT, we performed IHC staining of NPC tissues from 220 patients who had undergone RT. On the basis of the staining intensity, patients’ samples were categorized into a high HO-1 expression group (*n* = 116, 52.7%) and a low HO-1 expression group (*n* = 104, 47.3%) for further analysis (Figure 10A and Supplemental Table 4). By integrating the IHC results with our clinical data from Sun Yat-sen University Cancer Center (SYSUCC), we found that locoregional recurrence was positively correlated with high HO-1 expression in NPC (Figure 10B). Kaplan-Meier analysis revealed that high HO-1 expression was correlated with worse overall survival (OS) and disease-free survival (DFS) (Figure 10, C and D, and Supplemental Table 4). Consistently, HO-1 was associated with poor DFS in patients with NPC based on a published RNA-Seq dataset (43) (Figure 10E). Moreover, we also conducted survival analysis of patients from TCGA and Cbioportal. After stratifying patients according to those who received RT and those who did not, we found that for patients with esophageal carcinoma (ESCA) or glioblastoma (GBM), HO-1 expression did not correlate with progression-free survival (PFS) in patients who did not undergo RT, whereas its expression showed a strong correlation with worse PFS in patients who underwent RT (Supplemental Figure 9, A–D). Similarly, higher HO-1 expression was associated with worse OS in pediatric patients with brain cancer who received RT, but not in the non-RT cohort (Supplemental Figure 9, E and F). For patients with diffuse glioma, HO-1 expression was more deterministic of poor OS for patients who received RT compared with those who did not, as evidenced by higher HRs (Supplemental Figure 9, G and H).

In summary, these results suggest that high HO-1 expression is associated with an unfavorable prognosis and response to RT.

## Discussion

In this study, we performed a metabolism-based CRISPR/Cas9 screen to identify regulators of IFN-I production in the scenario of RT. On the basis of our data, HO-1 proved to be the key molecule inhibiting RT-induced IFN-Is through comprehensive suppression of the cGAS/STING pathway (Figure 10F). Canonically, HO-1 serves as a detoxifying enzyme for heme degradation, yet it impairs the activity of cGAS and STING independently of enzymatic activity. Here, HO-1 exerted this effect in a very direct manner by interacting with cGAS and STING, leading to their abnormal distribution and dysfunction. Moreover, we also revealed the close relationship between HO-1 and RT, which not only altered the form of HO-1 but also enhanced its expression. This indicates that, while RT induces the release of DAMPs to activate the inflammatory response, an inherent balance mechanism also exists. Therefore, HO-1 functions as the tip of the balance that can be adjusted to steer the reprogramming of the post-RT immune system.

The remodeling of the tumor immune microenvironment by RT depends on adjusting the balance between its intrinsic positive and negative regulatory factors (44). Effective arousal of antitumor immunity is important for long-term tumor control by RT. Insufficient IFN signaling is beneficial for tumor progression. The cGAS/STING pathway plays an important role in RT by recognizing large amounts of dsDNA and producing IFN-Is. Nevertheless, several mechanisms are implemented by tumor cells to impair the activation and transduction of signaling at different levels, including DNA repair enhancement (13, 45), and the regulation of the stability and activation of key components in the cGAS/STING pathway (46, 47). In comparison with other identified negative regulators of cGAS/STING signaling, HO-1, as revealed in this study, exhibits 2 notable features: an extremely strong association with RT and simultaneous, direct inhibitory effects on both cGAS and STING. HO-1 was originally characterized as an ER-located protein mediating heme degradation, yet increasing evidence has shown that HO-1 can be localized in other subcellular compartments besides ER (48). Hypoxia or incubation with hemin and H/HPX can induce the cleavage of HO-1 and its translocation to the nucleus (48, 49). Here, we found that RT resulted in HO-1 cleavage and nuclear translocation and that this process was mediated by SPP. Consistently, an earlier study showed that ER-anchored HO-1 was susceptible to cleavage in the intramembrane domain by SPP (32). Biologically, nuclear localization of HO-1 has been reported to be involved in mediating resistance to chemotherapy or promoting metastasis (48). Clinically, increased nuclear HO-1 expression also correlates with a higher histological grade and poorer outcomes for various types of cancer (50, 51). Our findings have contributed to an enriched understanding of the mechanism by which HO-1 promotes tumor progression, particularly in the context of limiting IFN-dependent immune surveillance. Moreover, under conditions of viral infection, such as HSV-1 or VSV, we also found that knocking out *HMOX1* in macrophages increased IFN-I production. However, another study showed that HO-1 is essential for IRF3-dependent transcription of downstream IFN-Is (52). Specifically, HO-1–deficient macrophages produced less IFN-Is in response to *Listeria monocytogenes* or Sendai virus infections, and the host became less resistant to these 2 infections. This contradiction may have arisen from the presence of distinct modification profiles of HO-1 and IRF3 under different conditions, leading to differences in the affinity for protein binding. In addition to PAMPs that activate pattern recognition receptors of the innate immune system, foreign microorganisms also produce specific virulence proteins that induce various biological changes in host cells, including distinct modification profiles. These may consequently lead to seemingly contradictory effects of HO-1. The above facts indicate that the nonenzymatic biological effects of HO-1 are of great significance and must be considered in future screening for HO-1 inhibitors. In addition to the unrecognized effects of HO-1, we also identified a role for IFN signaling in regulating its expression. The hypoxia- or oxidative stress–associated transcription factor NRF2 is thought to be the most relevant regulator of HO-1 expression (53). Here, we found that STAT1 also exerted a regulatory effect on HO-1, and from a macroscopic point of view, HO-1 formed a negative feedback loop with the cGAS/STING/IFN signaling pathway. It is worth mentioning that, due to the limitations of clinical practice, although we revealed an upregulatory effect of RT on HO-1, the effect of this part of upregulated HO-1 on patient outcomes could not be well represented in the existing clinical samples, illustrating the need for further development of noninvasive, nontoxic, real-time displayable chemical biology–based methods for HO-1 detection.

cGAS was first proposed as a DNA sensor in the cytosol, where it catalyzes the synthesis of a cyclic dinucleotide cGAMP. Nevertheless, recent studies revealed that cGAS also localizes in the nucleus of cells from different lineages. Nucleus-tethered cGAS is either inactive or has very low enzymatic activity but is endowed with the function to inhibit cellular homologous recombination repair or has other unidentified effects. In other words, the variations in distribution determine the different identities of cGAS (31). The classical nucleus-cytosol shuttle mechanism plays an important role in the localization of cGAS, yet the detailed regulatory mechanisms remain unknown. Importantly, multiple stimuli also affect the distribution of cGAS. Specifically, cGAS translocates to the nucleus in response to DNA damage caused by etoposide (13), but direct transfection of IFN stimulatory DNA (ISD) results in cGAS entry into the cytoplasm (38). It is interesting to note that, in contrast to etoposide, we observed that RT, which also induced substantial DNA damage, prompted cGAS to exit the nucleus. These results further highlight the complexity of regulating the nucleus-cytosol distribution of cGAS and partially illustrate the reason why the levels of induced IFN-I production vary under different treatments that cause DNA breaks. Theoretically, cellular transcription of IFN-Is via cGAS-STING requires 2 basic conditions: first, cGAS/STING can be activated, and second, the intracellular transcription system remains intact (54). When cGAS is confined in the nucleus, the above 2 conditions are much less likely to be reached. This also suggests that intervening in the distribution of cGAS, as required in various treatments or at different stages of the same treatment, may represent a potential strategy for achieving improved therapeutic outcomes. HO-1, as we discovered in our study, is the highly selective molecule to achieve this and deserves to be further explored for the possibility of combination therapy.

STING oligomerization, the core change in the process of STING activation, is critical for the translocation of STING from the ER to the Golgi and the subsequent formation of a platform for activating downstream signaling such as TBK1 and IRF3 (18, 20, 21). The endogenous agonistic ligand cGAMP, exogenous small-molecule agonists (diABZI, ref. 55; DMXAA, ref. 56, etc.), and a variety of autoimmune disease–associated, self-activating mutations (57) all exert their biologic effects through induction or stabilization of STING oligomerization. Here, we found that HO-1 inhibited STING oligomerization by occupying the homodimer interface in STING tetramers and destabilizing the formed STING tetramers. In the current studies, the regulation of STING oligomerization primarily focused on the relevant mechanisms mediated by modification of the amino acid residues on STING (19), but the inhibitory mechanisms mediated directly by collision or spatial site blocking have not been elucidated. Furthermore, it also suggests that certain STING-binding proteins located on the ER may have similar potentials that warrant further investigation.

To conclude, our data suggest that HO-1, when acted upon with RT, exerts a comprehensive inhibitory effect on cGAS and STING activity, making it a highly promising target that deserves ongoing investigation as a potential combination therapy.

## Methods

### Sex as a biological variable.

Sex was not considered as a biological variable; therefore, human and mouse studies included both sexes. In the case of the 4T1 murine breast cancer model, only female mice were utilized, as breast cancer predominantly affects female patients.

### Cell culture and treatment.

Cells from the human NPC cell line (HK1, a gift from M.S. Zeng, Sun Yat-sen University Cancer Center, Guangzhou, China), the human prostate cancer cell line (DU145), the human fibrosarcoma cell line (HT-1080), the human cervical cancer cell line (HeLa), the human breast epithelial cell line (MDM-MB-231), the human embryonic kidney 293T cell line (HEK293T), the mouse colon adenocarcinoma cell line (MC38), the mouse melanoma cell line (B16), and the mouse breast cancer cell line (4T1) were cultured in RPMI 1640/DMEM media supplemented with 10% FBS. Cells from the normal human nasopharyngeal epithelial cell line NP69 were cultured in keratinocyte serum–free medium (Invitrogen, Thermo Fisher Scientific) supplemented with bovine pituitary extract (BD Biosciences). Cells from the normal human breast epithelial line MCF10A were cultured in specific epithelial culture medium (Procell). Cells were irradiated at a dose of 10 Gy by RS-2000-PRO-225 Biological Irradiator. We chose 10 Gy in our study on the basis of the results that a RT dose of 10 Gy had the most potent ability to induce IFN-I production in both HK1 and HeLa cell lines (data not shown). The sources of all cell lines are listed in Supplemental Table 5.

### siRNAs, plasmids, and lentivirus.

siRNAs targeting the indicated genes were designed and synthesized by GenePharma. Efficiency was determined by reverse transcription quantitative PCR (RT-qPCR) or Western blotting after 24–48 hours of transfection. cDNA sequences were subcloned into the pSin-EF2-puro vector for exogenous expression. Lentivirus was produced by cotransfection of lentivirus vectors with pMD2.G and psPAX2 plasmids into HEK293T cells. For infection, cells were cocultured with lentivirus-containing supernatants for 6 hours and then washed and cultured in fresh medium.

### Reagents and ELISA.

An ELISA kit was used to detect the concentrations of human IFN-β (Neobioscience) and cGAMP (Cayman) according to the manufacturer’s instructions. Detailed information and other reagents used in the this study are listed in Supplemental Table 5.

### Generation of gene-deficient cells.

KO cells were generated using the CRISPR/Cas9 system. Cells were transfected with the pX458 expressing SpCas9, GFP, and sgRNA targeting the indicated genes. Positive cells were sorted on the basis of GFP expression with a flow cytometer. sgRNA sequences were designed with the Benchling online tool (https://benchling.com) and are listed in Supplemental Table 6.

### mCherry reporter.

The plasmid encoding the mCherry reporter gene, which was driven by ISREs and the IFN-β promoter, was subcloned into a dual-promoter lentiviral plasmid. This construct was infected into HK1 cells to generate stably expressing transgenic cell lines.

### CRISPR/Cas9 screen.

The Human CRISPR Metabolic Gene Knockout library (Addgene, 110066) contains 29,790 unique sgRNAs targeting 2,981 genes (25). HK1 cells were transduced at a MOI of approximately 0.3 in sufficient numbers for 500× coverage of the gRNA library. Cells were then cultured with puromycin for 5 days and without puromycin for 3 days. After amplification,a sufficient number of cells was maintained for 500× coverage of the gRNA library for subsequent steps. Positive HK1 cells were irradiated at a dose of 10 Gy. After radiation, cells were collected and sorted by the flow cytometer according to mCherry-expressing cells, which were sorted into 2 cell populations: the highest 30% of mCherry-expressing cells and the lowest 30%. Genomic DNA from these 2 cell populations was extracted for PCR amplification. The sgRNA library amplicons were then separated in agarose gel. After purification, the products were subjected to next-generation sequencing.

### Data analysis of the pooled CRISPR screen.

Screening hits identification and quality control were performed using the MAGeCKFlute program. The MAGeCk count module was used to generate the raw count table containing the raw sequencing read counts of each sgRNA for each sample. The MAGeCK RRA module was used to output a score for both negative selection and positive selection. MAGeCK RRA also output a *P* value or FDR for the scores of each gene. The scatterplots and volcano plots were generated on the basis of the results obtained from MAGeCK RRA using R scripts.

### Isolation of BMDMs and virus infection.

Isolation of BMDMs was performed as described previously (58). HSV-1 and VSV were propagated and titered on Vero cells. Virus titers were measured by means of 50% of the tissue culture’s infectious dose (TCID_50_). Cells were stimulated with HSV-1 (MOI of 1) or VSV (MOI of 1). HSV-1 and VSV were gifts from J. Cui (School of Life Sciences, Sun Yat-sen University, Guangzhou, China).

### RNA extraction and RT–qPCR.

Total RNA was extracted from cells using TRIzol (Invitrogen, Thermo Fisher Scientific) and then transcribed to cDNA using a reverse transcription kit (Promega). RT-qPCR was conducted with SYBR Green PCR Master Mix (Vazyme). The primer sequences are listed in Supplemental Table 6.

### Dual luciferase assay.

HEK293T cells were transfected with the IFN-β–luc reporter, *Renilla*-Luc plasmids, and other indicated plasmids, followed by use of a dual-luciferase assay kit (Promega) according to the manufacturer’s instructions. Luciferase activity was normalized to *Renilla* luciferase activity for each sample.

### Immunoblotting and coimmunoprecipitation.

Cells were lysed and sonicated to obtain whole-cell extracts. Total protein was separated by SDS-PAGE and transferred onto nitrocellulose or PVDF membranes, followed by visualization (Tanon). For coimmunoprecipitation, whole-cell lysates were immunoprecipitated overnight at 4°C with the indicated antibodies, followed by incubation with Pierce Protein A/G Magnetic Beads (Thermo Fisher Scientific), or directly incubated with Magnetic Beads (Thermo Fisher Scientific) conjugated with labeled antibodies. Detailed information is provided in Supplemental Table 7.

### Confocal microscopy.

Cells were seeded on coverslips, fixed with 4% formaldehyde, and permeabilized by 0.1%–0.2% Triton X-100 after transfection or stimulations as indicated. After blocking, cells were incubated with primary and secondary antibodies (antibodies are listed in Supplemental Table 7). Images were acquired with a laser-scanning confocal microscope (Zeiss LSM880 or Olympus FV1000).

### Cytoplasmic and nuclear fractionation.

Cytoplasmic and nuclear protein fractions were extracted using NE-PER Nuclear and Cytoplasmic Extraction Reagents (Thermo Fisher Scientific) according to the protocol provided by the manufacturer. The proteins were separated by SDS-PAGE for further detection.

### Animal experiments.

Tumor volumes were recorded (tumor volume = length × width^2^/2). The following reagents were used: heme oxygenase-1-IN-1 (MedChemExpress, i.p. 25 mg/kg) and InVivoMab anti–mouse CD8a (Bio X Cell, i.p., 200 μg/test). In the murine model of RT, we referred to previous published studies, which used 15 Gy for the MC38 model (59), 21 Gy for the B16 model (60), and 3 × 8 Gy for the 4T1 model (61), and these doses could effectively control tumor growth or elicited an abscopal effect of RT or antitumor immunity. As for the generation of NPC xenografts, a 6-week-old female, specific pathogen–free (SPF) humanized NCG mouse model was established by tail vein injection of human PBMCs (5 × 10^6^) and validated by the detection of more than 1% human CD45^+^ cells in the peripheral blood of the mice 1 week after injection. Mice were then s.c. inoculated with 1 × 10^6^ HK1 cells. LyzCre/Cre mice were gifts from X.J. Xia (Sun Yat-sen University Cancer Center, Guangzhou, China).

### Flow cytometry.

Cells and tissues were harvested and digested into a single-cell suspension. To analyze surface markers, the cells were incubated with the indicated antibodies. For intracellular cytokine staining, cells were fixed, permeabilized, and stained with the indicated antibodies. The antibodies used are listed in Supplemental Table 7.

### Clinical specimens.

Six fresh-frozen NPC specimens and 220 paraffin-embedded NPC specimens were collected from the Sun Yat-sen University Cancer Center (Guangzhou, China). The NPC tissues for IHC staining were collected before RT to predict the efficacy of RT. Patients underwent RT with a median total dose of 70 Gy (IQR, 68–70 Gy) and 2.0–2.3 Gy per fraction (median fraction, 2.0 Gy), at 5 fractions per week. The clinical features of these patients are shown in Supplemental Table 4.

### IHC.

Paraffin-embedded tissue sections were deparaffinized and rehydrated, antigens were retrieved, and endogenous peroxidase inactivated. After blocking, the slides were incubated with primary antibody at 4°C overnight. Subsequently, they were labeled with an HRP-conjugated secondary antibody, followed by diaminobenzidine development. All sections were scored by 2 experienced pathologists according to the immunoreactive score (IRS) system. The intensity of staining was scored as follows: 0 (negative staining), 1 (weak staining), 2 (moderate staining), and 3 (strong staining). The percentage of positive tumor cells was scored as follows: 1 (<10%), 2 (10%–35%), 3 (35%–70%), and 4 (>70%).

### Analysis based on public datasets.

TCGA datasets were obtained from UCSC Xena database (https://xena.ucsc.edu/). The pediatric brain cancer or diffuse glioma dataset was obtained from Cbioportal (https://www.cbioportal.org/). To evaluate the efficacy of NPC RT, bulk RNA-Seq data on 128 patients with NPC were analyzed.

### Molecular docking.

The structure of the STING dimer and tetramer was constructed by Alphafold21 and parametrized by Amber-fb15 force field2 in tleap in Amber20 package3. The complexes of the STING dimer and HO-1 dimer as well as the STING tetramer and HO-1 dimer were built by HDOCK webserver4 and evaluated in MD simulations, which were done by pmemd.cuda in Amber20 package3. The MD simulations were initialized by a multistep optimization, which first optimized the solvents and then optimizes the protein systems with the solvent. All the protein behaviors were described by Amber-fb15 force field2, and the MD simulations were done by pmemd.cuda in Amber 20.3.

### Statistical analysis and reproducibility.

Statistical analysis was performed with GraphPad Prism 9.0 (GraphPad Software), SPSS 24.0 software, or R software. An unpaired, 2-tailed Student’s *t* test or χ^2^ test was used for statistical analysis between 2 groups, and 1-way ANOVA was performed for more than 2 groups. The survival curves were constructed with Kaplan–Meier method and analyzed by log-rank test (62). Data are presented as the mean ± SEM or SD for a minimum of 3 independent experiments. A *P* value of less than 0.05 was considered statistically significant. Three biologically independent experiments were performed unless otherwise stated.

### Study approval.

Our study was approved by the institutional ethics review boards of Sun Yat-sen University Cancer Center (SL-B2022-621-01), and all patients provided written informed consent to participate in the study. the experimental animal ethics committee of Sun Yat-sen University approved all animal experiments (SYSU-IACUC-2023-000302, SYSU-IACUC-2023-001177, and SYSU-IACUC-2024-001780).

### Data availability.

Values for all data points in graphs are reported in the Supporting Data Values file. Bulk RNA-Seq and CRISPR Screen data (PRJCA028593) have been deposited in the National Genomics Data Center (NGDC) (https://ngdc.cncb.ac.cn/). Further requests for data should be directed to the corresponding authors.

## Author contributions

The order of the co–first authors was determined on the basis of the significance, time, and effort each author invested in the project. XYL, CX, JM, and CQZ conceived the project. CQZ, ZJD, JWW, CD, ZL, ZMX, and LFL performed the experiments. CQZ, ZJD, and JWW developed methodology. CQZ, ZJD, WPC, and KBY performed data analysis. CQZ, HMW, and TXH contributed to clinical simple collection and analysis. XYL and CQZ wrote the manuscript.

## Supplementary Material

Supplemental data

Unedited blot and gel images

Supporting data values

## Version 1 12/02/2024

Electronic publication

## References

[1] Schaue D, McBride WH (2015). Opportunities and challenges of radiotherapy for treating cancer. Nat Rev Clin Oncol.

[2] Deng L (2014). STING-dependent cytosolic DNA sensing promotes radiation-induced Type I interferon-dependent antitumor immunity in immunogenic tumors. Immunity.

[3] Schaue D, McBride WH (2015). Opportunities and challenges of radiotherapy for treating cancer. Nat Rev Clin Oncol.

[4] Kachikwu EL (2011). Radiation enhances regulatory T cell representation. Int J Radiat Oncol Biol Phys.

[5] Xu J (2013). CSF1R signaling blockade stanches tumor-infiltrating myeloid cells and improves the efficacy of radiotherapy in prostate cancer. Cancer Res.

[6] Burnette BC (2011). The efficacy of radiotherapy relies upon induction of type i interferon-dependent innate and adaptive immunity. Cancer Res.

[7] Zitvogel L (2015). Type I interferons in anticancer immunity. Nat Rev Immunol.

[8] Hou Y (2018). Non-canonical NF-κB antagonizes STING sensor-mediated DNA sensing in radiotherapy. Immunity.

[9] Yamazaki T (2020). Mitochondrial DNA drives abscopal responses to radiation that are inhibited by autophagy. Nat Immunol.

[10] Ma D (2021). Arginine methyltransferase PRMT5 negatively regulates cGAS-mediated antiviral immune response. Sci Adv.

[11] Kuchitsu Y (2023). STING signalling is terminated through ESCRT-dependent microautophagy of vesicles originating from recycling endosomes. Nat Cell Biol.

[12] Srikanth S (2019). The Ca(2+) sensor STIM1 regulates the type I interferon response by retaining the signaling adaptor STING at the endoplasmic reticulum. Nat Immunol.

[13] Liu H (2018). Nuclear cGAS suppresses DNA repair and promotes tumorigenesis. Nature.

[14] Bai J, Liu F (2022). Nuclear cGAS: sequestration and beyond. Protein Cell.

[15] Guey B (2020). BAF restricts cGAS on nuclear DNA to prevent innate immune activation. Science.

[16] Barber GN (2015). STING: infection, inflammation and cancer. Nat Rev Immunol.

[17] Liu S (2023). The mechanism of STING autoinhibition and activation. Mol Cell.

[18] Saitoh T (2009). Atg9a controls dsDNA-driven dynamic translocation of STING and the innate immune response. Proc Natl Acad Sci U S A.

[19] Fang R (2021). Golgi apparatus-synthesized sulfated glycosaminoglycans mediate polymerization and activation of the cGAMP sensor STING. Immunity.

[20] Ergun SL (2019). STING polymer structure reveals mechanisms for activation, hyperactivation, and inhibition. Cell.

[21] Zhang BC (2020). STEEP mediates STING ER exit and activation of signaling. Nat Immunol.

[22] Ayer A (2016). Heme oxygenases in cardiovascular health and disease. Physiol Rev.

[23] Zhang L (2023). STING is a cell-intrinsic metabolic checkpoint restricting aerobic glycolysis by targeting HK2. Nat Cell Biol.

[24] Fang R (2023). ARMH3-mediated recruitment of PI4KB directs Golgi-to-endosome trafficking and activation of the antiviral effector STING. Immunity.

[25] Birsoy K (2015). An essential role of the mitochondrial electron transport chain in cell proliferation is to enable aspartate synthesis. Cell.

[26] Hu H (2014). Changes in glucose-6-phosphate dehydrogenase expression results in altered behavior of HBV-associated liver cancer cells. Am J Physiol Gastrointest Liver Physiol.

[27] Makokha GN (2023). Deficiency of SCAP inhibits HBV pathogenesis via activation of the interferon signaling pathway. Virology.

[28] Wu SY (2021). MYC suppresses STING-dependent innate immunity by transcriptionally upregulating DNMT1 in triple-negative breast cancer. J Immunother Cancer.

[29] Diao F (2007). Negative regulation of MDA5- but not RIG-I-mediated innate antiviral signaling by the dihydroxyacetone kinase. Proc Natl Acad Sci U S A.

[30] Liu S (2015). Phosphorylation of innate immune adaptor proteins MAVS, STING, and TRIF induces IRF3 activation. Science.

[31] Sun L (2013). Cyclic GMP-AMP synthase is a cytosolic DNA sensor that activates the type I interferon pathway. Science.

[32] Hsu FF (2015). Signal peptide peptidase-mediated nuclear localization of heme oxygenase-1 promotes cancer cell proliferation and invasion independent of its enzymatic activity. Oncogene.

[33] Valerie K (2007). Radiation-induced cell signaling: inside-out and outside-in. Mol Cancer Ther.

[34] Hein AL (2014). Radiation-induced signaling pathways that promote cancer cell survival (review). Int J Oncol.

[35] Yücel SS (2019). The metastable XBP1u transmembrane domain defines determinants for intramembrane proteolysis by signal peptide peptidase. Cell Rep.

[36] Lemberg MK (2011). Intramembrane proteolysis in regulated protein trafficking. Traffic.

[37] Jiang H (2019). Chromatin-bound cGAS is an inhibitor of DNA repair and hence accelerates genome destabilization and cell death. EMBO J.

[38] Liu ZS (2019). G3BP1 promotes DNA binding and activation of cGAS. Nat Immunol.

[39] Sun H (2021). A nuclear export signal is required for cGAS to sense cytosolic DNA. Cell Rep.

[40] Gui X (2019). Autophagy induction via STING trafficking is a primordial function of the cGAS pathway. Nature.

[41] Doucet CM (2015). Membrane curvature sensing by amphipathic helices is modulated by the surrounding protein backbone. PLoS One.

[42] Shang G (2019). Cryo-EM structures of STING reveal its mechanism of activation by cyclic GMP-AMP. Nature.

[43] Chen YP (2020). Single-cell transcriptomics reveals regulators underlying immune cell diversity and immune subtypes associated with prognosis in nasopharyngeal carcinoma. Cell Res.

[44] Weichselbaum RR (2017). Radiotherapy and immunotherapy: a beneficial liaison?. Nat Rev Clin Oncol.

[45] Nakamura K (2021). Inhibition of DNA-PK with AZD7648 sensitizes tumor cells to radiotherapy and induces Type I IFN-dependent durable tumor control. Clin Cancer Res.

[46] Ma Z (2023). AhR diminishes the efficacy of chemotherapy via suppressing STING dependent type-I interferon in bladder cancer. Nat Commun.

[47] Sun H (2017). USP13 negatively regulates antiviral responses by deubiquitinating STING. Nat Commun.

[48] Mascaró M (2021). Nuclear localization of heme oxygenase-1 in pathophysiological conditions: does it explain the dual role in cancer?. Antioxidants (Basel).

[49] Hwang HW (2009). Oligomerization is crucial for the stability and function of heme oxygenase-1 in the endoplasmic reticulum. J Biol Chem.

[50] Wegiel B (2013). Carbon monoxide expedites metabolic exhaustion to inhibit tumor growth. Cancer Res.

[51] Gandini NA (2012). Nuclear localization of heme oxygenase-1 is associated with tumor progression of head and neck squamous cell carcinomas. Exp Mol Pathol.

[52] Tzima S (2009). Myeloid heme oxygenase-1 regulates innate immunity and autoimmunity by modulating IFN-beta production. J Exp Med.

[53] Ma Q (2013). Role of nrf2 in oxidative stress and toxicity. Annu Rev Pharmacol Toxicol.

[54] Hopfner KP, Hornung V (2020). Molecular mechanisms and cellular functions of cGAS-STING signalling. Nat Rev Mol Cell Biol.

[55] Ramanjulu JM (2018). Design of amidobenzimidazole STING receptor agonists with systemic activity. Nature.

[56] Ding C (2020). Small molecules targeting the innate immune cGAS-STING-TBK1 signaling pathway. Acta Pharm Sin B.

[57] Dobbs N (2015). STING activation by translocation from the ER is associated with infection and autoinflammatory disease. Cell Host Microbe.

[58] Chen M (2016). TRIM14 inhibits cGAS degradation mediated by selective autophagy receptor p62 to promote innate immune responses. Mol Cell.

[59] Li JY (2023). TRIM21 inhibits irradiation-induced mitochondrial DNA release and impairs antitumour immunity in nasopharyngeal carcinoma tumour models. Nat Commun.

[60] Uribe-Herranz M (2020). Gut microbiota modulate dendritic cell antigen presentation and radiotherapy-induced antitumor immune response. J Clin Invest.

[61] Vanpouille-Box C (2017). DNA exonuclease Trex1 regulates radiotherapy-induced tumour immunogenicity. Nat Commun.

[62] Qiao H (2022). Association of intratumoral microbiota with prognosis in patients with nasopharyngeal carcinoma from 2 hospitals in China. JAMA Oncol.

