# Supplementary material for: HO-1 impairs the efficacy of radiotherapy by redistributing cGAS and STING in tumors
Source: J Clin Invest. 2024 Dec 2;134(23):e181044. doi: 10.1172/JCI181044 (PMC11601901; doi:10.1172/JCI181044)
Supplement: Supplemental data [file jci-134-181044-s084.pdf]

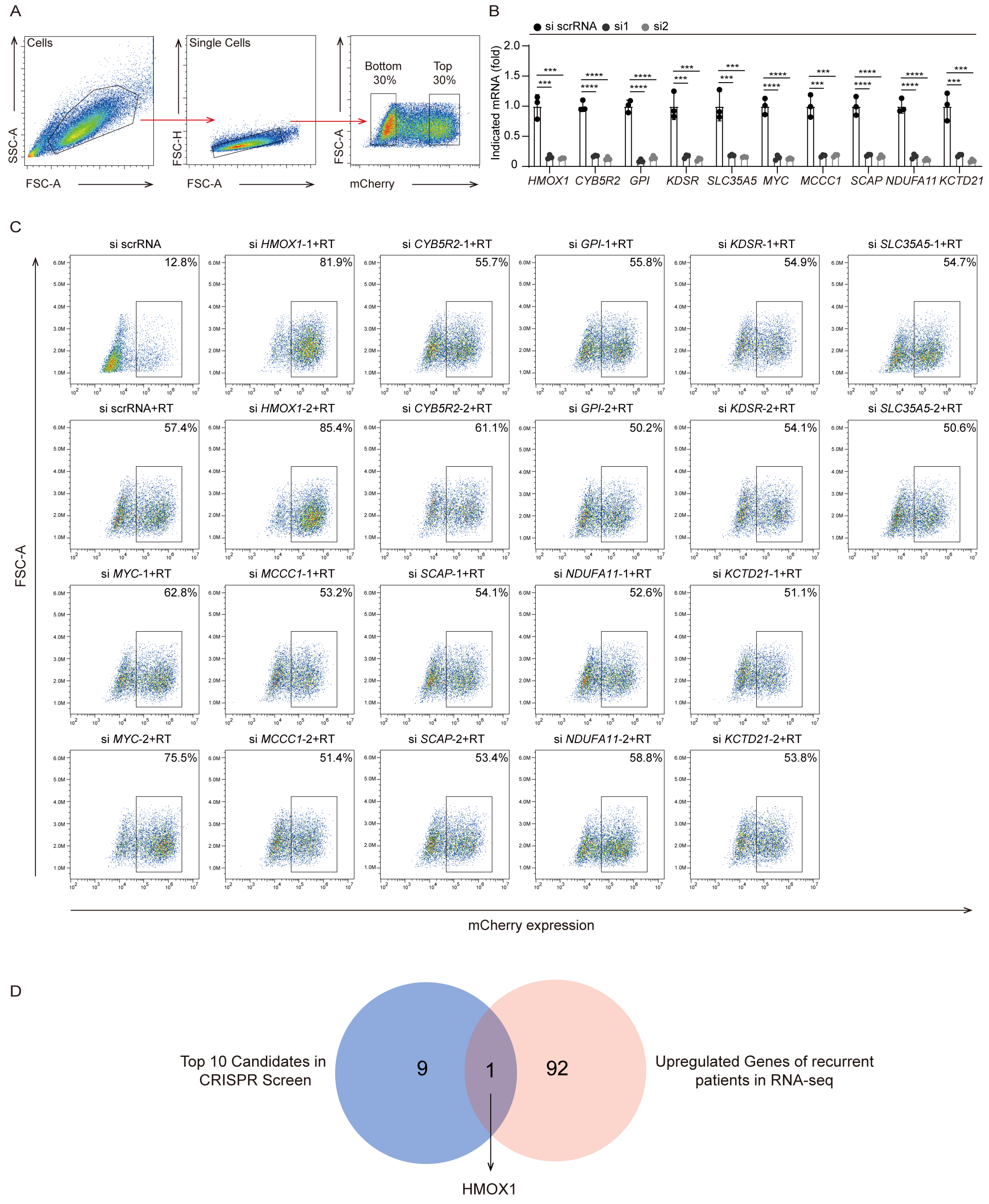

Supplemental Figure 1. A metabolic CRISPR/Cas9 screen identifies HMOX1 as a potent IFN-Is production inhibitor in response to radiotherapy.

(A) Representative gating strategy for flow cytometry-based sorting of the CRISPR screen of reporter-expressing cells stimulated with RT.

(B) mRNA expression for indicated genes knocking-down efficiency (related to 1F-H). *P*-value by One-way ANOVA. *P* -value < 0.05 as statistic difference. **P*<0.05; ***P*<0.01; ****P*<0.001; *****P*<0.0001. Data are shown as the mean ± SD (n=3 three biologically independent samples).

(C) Representative flow cytometry exhibition of reporter expression after interfering the top 10 candidates in CRISPR screen.

(D) NPC tissues from at Sun Yat-sen University Cancer Center (SYSUCC) with or without local recurrence after radiotherapy were collected for RNA sequencing analysis. The intersection between top 10 genes in CRISPR screen and 93 upregulated genes in the local recurrence group of RNA-seq.

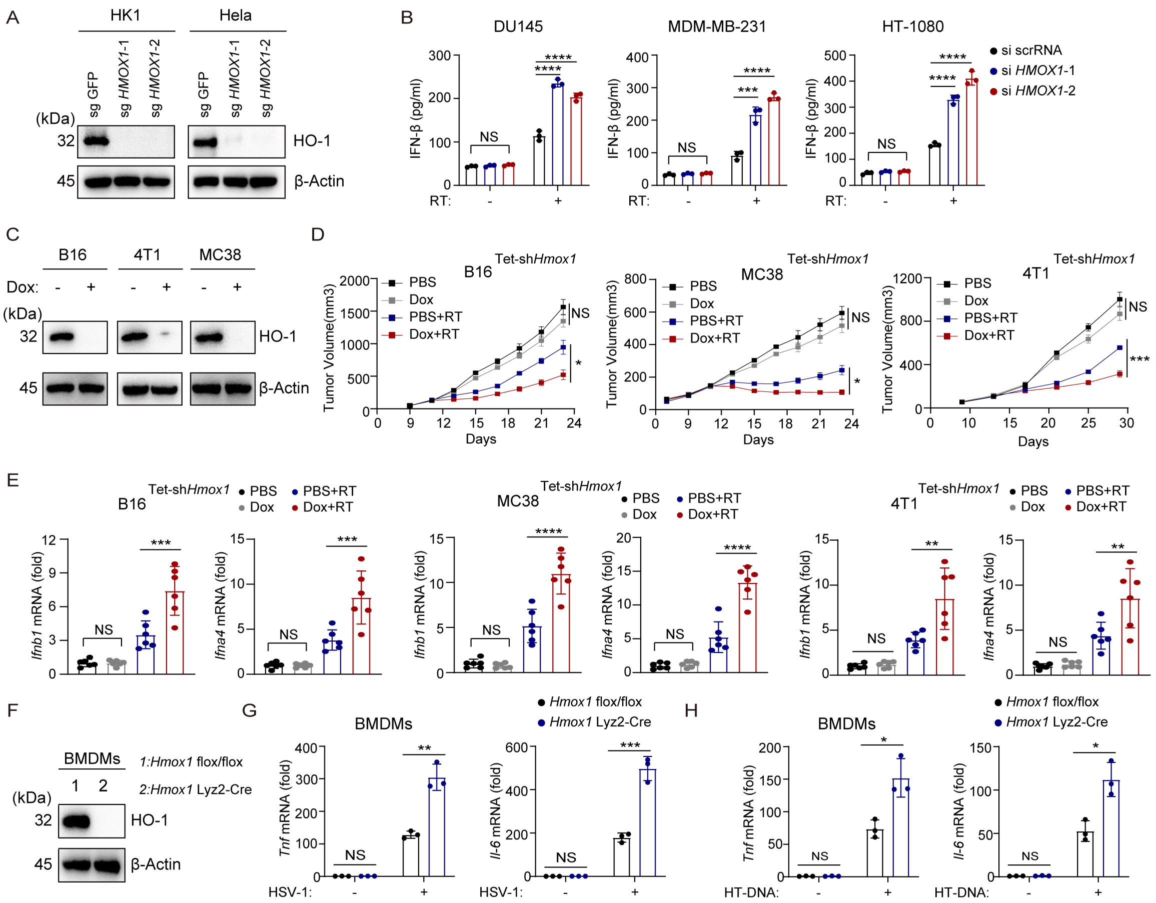

Supplemental Figure 2. HO-1 inhibits RT-mediated IFN-Is production.

(A) Immunoblot analysis of *HMOX1* knocking-out efficiency in the indicated cell lines.

(B) *HMOX1* was knocked down in the indicated cell lines, following by ELISA of IFN-β content in the supernatant before and after RT stimulation.

(C) Immunoblot analysis of doxycycline (Dox)-induced *Hmox1* knocking-down efficiency in indicated cells.

(D,E) The effect of knocking-down *Hmox1* combined with radiotherapy on tumor growth (D) or typical IFN-Is mRNA levels (E) of indicated tumors (n=6 in each group).

(F) Immunoblot analysis of *Hmox1* knocking-out efficiency in BMDMs (related to Figure 2G).

(G,H) *Tnf* and *Il6* mRNA levels of BMDMs from *Hmox1*$^{fl/fl}$ and *Hmox1*$^{fl/fl}$ Lyz$^{Cre/Cre}$ mice. BMDMs were infected with HSV-1 or HT-DNA.

(B,E) *P*-value by One-way ANOVA. Data are shown as the mean ± SD. (D) *P*-value by Two-way ANOVA. Data are shown as the mean ± SEM. (G,H) *P*-value by Student's t-test. Data are shown as the mean ± SD. All *P*-value< 0.05 as statistic difference. *P<0.05; **P<0.01; ***P<0.001; ****P<0.0001. n=3 three biologically independent experiments, unless otherwise indicated.

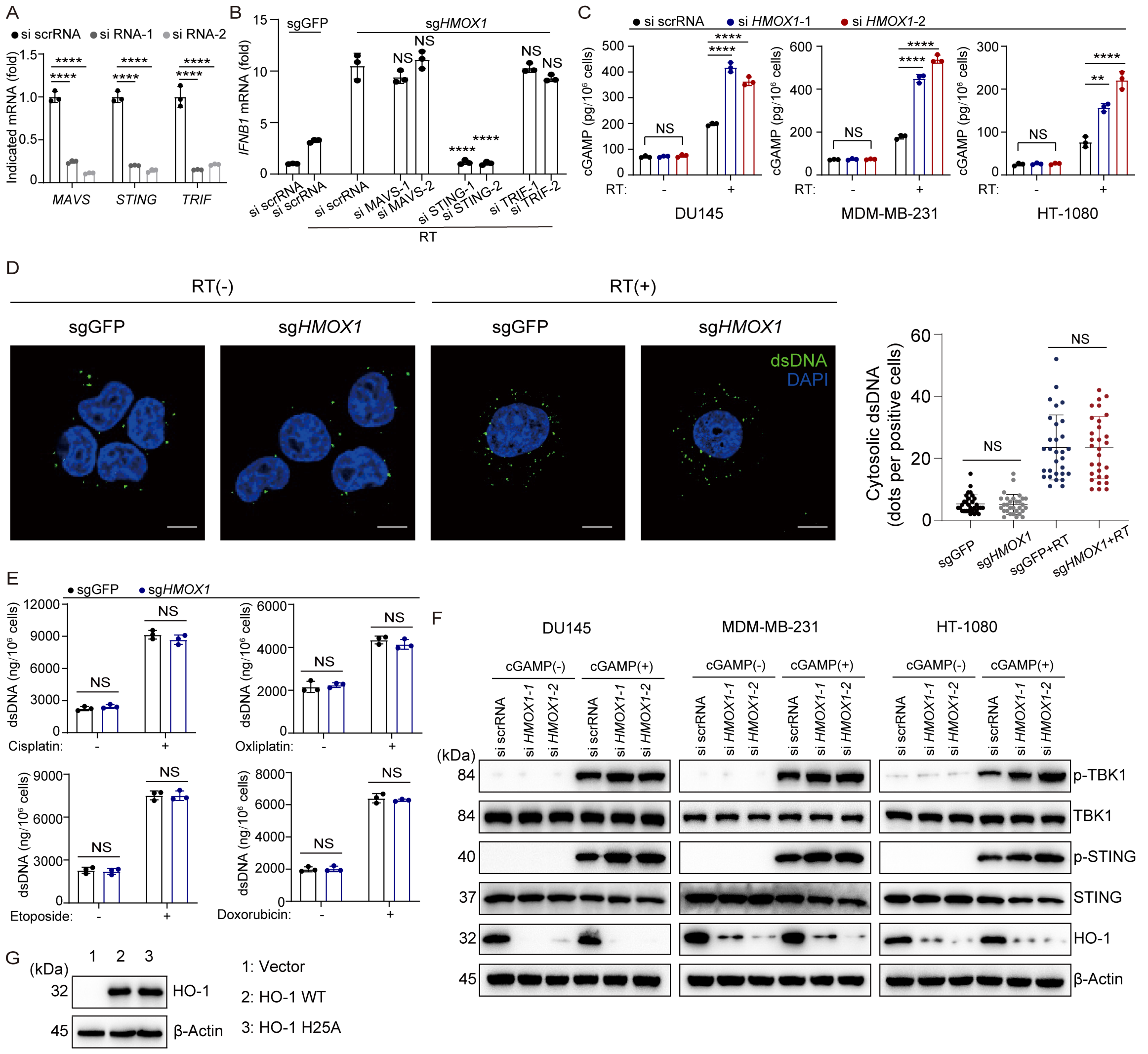

Supplemental Figure 3. HO-1 inhibits the activity of cGAS and STING under RT independent of its enzymatic activity.

(A) mRNA levels of indicated genes for knocking-down efficiency.

(B) *IFNB1* mRNA levels of knocking-down indicated genes with RT treatment. *P*-value was determined by comparing to sg*HMOX1*+si scrRNA+RT group.

(C) ELISA of cGAMP production of control or *HMOX1* knocked-down cells before and after RT stimulation in the indicated cell lines.

(D) Cytosolic DNA accumulation was assessed by immunofluorescence staining with dsDNA-specific antibody. Representative images (scale bar, 10 μm) and quantitative results are shown (n = 30 cells per group).

(E) Cytoplasmic dsDNA contents in HK1 cells stimulated with indicated chemotherapeutics.

(F) Immunoblot analysis of indicated proteins from control or *HMOX1* knocked-down cells with or without 2’3’-cGAMP stimulation.

(G) Immunoblot analysis for wildtype HO-1 or HO-1H25A expression in HK1 cells.

(A,B,C,D) *P*-value by One-way ANOVA. Data are shown as the mean ± SD. (E) *P*-value by Student's t-test. Data are shown as the mean ± SD. All *P*-value< 0.05 as statistic difference. **P*<0.05; ***P*<0.01; ****P*<0.001; *****P*<0.0001. n=3 three biologically independent experiments, unless otherwise indicated.

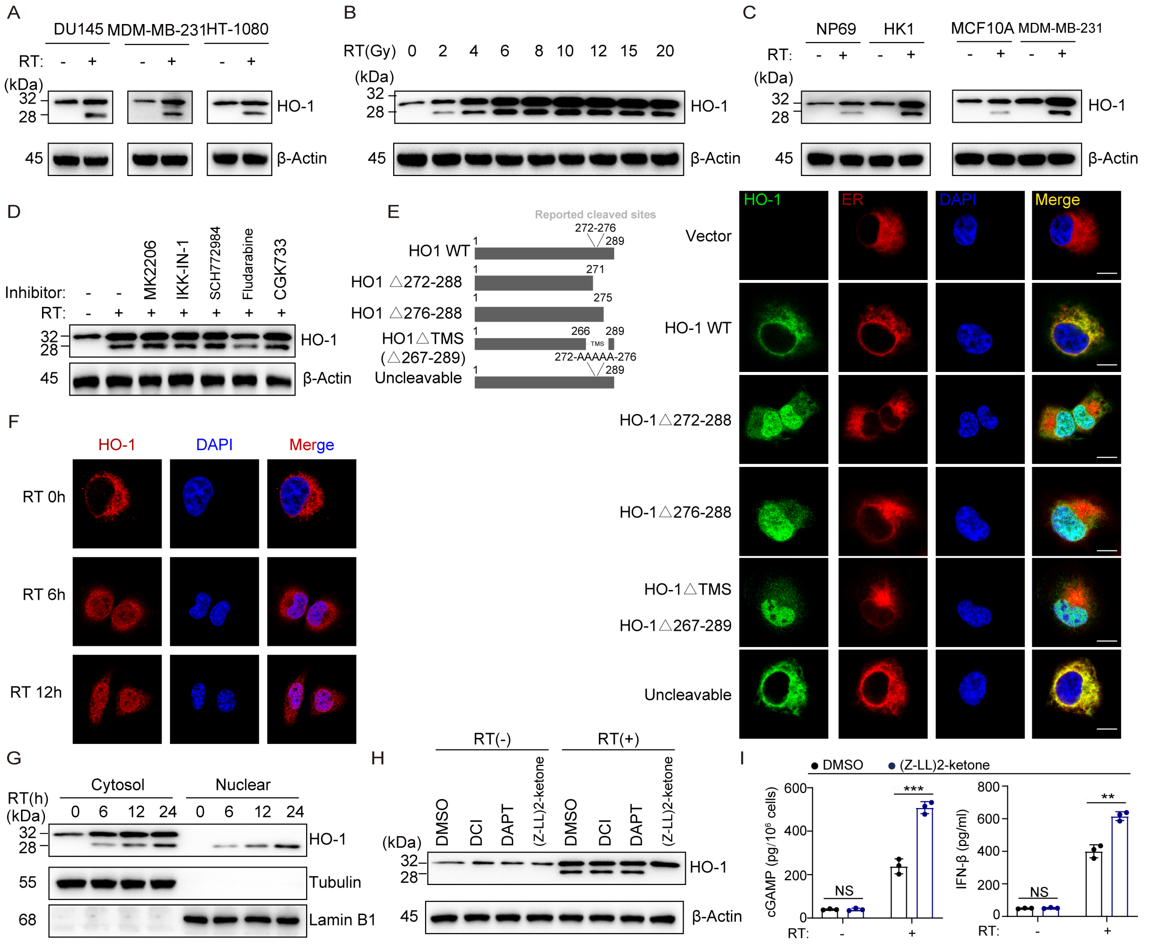

Supplemental Figure 4. RT induces HO-1 and promotes its cleavage.

(A) Immunoblot analysis of HO-1 expression and truncation in indicated cell lines before and after RT.

(B) Immunoblot analysis of HO-1 expression and truncation in HK1 cells stimulated with indicated RT doses.

(C) Immunoblot analysis of HO-1 expression and truncation in indicated normal and tumor cell lines before and after RT.

(D) HK1 cells were pretreated with indicated inhibitors (MK2206, an AKT inhibitor, targeting PI3K-AKT pathway; IKK-IN-1, an IKK inhibitor, targeting NF-κB pathway; SCH772984, an ERK inhibitor, targeting MAPK pathway; Fludarabine, an STAT1 inhibitor, targeting Jak-STAT1 pathway; CGK733, an ATR inhibitor). After RT, HO-1 expression was determined with immunoblot analysis.

(E) Subcellular distribution (ER and nucleus) of HO-1 or indicated mutants was determined with immunofluorescence staining in HK1 cells. Calreticulin staining for ER; DAPI staining for nucleus; scale bar, 10 μm.

(F) Subcellular distribution of endogenous HO-1 was determined with immunofluorescence staining in HeLa cells stimulated with RT (scale bar, 10 μm).

(G) Nuclear and cytoplasmic protein extraction experiment was performed to determine the cellular localization of endogenous HO-1 at indicated timepoint of RT in HeLa cells.

(H) HK1 cells were pretreated with indicated intramembrane protease inhibitors (DCI: an inhibitor of Rhomboid serine proteases; DAPT: an inhibitor of γ-secretase; (Z-LL)2-ketone: an inhibitor of SPP). Before and after RT, HO-1 expression and truncation was determined with immunoblot analysis.

(I) HK1 cells were pretreated with (Z-LL)2-ketone (an inhibitor of SPP). cGAMP (I) or IFN-β (J) production was determined with ELISA before and after RT. *P*-value by Student's t-test. *P* -value < 0.05 as statistic difference. Data are shown as the mean ± SD. **P*<0.05; ***P*<0.01; ****P*<0.001; *****P*<0.0001.

All representative data from one experiment are shown (n = 3 biologically independent experiments).

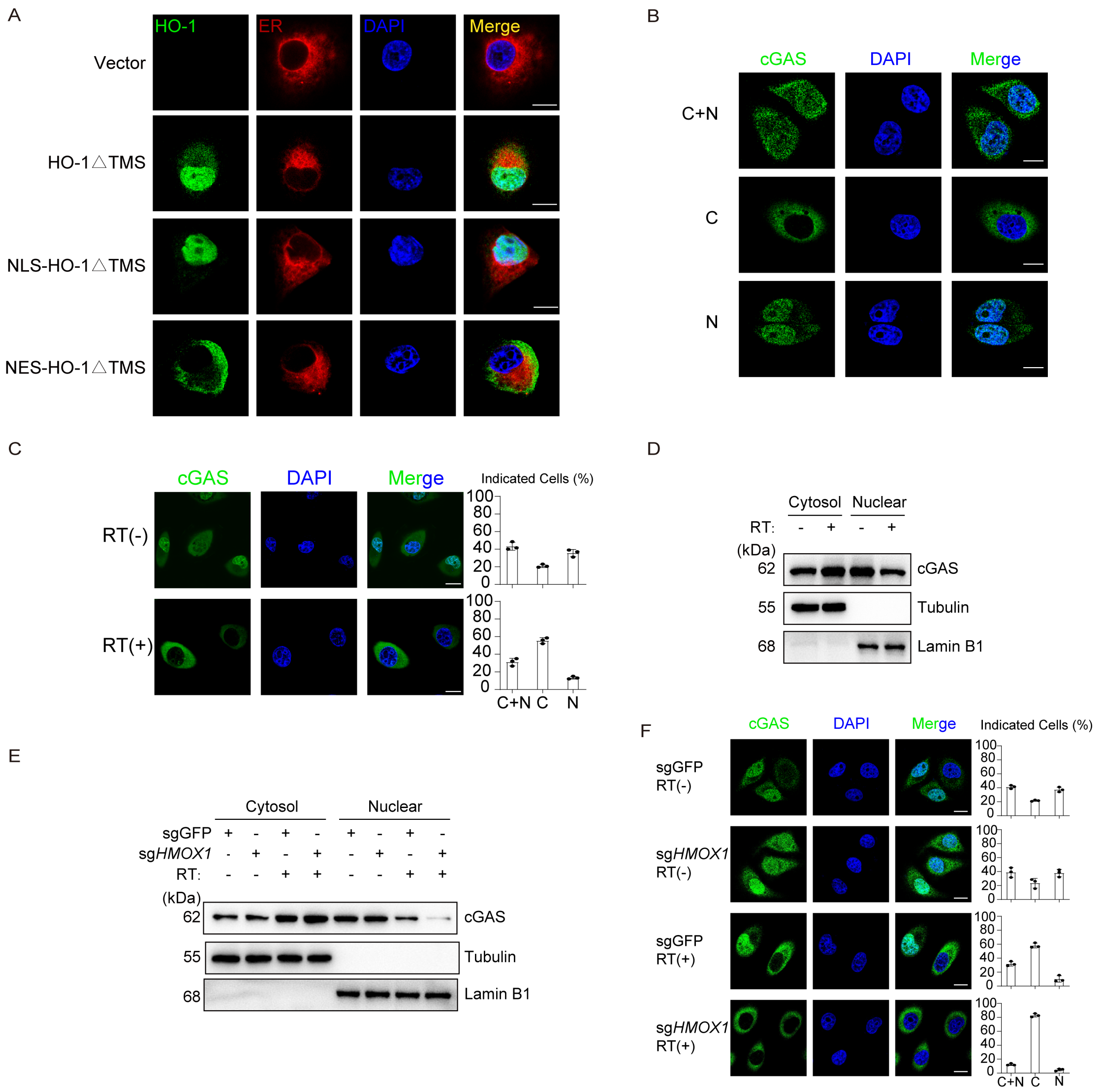

Supplemental Figure 5. Cleaved HO-1 directly interacts with cGAS and inhibits its nuclear export.

(A) Subcellular distribution of HO-1 mutants (ER and nucleus) was determined with immunofluorescence staining in HK1 cells (related to 5A-E). Calreticulin staining for ER; DAPI staining for nucleus; scale bar, 10 μm.

(B) Definition of subcellular location of cGAS: N, predominantly nuclear; C, predominately cytoplasm; C+N, evenly distributed in the nucleus and cytoplasm (scale bar, 10 μm).

(C,F) Subcellular distribution (cytoplasm and nucleus) of cGAS before and after RT was determined with immunofluorescence staining in HeLa cells (scale bar, 10 μm).

(D,E) The cytoplasmic and nuclear protein fractions were extracted for immunoblot analysis to determine the subcellular localization of cGAS before and after RT in HeLa cells.

(C,F) The percentages of N-, C-, or C+N-containing cells in 200 cells (N, predominantly nuclear; C, predominately cytoplasm; C+N, evenly distributed in the nucleus and cytoplasm) were calculated. Representative data from one experiment are shown (n = 3 biologically independent experiments). (C,F) Data are shown as the mean ± SD.

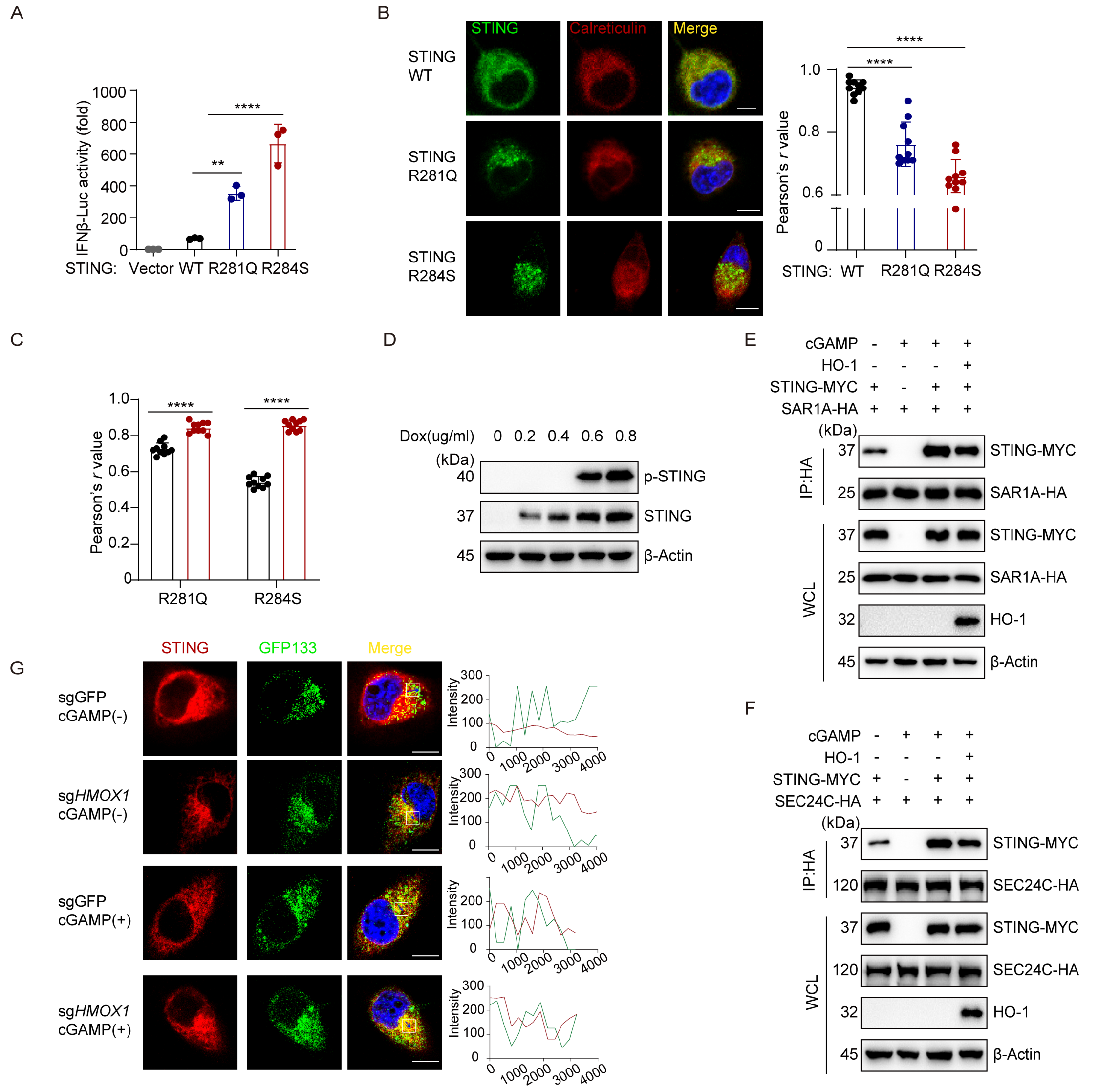

Supplemental Figure 6. HO-1 inhibits STING oligomerization and consecutive ER-to-Golgi translocation by direct interaction.

(A) Luciferase reporter assay for HEK293T cells transfected with wild-type STING or indicated STING mutants, *IFNB1* promoter luciferase reporter and Renilla.

(B) HEK293T cells were stably transfected with wild-type STING or indicated STING mutants, followed by confocal imaging. Pearson's correlation coefficient was analyzed and quantified using ImageJ.

(C) Pearson's correlation coefficient of Figure 7H was quantified using ImageJ.

(D) HK1 cells were stably transfected with doxycycline-induced STING expression plasmids. After doxycycline (DOX) treatment with indicated dose, total and phosphorylated STING determined with immunoblot analysis.

(E,F) The interaction in HEK293T cells overexpressing MYC-tagged STING and HA-tagged SAR1A (E) or SEC24C (F) was analyzed by immunoprecipitation with or without 2'3'-cGAMP treatment.

(G) 2'3'-cGAMP induced ER membrane curvature in HK1 cells. Colocalizations of STING and ER membrane curvature probed with GFP133 were determined with immunofluorescence staining.

(B,C)n = 10 cells were quantified in a blind manner. (A,B) *P*-value by One-way ANOVA. (C) *P*-value by Student's t-test. All data are shown as the mean ± SD. *P* -value < 0.05 as statistic difference. **P*<0.05; ***P*<0.01; ****P*<0.001; *****P*<0.0001. 10 μm for all scale bars.

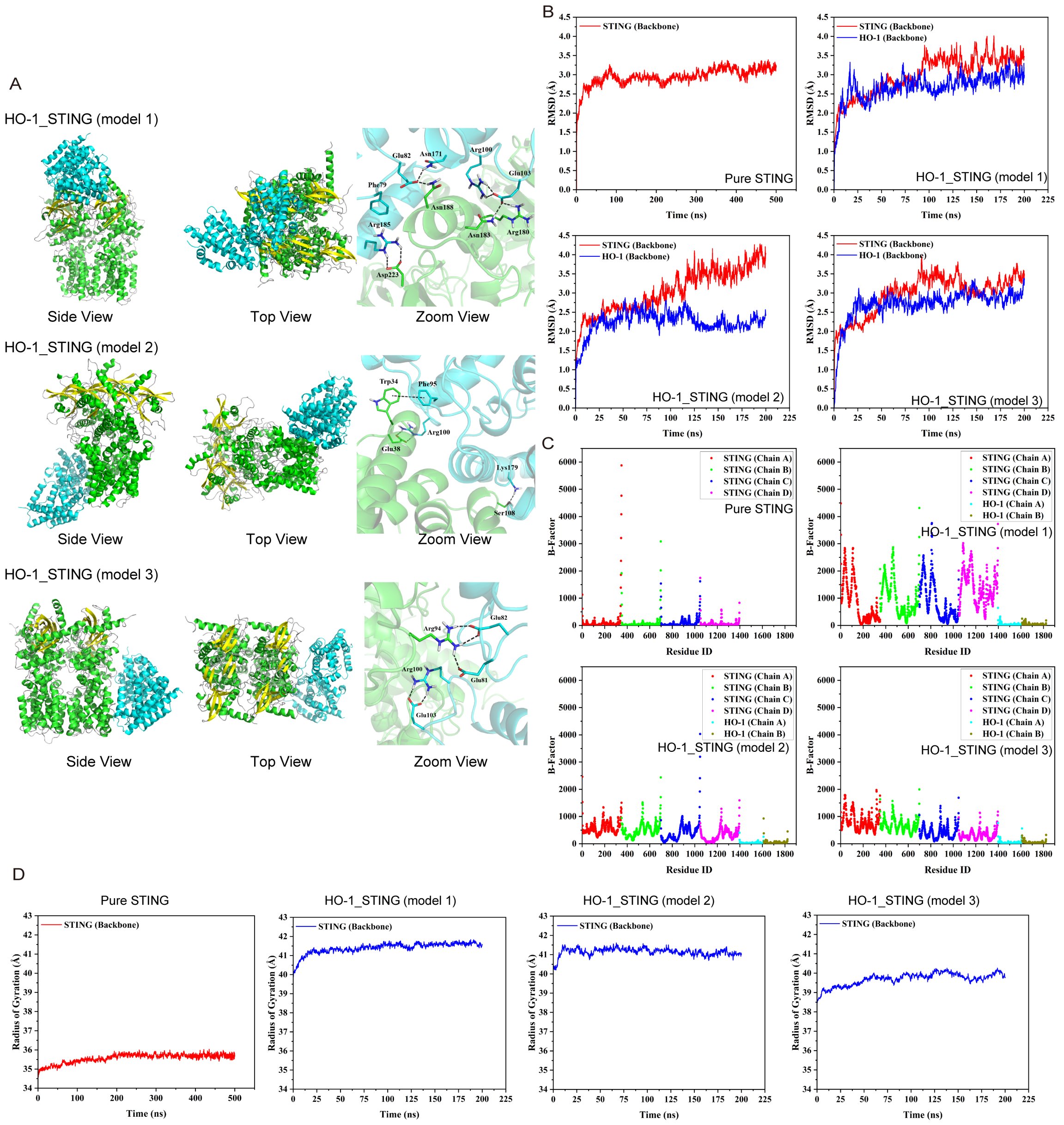

Supplemental Figure 7. Molecular docking of HO-1 and STING.

(A) The view of binding modes between the STING tetramer and HO-1 dimer based on MD simulations. (B-D) RMSD (B), RMSF (C) and Rg (D) in the pure human STING tetramer model and HO-1 dimer+STING tetramer complexes during the MD simulations.

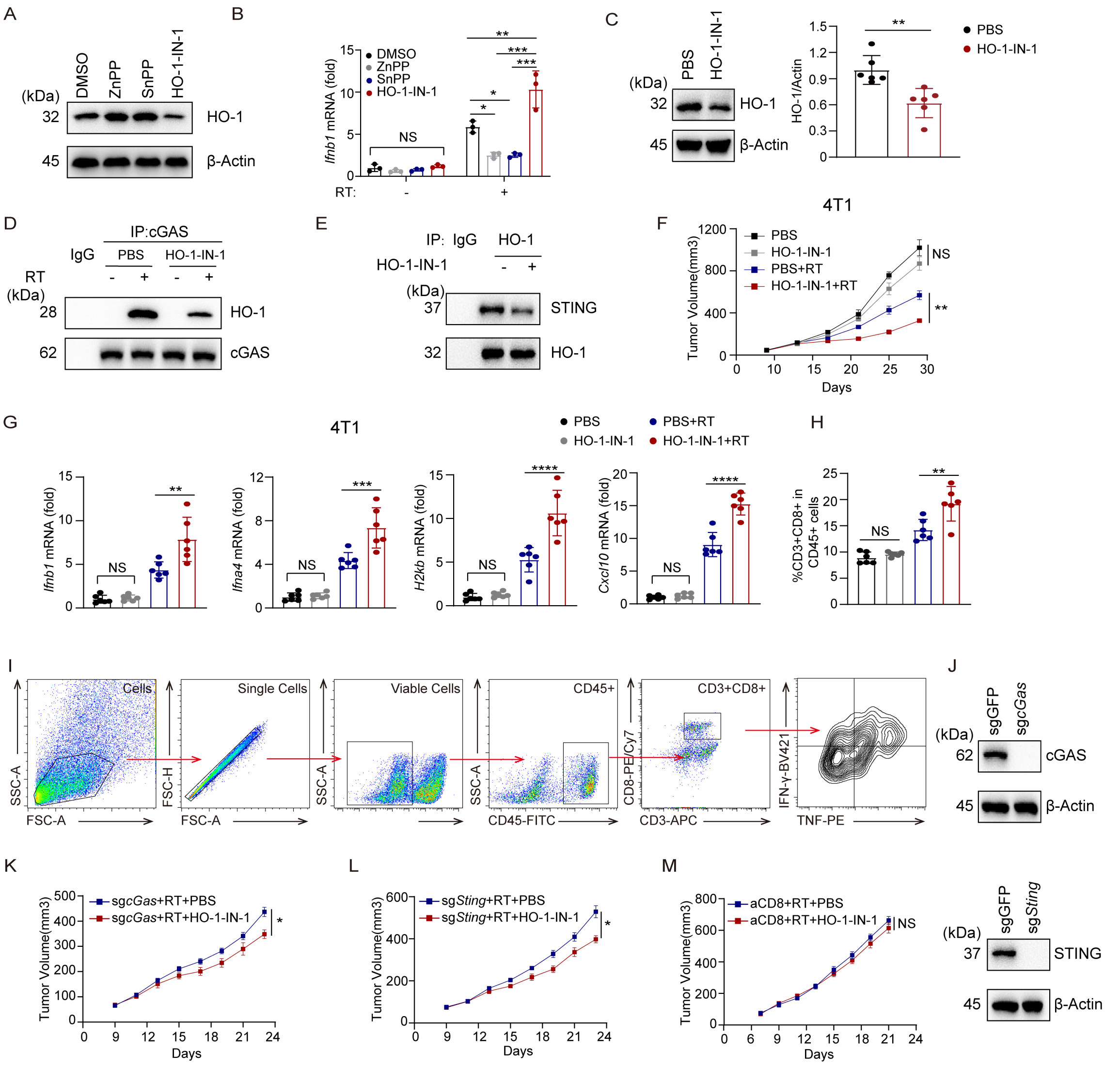

Supplemental Figure 8. HO-1 inhibitor enhances the efficacy and abscopal effect of RT in vivo.

(A) Immunoblot analysis of HO-1 expression after treating with indicated HO-1 inhibitors in MC38 cells.

(B) Quantitative PCR analysis for *Ifnb1* mRNA levels of MC38 cells treated as indicated (n=3 in each group).

(C) Immunoblot analysis of HO-1 expression in MC38-bearing mice treated with or without HO-1-IN-1 (n=6 in each group).

(D) Endogenous HO-1-cGAS interaction was analyzed by immunoprecipitation in MC38 cells treated as indicated.

(E) Endogenous HO-1-STING interaction was analyzed by immunoprecipitation in MC38 cells treated as indicated.

(F-H) The effect of HO-1 inhibitor combined with RT on tumor growth (F), mRNA levels of typical IFN-Is and ISGs (G), and CD8+ T infiltration (H) of 4T1 tumors. (n=6 in each group).

(I) Representative flow cytometry plot of gating strategy to identify IFN-γ+ and TNF-α+ in CD8+ T cells (related to Figure 9J).

(J) *cGas* and *Sting* knocking-out efficiency of MC38 cells were determined with immunoblot analysis.

(K, L) Tumor growth of *cGas*- (I) or *Sting*-deficient (J) MC38 tumors under RT. Tumor-bearing mice were treated with or without HO-1 inhibitor. (n=6 in each group).

(M) Tumor growth of anti-CD8 neutralizing antibody treated MC38 tumors combined with or without HO-1 inhibitor. (n=6 in each group).

(B,C,G,H) *P*-value by One-way ANOVA. Data are shown as the mean ± SD. (F, K,L,M) *P*-value by Two-way ANOVA. Data are shown as the mean ± SEM. All *P*-value< 0.05 as statistic difference. **P*<0.05; ***P*<0.01; ****P*<0.001; *****P*<0.0001.

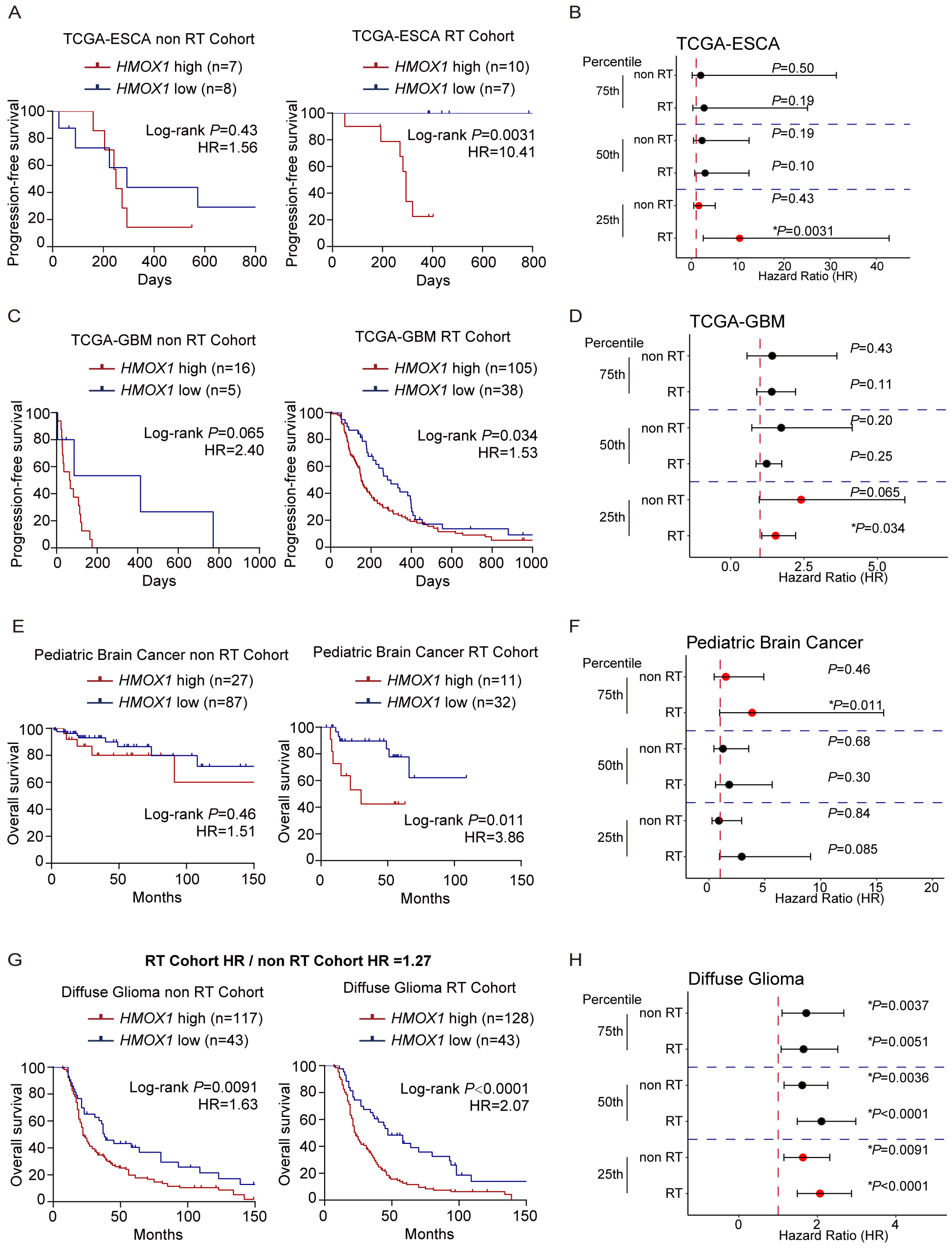

Supplemental Figure 9. High expression of HO-1 correlates with unfavorable radiotherapy prognosis. Quartiles were used as the cut-off point to distinguish between patient groups classified as "low" and "high" based on HO-1 expression. Subsequently, patients were divided into non-radiotherapy group or radiotherapy group according to the available documented information, and patients without recorded radiotherapy information were not included in the Kaplan-Meier analysis.

(A,C) Kaplan–Meier analysis of progression-free survival according to *HMOX1* expression in TCGA ESCA dataset of patients without metastasis (A) or TCGA GBM dataset (B). The lower-quartile was used as the cut-off point.

(E,G) Kaplan–Meier analysis of overall survival according to HO-1 expression in Pediatric Brain Cancer dataset (E) or Diffuse Glioma dataset (G) of patients. The upper-quartile was used as the cut-off point for (E), and the lower-quartile percentile was used as the cut-off point for (G).

(B,D,F,H) Kaplan-Meier analysis for the 25th, 50th, 75th percentiles of HO-1 expression in TCGA-ESCA dataset (B), TCGA GBM dataset (D), Pediatric Brain Cancer dataset (F) or Diffuse Glioma dataset (H). Data were presented as forest plots based on Hazard Ratio (HR) and 95% confidence interval. Red dashed line indicated HR=1.

(A-H) The *P* values were determined using the log-rank test. All *P*-value< 0.05 as statistic difference.

Supplemental Table 1. The key contact list between STING dimer-HO-1 dimer.

	STING Residue	HO-1 Residue	Interaction Type
**1**	His16	Thr222	σ-π interaction/Hydrogen Bond
**2**	Gln266	Arg100	Hydrogen Bond
**3**	Gln273	Arg100	Hydrogen Bond
**4**	Tyr274	Tyr97	Hydrogen Bond
**5**	Glu340	Arg113	Salt Bridge
**6**	Glu340	Gln212	Hydrogen Bond

Supplemental Table 2. Changes in binding affinity (ΔΔG) of STING dimer binding to HO-1 dimer upon HO-1 mutation.

	STING Residue	HO-1 Residue	HO-1 Mutation	Interaction Energy (kcal/mol)	ΔΔG (kcal/mol)
**0**			Wildtype	-14.46	0
**1**	His16	Thr222	T222A	-14.26	0.20
**2**	Gln266	Arg100	R100A	-14.07	0.39
**3**	Gln273	Arg100	R100A		
**4**	Tyr274	Tyr97	Y97A	-13.80	0.66
**5**	Glu340	Arg113	R113A	-13.27	1.19
**6**	Glu340	Gln212	Q212A	-13.45	1.01
**7**		Y97A,R100A,R113A,Q212A,T222A		-11.79	2.67

Supplemental Table 3. The binding free energy (in kcal/mol) and its components obtained from the MM/PBSA calculation for STING tetramer and STING dimer-HO-1 dimer.

Contribution	STING tetramer	STING dimer-HO-1 dimer
ΔE_{vdw}	-538.96	-547.52
ΔE_{ele}	-658.16	-700.38
ΔG_{polar}	1047.75	1044.24
$\Delta G_{nonpolar}$	-94.31	-76.57
ΔG_{total}	-243.68	-280.23

Supplemental Table 4. Correlations between HO-1 expression and the clinical

Characteristic	Low expression group N=104(100%)	High expression group N=116(100%)	*P* value*
**Age (years)**			
<45	51(49.0)	51(44.0)	0.451
⩾45	53(51.0)	65(56.0)	
**Gender**			
Male	73(70.2)	93(80.2)	0.086
Female	31(29.8)	23(19.8)	
**TNM stage**			
I+II	36(34.6)	38(32.8)	0.771
III+IV	68(65.4)	78(67.2)	
**VCA-IgA titre**			
<1:80	13(12.5)	9(7.8)	0.476
⩾1:80	83(79.8)	99(85.3)	
NA	8(7.7)	8(6.9)	
**EA-IgA titre**			
<1:10	31(29.8)	19(16.4)	0.040
⩾1:10	63(60.6)	88(75.9)	
NA	10(9.6)	9(7.8)	
**Death**			
No	92(88.5)	83(71.6)	0.002
Yes	12(11.5)	33(28.4)	
**Disease**			
No	87(83.7)	70(60.3)	0.000
Yes	17(16.3)	46(39.7)	
**Recurrence**			
No	95(91.3)	94(81.0)	0.028
Yes	9(8.7)	22(19.0)	
**Concurrent Chemotherapy**			
No	49(47.1)	44(37.9)	0.107
Yes	55(52.9)	72(62.1)	

characteristics of patients with NPC.

*Two-sided Chi-square test. All patients were restaged according to the AJCC Cancer Staging Manual, 7th edition.

Supplemental Table 5. List of reagents used in this study.

REAGENTS or RESCOURCE	SOURCE	CATALOGUE
**Chemicals**		
2′3′-cGAMP	InvivoGen	tlrl-nacga23
Digitonin	Sigma-Aldrich	11024-24-119887
IKK-IN-1	MCE	HY-13873
SCH772984	MCE	HY-50846
Fludarabine	MCE	HY-B0069
CGK733	GLPBIO	GC14526
3,4-Dichloroisocoumarin	Cayman	21194
DAPT	Selleck	S2215
$(Z\text{-}LL)_2$ Ketone	Sigma-Aldrich	313664-40-3
Heme Oxygenase-1-IN-1	MCE	1093058-52-6
Zn(II)-protoporphyrin (ZnPP)	MCE	HY-101193
Tin-protoporphyrin IX (SnPP)	MCE	HY-101194
Recombinant Human IFN-β	PeproTech	300-02BC
Doxycycline	MCE	HY-N0565B
5'ppp-dsRNA	InvivoGen	tlrl-3prna
Puromycin	TargetMol	T19978
Penicillin–streptomycin	Gibco	15140122
**Critical commercial assays**		
Human IFN-β ELISA kit	Neobioscience	EHC026
cGAMP ELISA kit	Cayman	501700
Dual luciferase assay kit	Promega	E1910
NE-PER Nuclear and Cytoplasmic Extraction Reagents	Thermofisher	78835
**Cell lines**		
HK1, human nasopharyngeal carcinoma cell line	A gift from Prof. M.S. Zeng, Sun Yat-sen University Cancer Center, Guangzhou, China	
NP69, human normal nasopharyngeal epithelial cell line		
HeLa, human cervical cancer cell line	ATCC	
DU145, human prostate cancer cell line	ATCC	
MDM-MB-231, human breast epithelial cell line	ATCC	
HT-1080, human fibrosarcoma cell line	ATCC	
HEK293T, human embryonic kidney 293T cell line	ATCC	
B16, mouse melanoma cell line	ATCC	
MC38, mouse colon adenocarcinoma cell line	ATCC	
4T1, mouse breast cancer cell line	ATCC	
MCF10A, human normal breast epithelial cell line	ATCC	
**Experimental models: Organisms/strains**		
Homx1$^{fl/fl}$ mice	Institute of Laboratory Animal Science, CAMS&PUMC	
Lyz$^{Cre/Cre}$ mice	A gift from Professor Xiaojun Xia; the Sun Yat-sen University Cancer Center (Guangzhou, China)	
huHSC-NCG	GemPharmatech Co.,Ltd.	
C57BL/6 and BALB/c	Guangdong Medical Laboratory Animal Center (Foshan, China) and GemPharmatech Co.,Ltd.	

Supplemental Table 6. Primer sequences for qPT-PCR, siRNA, shRNA, and sgRNA assays

Name	Forward	Reverse
Primer sequences for qRT-PCR		
HMOX1	5’-AAGACTGCGTTCCTGCTCAAC-3’	5’-AAAGCCCTACAGCAACTGTCG-3’
CYB5R2	5’-AGGAGGAGAGAGCCAATCACC-3’	5’-ACATAGTTACCTACAGGAAGCCC-3’
GPI	5’-CAAGGACCGCTTCAACCACTT-3’	5’-CCAGGATGGGTGTGTTTGACC-3’
KDSR	5’-GTGGCATCGGGAAGTGCAT-3’	5’-AAGCACCACCTGTTTGTCATT-3’
SLC35A5	5’-GTGCTGAAGAGGCGTCTAAAC-3’	5’-GTCCTGCCAAGTTGTGCTGT-3’
MYC	5’-GGCTCCTGGCAAAAGGTCA-3’	5’-CTGCGTAGTTGTGCTGATGT-3’
MCCC1	5’-GCTGCACAGGCTATCCATCC-3’	5’-CACCATGATAACCCTCCACAAC-3’
SCAP	5’-TATCTCGGGCCTTCTACAACC-3’	5’-GGGGCGAGTAATCCTTCACA-3’
NDUFA11	5’-GCCGAAGGTTTTTCGTCAGTA-3’	5’-GGAGGATTGAGTGTGACTCTGT-3’
KCTD21	5’-GTGACGGCAAAGTGTTCCG-3’	5’-GTTGGCGTTGAAGACCTCCAT-3’
IFNA2	5’-GCTTGGGATGAGACCCTCCTA-3’	5’-CCCACCCCCTGTATCACAC-3’
IFNB1	5’-GCTTGGATTCCTACAAAGAAGCA-3’	5’-ATAGATGGTCAATGCGGCGTC-3’
HLA-A	5’- GACGCCCCCAAAACGCATA-3’	5’- TGGGCAAACCCTCATGCTG -3’
CXCL10	5’-GTGGCATTCAAGGAGTACCTC-3’	5’-TGATGGCCTTCGATTCTGGATT-3’
Ifnb1(mouse)	5’-CAGCTCCAAGAAAGGACGAAC-3’	5’-GGCAGTGTAACTCTTCTGCAT-3’
Ifna4(mouse)	5’-TGATGAGCTACTACTGGTCAGC-3’	5’-GATCTCTTAGCACAAGGATGGC-3’
H2-kb(mouse)	5’- ACCAGCAGTACGCCTACGA -3’	5’- AACCAGAACAGCAACGGTCG-3’
Cxcl10 (mouse)	5’-CCAAGTGCTGCCGTCATTTTC-3’	5’-GGCTCGCAGGGATGATTTCAA-3’
Primer sequences for siRNA		
TRIF-1	5’-GCCAGGACAAGCUCUUGUATT-3’	5’-UACAAGAGCUUGUCCUGGCTT-3’
TRIF-2	5’-GGAUCUCUCUAGAGGCAUUTT-3’	5’-AAUGCCUCUAGAGAGAUCCTT-3’
MAVS-1	5’- CUGCCGCAAUUUCAGCAAUTT-3’	5’- AUUGCUGAAAUUGCGGCAGTT-3’

MAVS-2	5’- GCUGUGAGCUAGUUGAUCUTT-3’	5’- AGAUCAACUAGCUCACAGCTT-3’
STING-1	5’- GCCCUUCACUUGGAUGCUUTT-3’	5’- AAGCAUCCAAGUGAAGGGCTT-3’
STING-2	5’- CCGGAUUCGAACUUACAAUTT-3’	5’- AUUGUAAGUUCGAAUCCGGTT-3’
HMOX1-1	5’-CAGUUGCUGUAGGGCUUUATT-3’	5’-UAAAGCCCUACAGCAACUGTT-3’
HMOX1-2	5’-CUGGAAGACACCCUAAUGUTT-3’	5’-ACAUUAGGGUGUCUUCCAGTT-3’
CYB5R2-1	5’-GGAGAGAGCCAAUCACCUUTT-3’	5’-AAGGUGAUUGGCUCUCUCCTT-3’
CYB5R2-2	5’-GCUUUGUGGACCUAAUUAUTT-3’	5’-AUAAUUAGGUCCACAAAGCTT-3’
GPI-1	5’-CCAGGAGACCAUCACGAAUTT-3’	5’-AUUCGUGAUGGUCUCCUGGTT-3’
GPI-2	5’-GCUCACACCAUUCAUGCUUTT-3’	5’-AAGCAUGAAUGGUGUGAGCTT-3’
KDSR-1	5’-GCAUUGCUAUCGAGUGCUATT-3’	5’-UAGCACUCGAUAGCAAUGCTT-3’
KDSR-2	5’-GCAGGACAGUUGGGAUUAUTT-3’	5’-AUAAUCCCAACUGUCCUGCTT-3’
SLC35A5-1	5’-GGGCUUCCCUCCUGACUUUTT-3’	5’-AAAGUCAGGAGGGAAGCCCTT-3’
SLC35A5-2	5’-GGAAUACGCACCUAGGCAATT-3’	5’-UUGCCUAGGUGCGUAUUCCTT-3’
MYC-1	5’-GGAAGAAAUCGAUGUUGUUTT-3’	5’-AACAACAUCGAUUUCUUCCTT-3’
MYC-2	5’-GGAAACGACGAGAACAGUUTT-3’	5’-AACUGUUCUCGUCGUUUCCTT-3’
MCCC-1	5’-GGAAUGAGGAUUGUUAGAUTT-3’	5’-AUCUAACAAUCCUCAUUCCTT-3’
MCCC-2	5’-CACCAACAUUGACUUCUUATT-3’	5’-UAAGAAGUCAAUGUUGGUGTT-3’
SCAP-1	5’-CCUACCUUGUGGUGGUUAUTT-3’	5’-AUAACCACCACAAGGUAGGTT-3’
SCAP-2	5’-CGACGCUCUUCAGCUAUUATT-3’	5’-UAAUAGCUGAAGAGCGUCGTT-3’
NDUFA11-1	5’-CCACCAGUAUUGCCAGCGUTT-3’	5’-ACGCUGGCAAUACUGGUGGTT-3’
NDUFA11-2	5’-GCCUGCGUGUACUUUGGCATT-3’	5’-UGCCAAAGUACACGCAGGCTT-3’
KCTD21-1	5’-GGGAAGCUCUAUACAACCUTT-3’	5’-AGGUUGUAUAGAGCUUCCCTT-3’
KCTD21-2	5’-GCAAAGUGUUCCGCUAUAUTT-3’	5’-AUAUAGCGGAACACUUUGCTT-3’
Primer sequences for shRNA		
Hmox1-1(mouse)	5’-CCGG-AGCCACACAGCACTATGTAAA-CTCGAG-TTTACATAGTGCTGTGTGGCT-TTTTTT-3’	

Hmox1-2(mouse)	5’- CCGG-ACAGTGGCAGTGGGAATTTAT-CTCGAG-ATAAATTCCCACTGCCACTGT-TTTTTT -3’	
Primer sequences for sgRNA		
HMOX1-1	5’-CACCGAGGGCCTCTGACAAATCCTG-3’	5’-AAACCAGGATTTGTCAGAGGCCCTC-3’
HMOX1-2	5’-CACCGAAGGGCCAGGTGACCCGAGA-3’	5’-AAACTCTCGGGTCACCTGGCCCTTC-3’
cGAS-1	5’-CACCGCCGCGATGATATCTCCACGG-3’	5’-AAACCCGTGGAGATATCATCGCGGC-3’
cGAS-2	5’-CACCGGCTTCCGCACGGAATGCCAG-3’	5’-AAACCTGGCATTCCGTGCGGAAGCC-3’
STING-1	5’-CACCGGCTGGGACTGCTGTTAAACG-3’	5’-AAACCGTTTAACAGCAGTCCCAGCC-3’
STING-2	5’-CACCGCCATCCATCCCGTGTCCCAG-3’	5’-AAACCTGGGACACGGGATGGATGGC-3’
Sting-1(mouse)	5’-CACCGCCAGCCATCCCACGGCCCAG-3’	5’-AAACCTGGGCCGTGGGATGGCTGGC-3’
Sting-2(mouse)	5’-CACCGTGTAGCCCTCATCTTTCTGG-3’	5’-AAACCCAGAAAGATGAGGGCTACAC-3’
cGas-1(mouse)	5’-CACCGGAAACGCAAAGATATCTCGG -3’	5’-AAACCCGAGATATCTTTGCGTTTCC -3’
cGas-2(mouse)	5’- CACCGCGAGACGGTGAATAAAGTTG-3’	5’-AAACCAACTTTATTCACCGTCTCGC -3’

Supplemental Table 7. List of antibodies used in this study.

**Antibodies used for Western blotting (WB) and immunoprecipitation (IP).**							
Primary antibodies	Supplier	Catalogue	Application	Host species	Species activity	clone	Dilution
anti-cGAS	Cell Signaling Technology	79978	WB	Rabbit	Hu	E5V3W	1:1000
anti-cGAS	Cell Signaling Technology	31659	WB, IP	Rabbit	Mo	D3O8O	1:1000 for WB, 1:200 for IP
anti-cGAS	Cell Signaling Technology	83623	IP	Rabbit	Hu	E9G9G	1:100
anti-STING	Cell Signaling Technology	13647	WB, IP	Rabbit	Hu, Mo	D2P2F	1:2000 for WB, 1:50 for IP
anti-pSTING	Cell Signaling Technology	50907	WB	Rabbit	Hu	E9A9K	1:1000
anti-pSTING	Cell Signaling Technology	72971	WB	Rabbit	Mo	D8F4W	1:1000
anti-TBK1	Cell Signaling Technology	38066	WB	Rabbit	Hu	E8I3G	1:1000
anti-pTBK1	Cell Signaling Technology	5483	WB	Rabbit	Hu	D52C2	1:1000
anti-IRF3	Cell Signaling Technology	4302	WB	Rabbit	Hu	D83B9	1:1000
anti-pIRF3	Cell Signaling Technology	29047	WB	Rabbit	Hu	D6O1M	1:1000
anti-STAT1	Cell Signaling Technology	14994	WB	Rabbit	Hu	D1K9Y	1:1000
anti-pSTAT1	Cell Signaling Technology	9167	WB	Rabbit	Hu	58D6	1:1000
anti-HO-1	Cell Signaling Technology	43966	WB, IP	Rabbit	Hu, Mo	E3F4S	1:1000 for WB, 1:100 for IP
anti-HO-1	Cell Signaling Technology	82551	WB	Rabbit	Hu, Mo	E7U4W	1:1000 for WB
anti-HO-1	Cell Signaling Technology	26416	WB	Rabbit	Hu	E8B7A	1:1000 for WB
anti-CRM1	Cell Signaling Technology	46249	WB	Rabbit	Hu	D6V7N	1:1000 for WB
anti-FLAG	Cell Signaling Technology	14793	WB	Rabbit	Hu	N. A	1:2000
anti-HA	Cell Signaling Technology	3724	WB	Rabbit	Hu	N. A	1:2000
anti-Myc	Cell Signaling Technology	2278	WB	Rabbit	Hu	N. A	1:2000

anti-Beta Actin	Proteintech	66009	WB	Mouse	Hu, Mo	2D4H5	1:20000
anti-Alpha Tublin	Proteintech	66031	WB	Mouse	Hu, Mo	1E4C11	1:20000
anti-Lamin B1	Proteintech	12987	WB	Rabbit	Hu	N. A	1:10000
anti-Phospho-Histone H2A.X (Ser139)	Cell Signaling Technology	2577	WB	Rabbit	Hu	N. A	1:1000
IgG control Polyclonal antibody	Proteintech	30000-0-AP	IP	Rabbit	Hu	N. A	5μg for IP
PierceTM Protein A/G Magnetic Beads	ThermoFisher	88802	IP	N. A	Hu	N. A	25ul per test for IP
Pierce™ Anti-DYKDDDDK Magnetic Agarose	ThermoFisher	A36797	IP	N. A	Hu	N. A	25ul per test for IP
Pierce™ Anti-HA Magnetic Beads	ThermoFisher	88836	IP	N. A	Hu	N. A	25ul per test for IP
Pierce™ Anti-c-Myc Magnetic Beads	ThermoFisher	88842	IP	N. A	Hu	N. A	25ul per test for IP
anti-mouse IgG, HRP-linked Antibody	Cell Signaling Technology	7076	WB	Horse	Mo	N. A	1:5000
anti-rabbit IgG, HRP-linked Antibody	Cell Signaling Technology	7074	WB	Goat	Rabbit	N. A	1:5000
**Antibodies used for flow cytometric analysis.**							
Primary antibodies	Supplier	Catalogue	Application	Host species	Species activity	clone	Dilution
FITC anti-mouse CD45	Biolegend	103107	Fc	Rat	Mo	30-F11	5ug per test

APC anti- mouse CD3ε	Biolegend	100311	Fc	Rat	Mo	145-2C11	5ug per test
PC7 anti-mouse CD8α	Biolegend	100721	Fc	Rat	Mo	53-6.7	5ug per test
PE anti-mouse TNFα	Biolegend	506306	Fc	Rat	Mo	MP6-XT22	5ug per test
BV421 anti-mouse IFNγ	Biolegend	505829	Fc	Rat	Mo	XMG1.2	5ug per test
FITC anti-human CD45	Biolegend	304006	Fc	Mo	Hu	HI30	5ug per test
PE anti-human CD3	Biolegend	317308	Fc	Mo	Hu	OKT3	5ug per test
BV605 anti-human CD8	Biolegend	344741	Fc	Mo	Hu	SK1	5ug per test
APC anti-human IFN-γ	Biolegend	502512	Fc	Mo	Hu	4S.B3	5ug per test
PE/Cyanine7 anti-human TNF-α	Biolegend	502929	Fc	Mo	Hu	MAb11	5ug per test
Zombie NIR™ Fixable Viability Kit	Biolegend	423105	Fc	N. A	N. A	N. A	1:1000
Zombie UV™ Fixable Viability Kit	Biolegend	423107	Fc	N. A	N. A	N. A	1:1000
**Antibodies used for immunofluorescence (IF).**							
Primary antibodies	Supplier	Catalogue	Application	Host species	Species activity	clone	Dilution
anti-HO-1	Cell Signaling Technology	82551	IF	Rabbit	Hu	E7U4W	1:3000
anti-HO-1	Proteintech	66743	IF	Mouse	Hu	2D10A5	1:200

anti-cGAS	Cell Signaling Technology	79978	IF	Rabbit	Hu	E5V3W	1:200
anti-STING	Proteintech	19851	IF	Rabbit	Hu	N. A	1:100
488 conjugated anti-STING	Proteintech	CL488-19851	IF	Rabbit	Hu	N. A	1:100
anti-Calreticulin	Cell Signaling Technology	12238	IF	Rabbit	Hu	D3E6	1:400
anti-GM130	Cell Signaling Technology	12480	IF	Rabbit	Hu	D6B1	1: 3000
anti-FLAG	Cell Signaling Technology	14793	IF	Rabbit	Hu	N. A	1:1000
**Antibodies used for Neutralizing antibody.**							
Primary antibodies	Supplier	Catalogue	Application	Host species	Species activity	clone	Dilution
anti-mouse CD8α	Bioxcell	BE0061	Neutralizing	N. A	Mo	2.43	200μg per test
