# Supplementary material for: HO-1 impairs the efficacy of radiotherapy by redistributing cGAS and STING in tumors
Source: J Clin Invest. 2024 Dec 2;134(23):e181044. doi: 10.1172/JCI181044 (PMC11601901; doi:10.1172/JCI181044)

Figure 3A

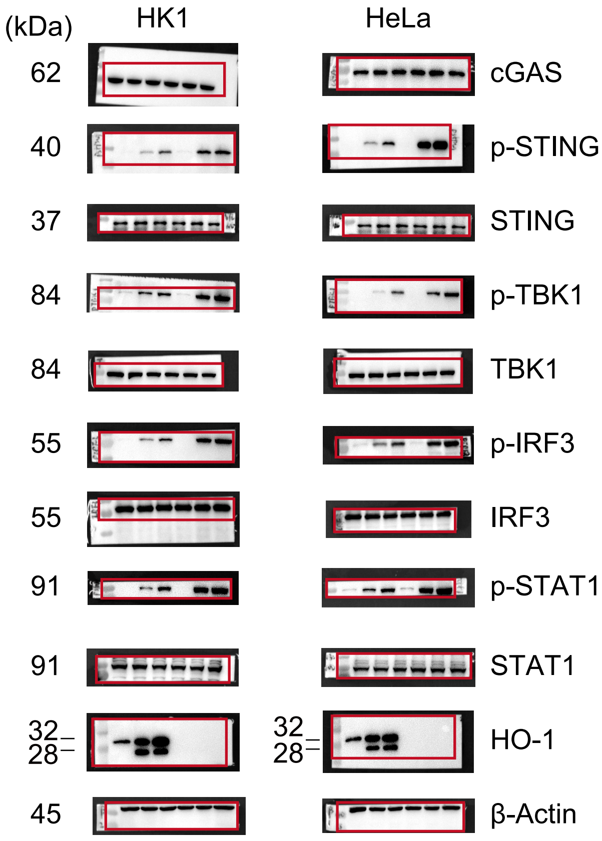

Figure 3D

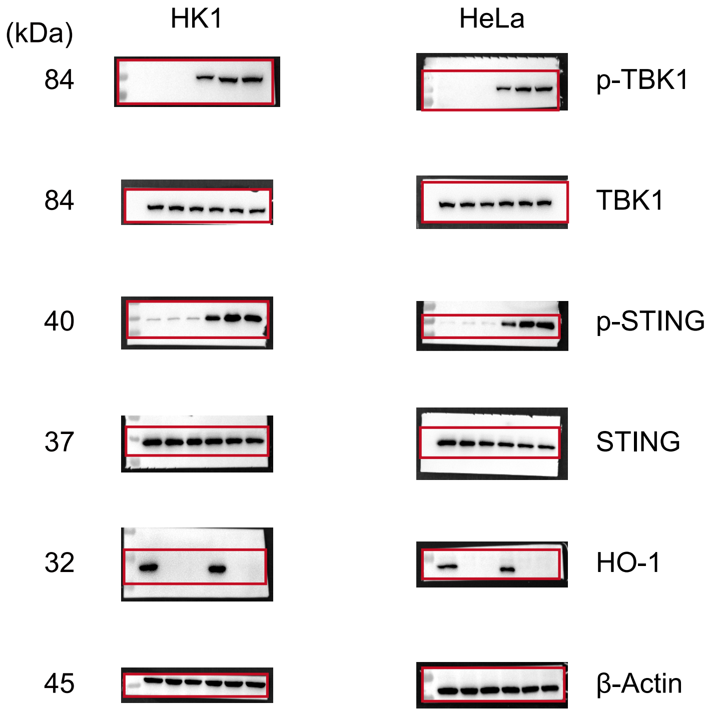

Figure 3E

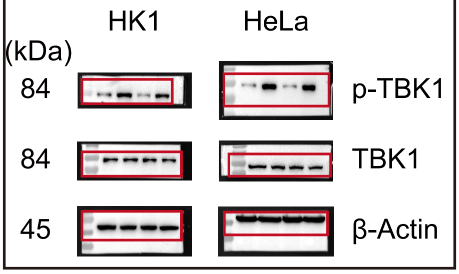

Supplemental Figure 2A

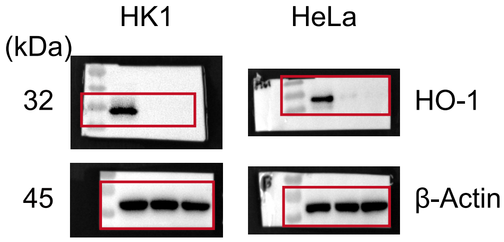

Supplemental Figure 2C

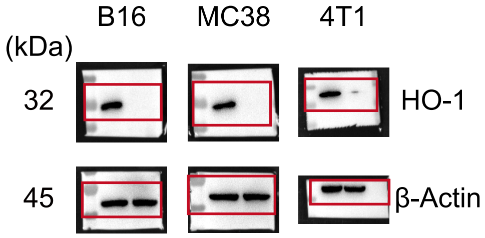

Supplemental Figure 2F

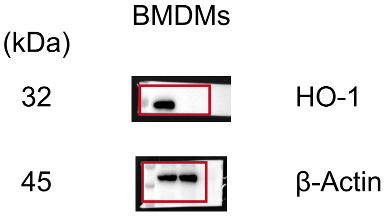

Supplemental Figure 3F

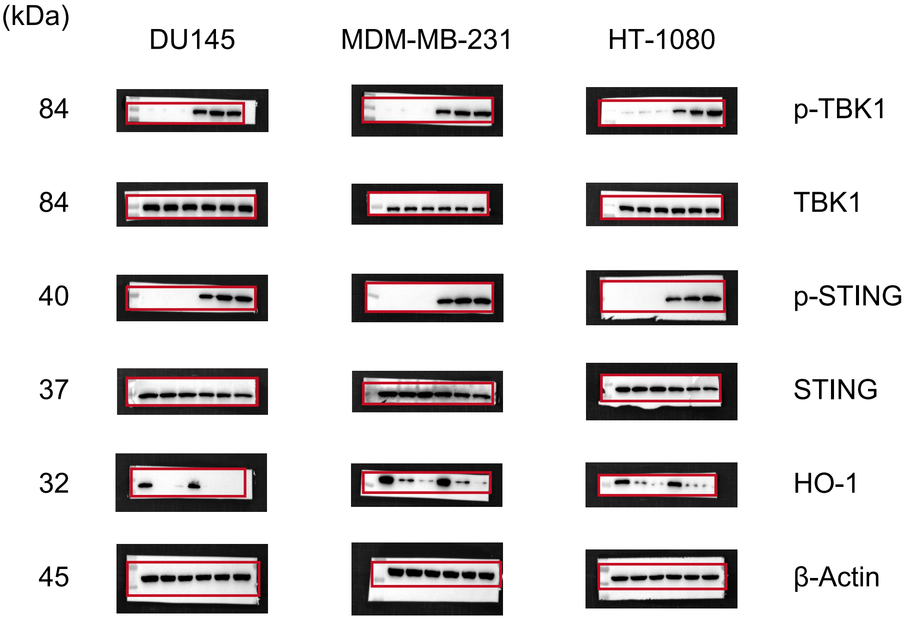

Supplemental Figure 3G

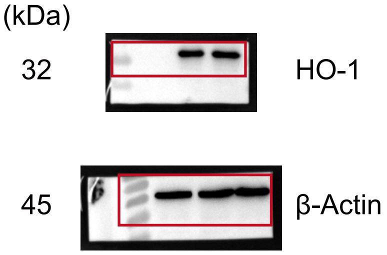

Figure 4A

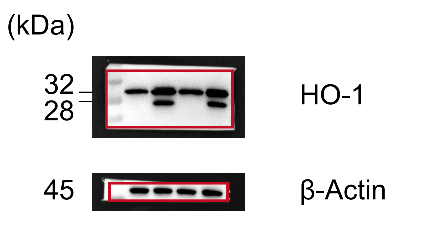

Figure 4B

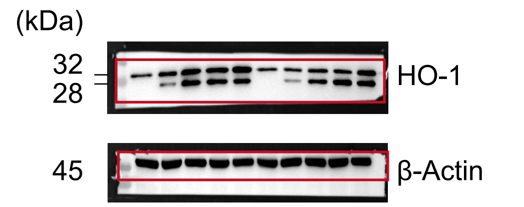

Figure 4C

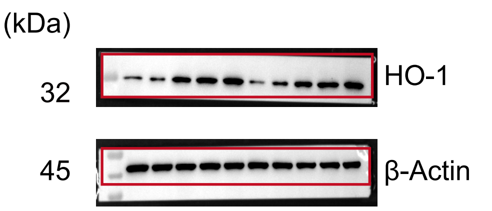

Figure 4D

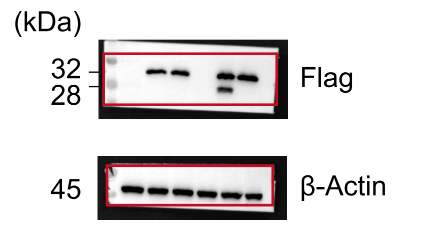

Figure 4E

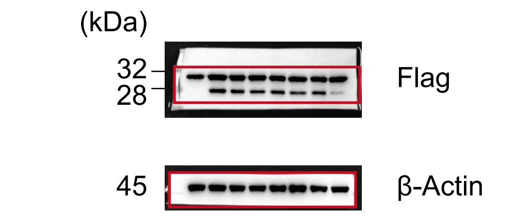

Figure 4G

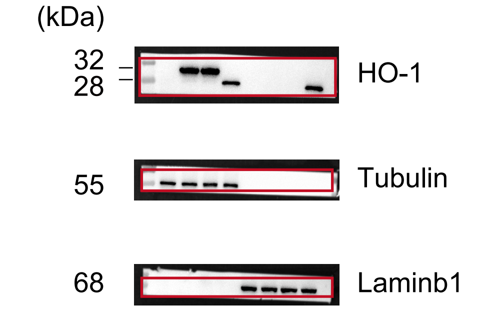

Figure 4H

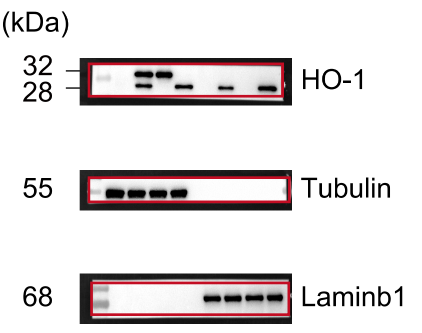

Figure 4J

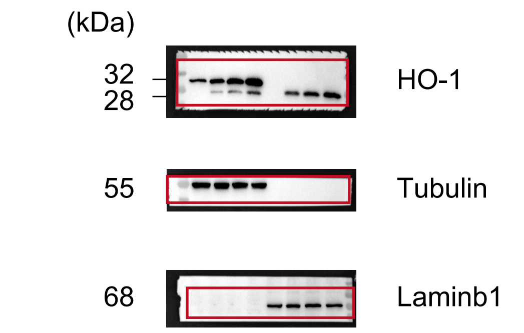

Figure 4M

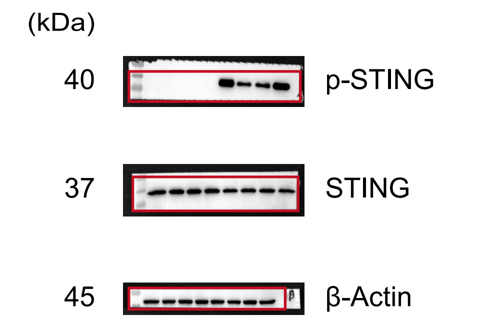

Supplemental Figure 4A

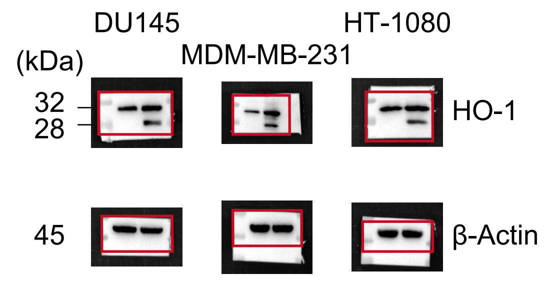

Supplemental Figure 4B

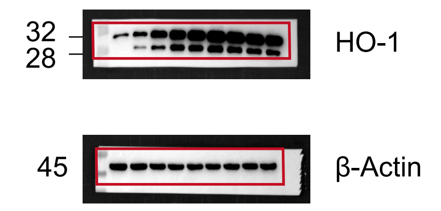

Supplemental Figure 4C

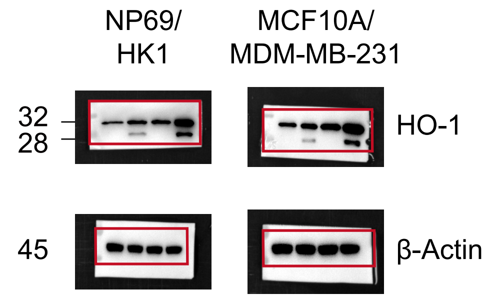

Supplemental Figure 4D

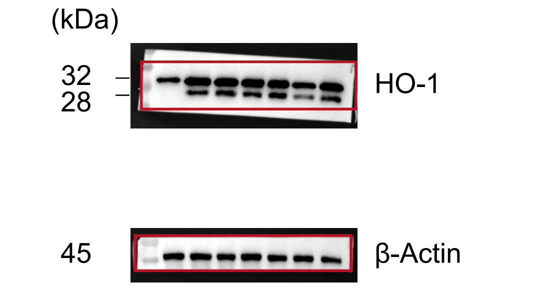

Supplemental Figure 4G

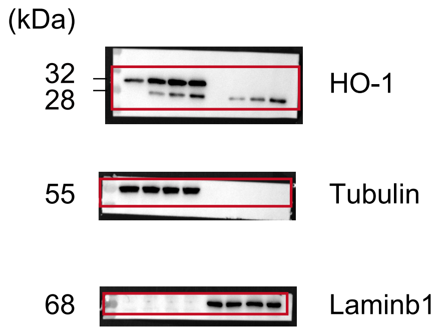

Supplemental Figure 4H

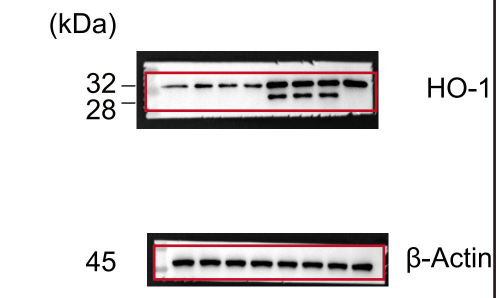

Figure 5C

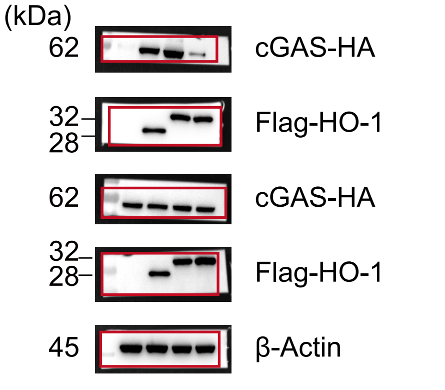

Figure 5E

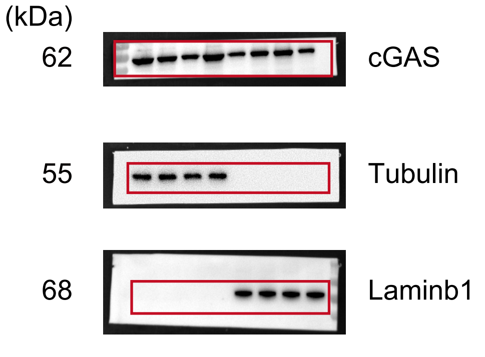

Figure 5G

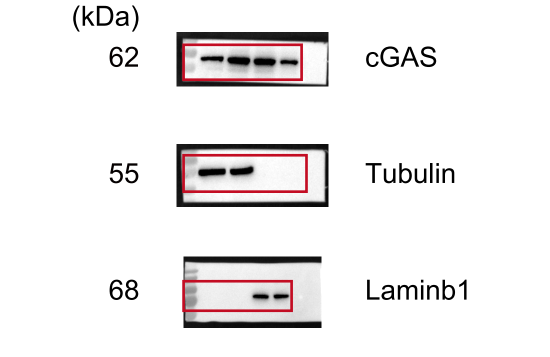

Figure 6A

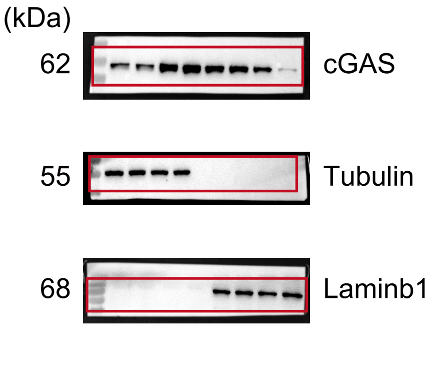

Supplemental Figure 5E

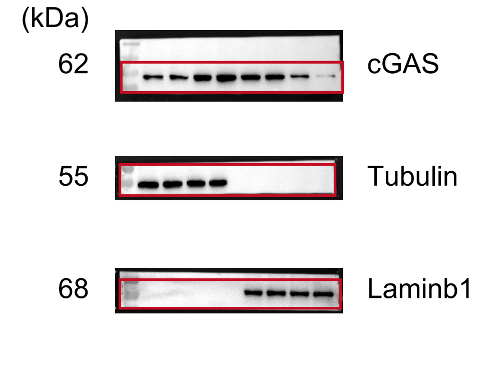

Figure 6C

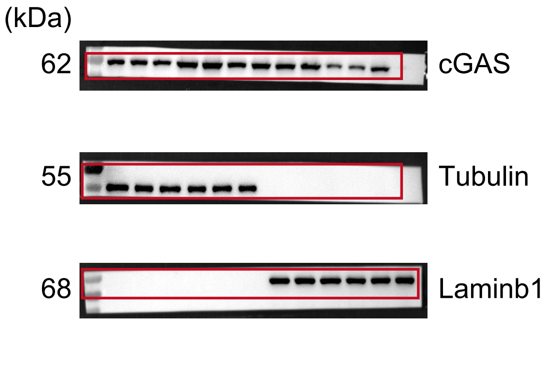

Figure 6F

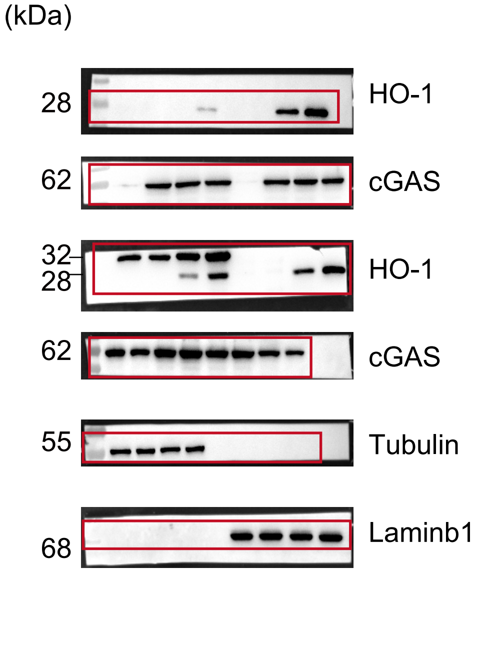

Figure 6H

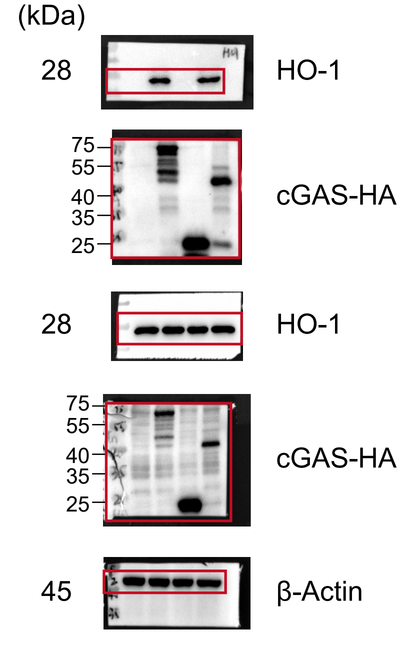

Figure 6I

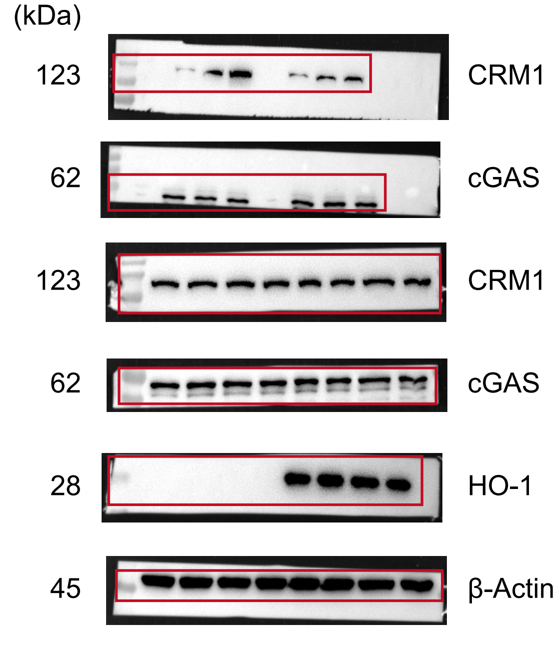

Supplemental Figure 5D

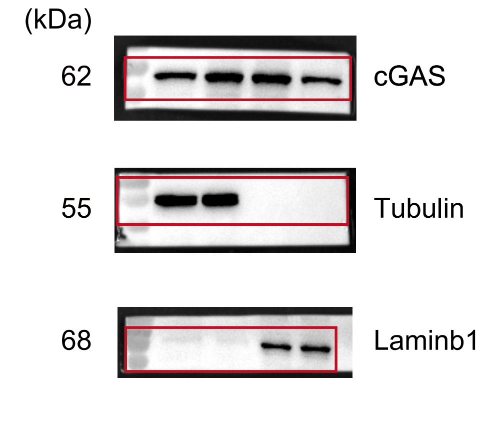

Figure 7B

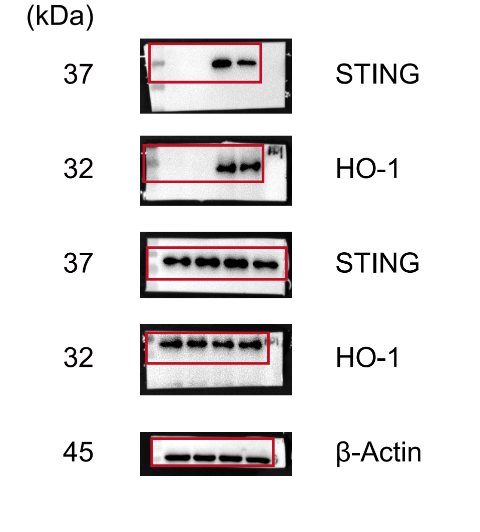

Figure 7E

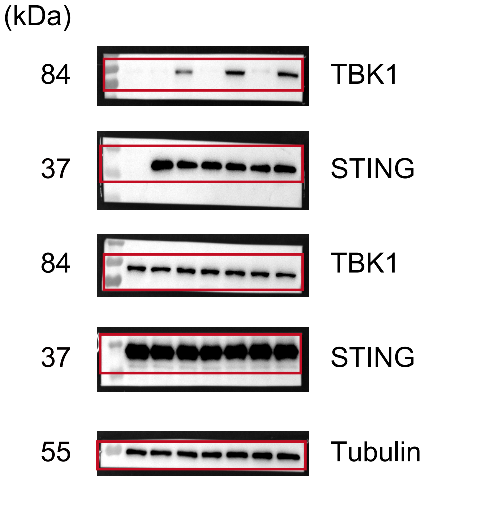

Figure 7F

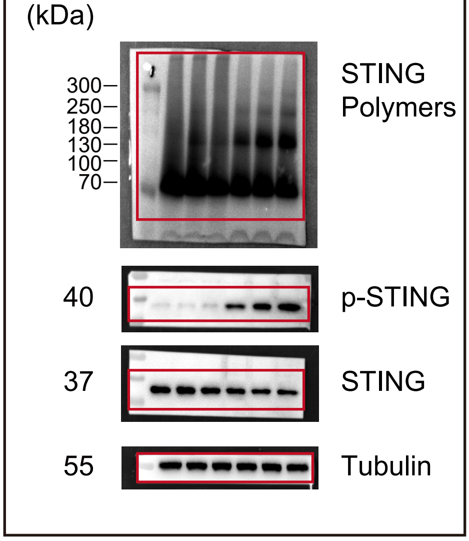

Figure 7G

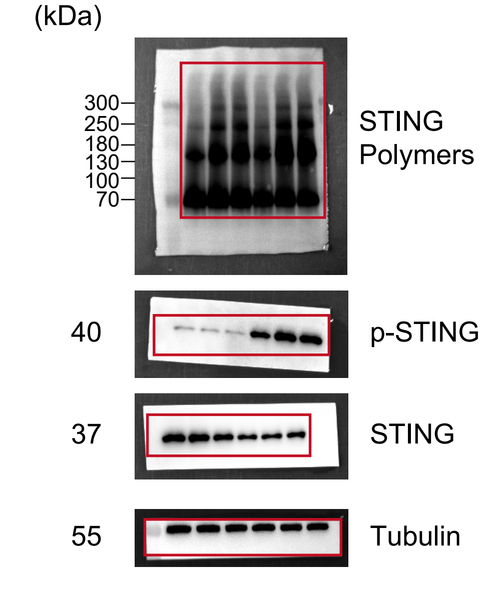

Figure 7I

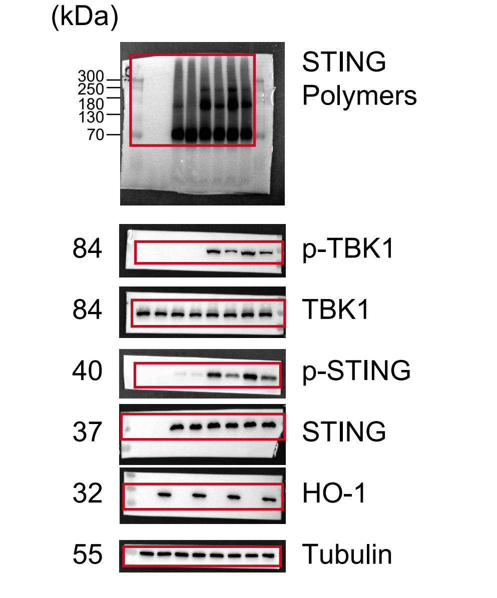

Figure 7J

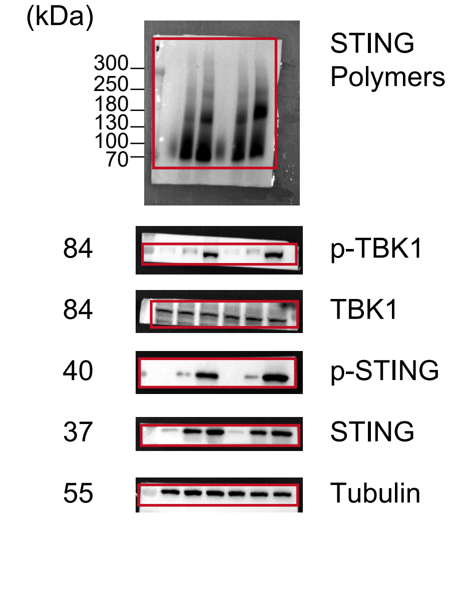

Figure 8B

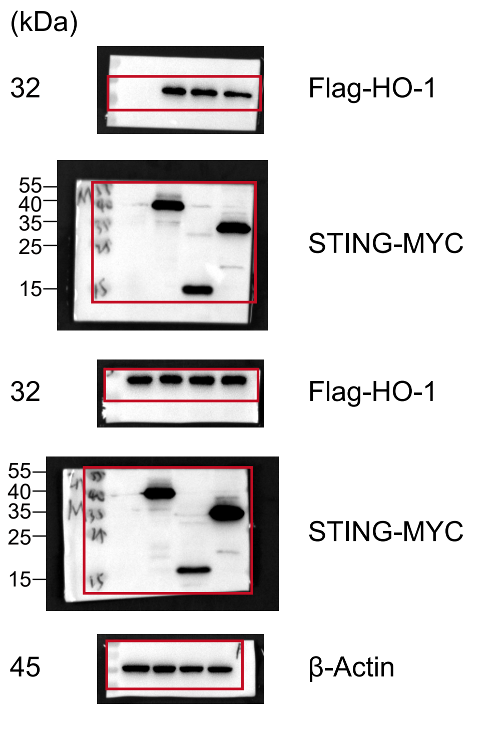

Figure 8D

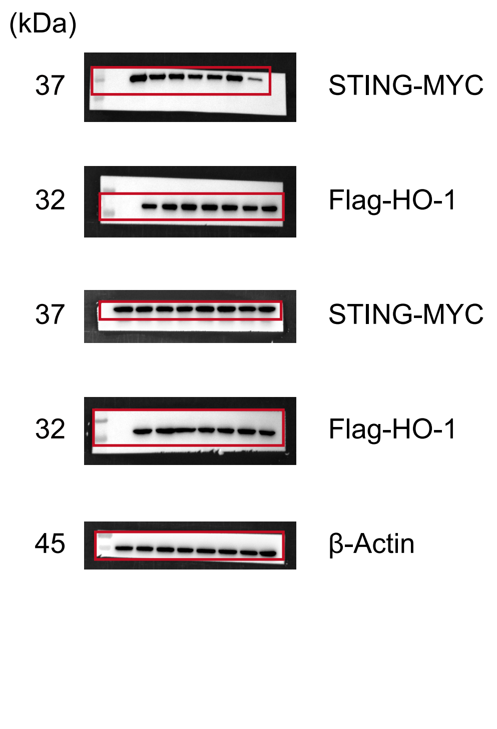

Figure 8E

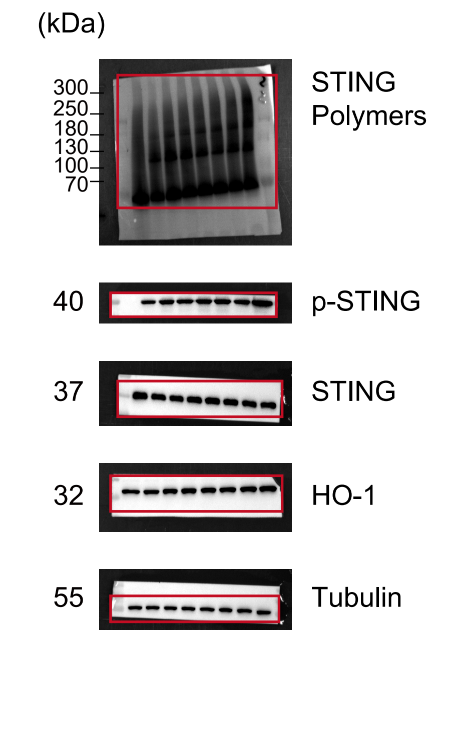

Figure 9A

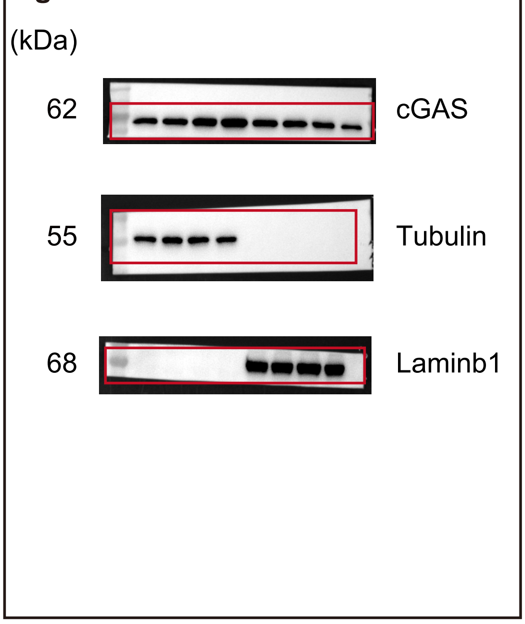

Figure 9C

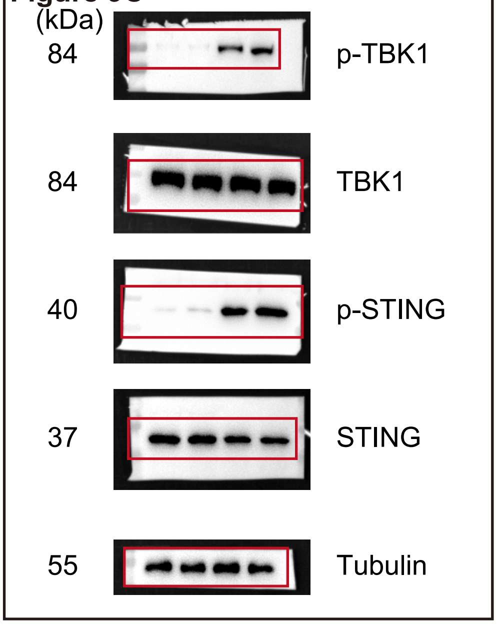

Supplemental Figure 8A

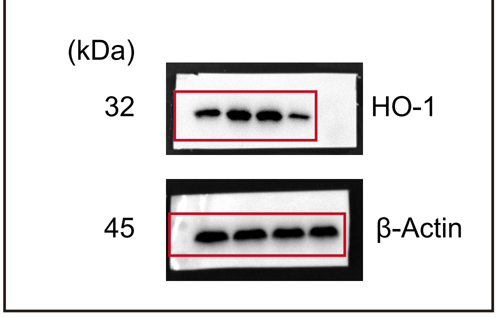

Supplemental Figure 8C

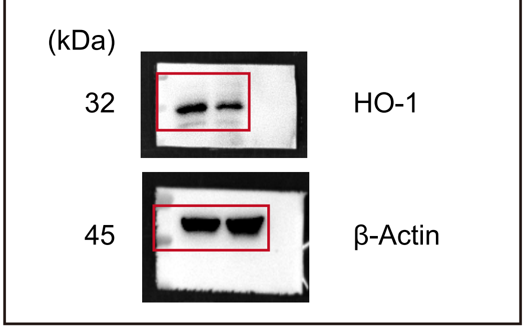

Supplemental Figure 8D

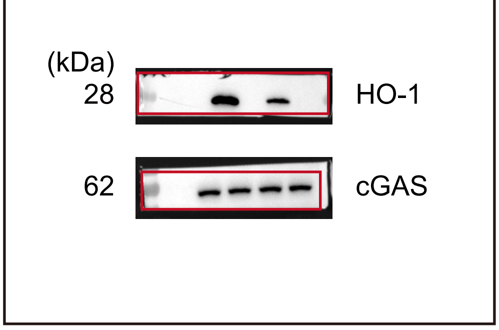

Supplemental Figure 8E

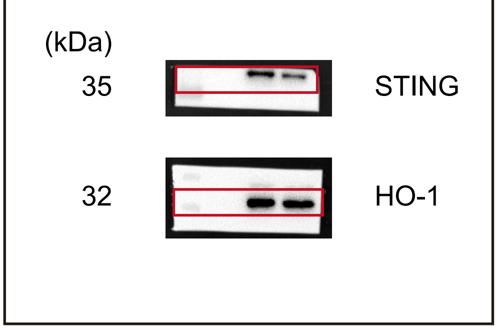

Supplemental Figure 8J

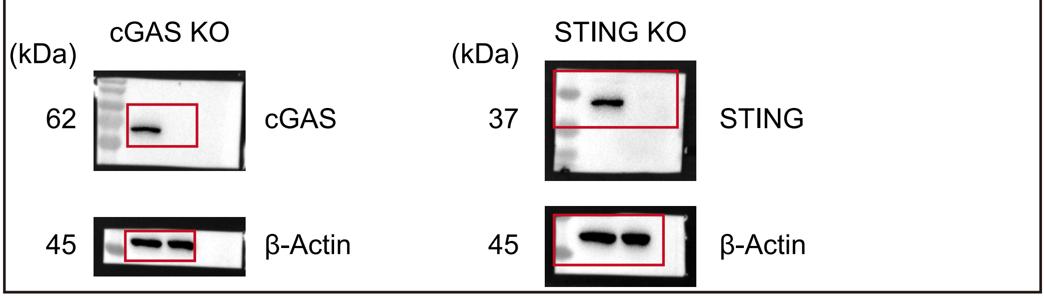

Supplemental Figure 6D

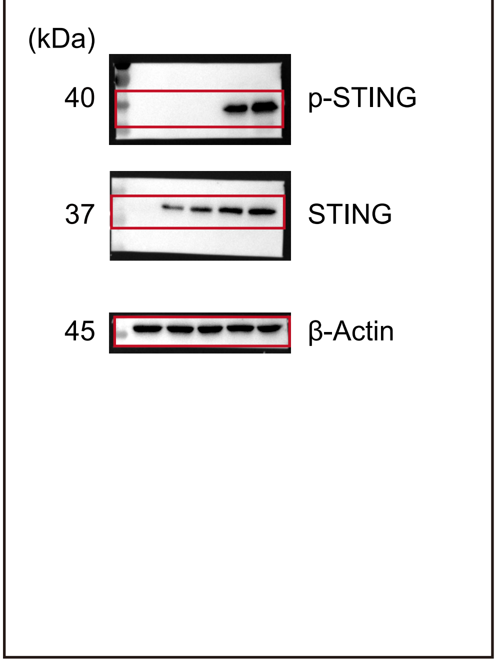

Supplemental Figure 6E

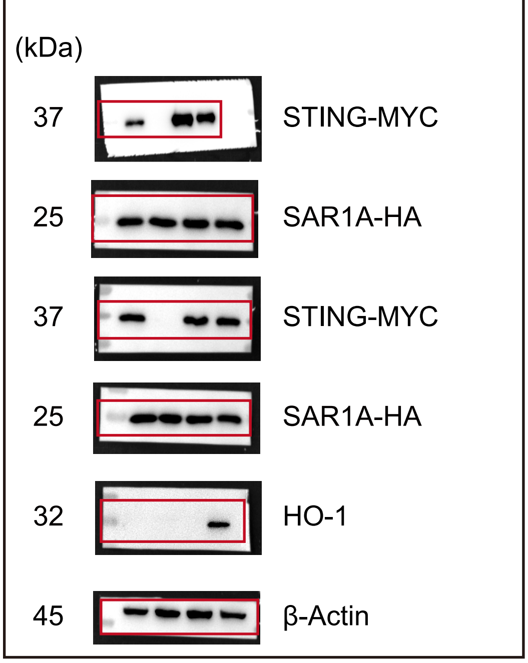

Supplemental Figure 6F

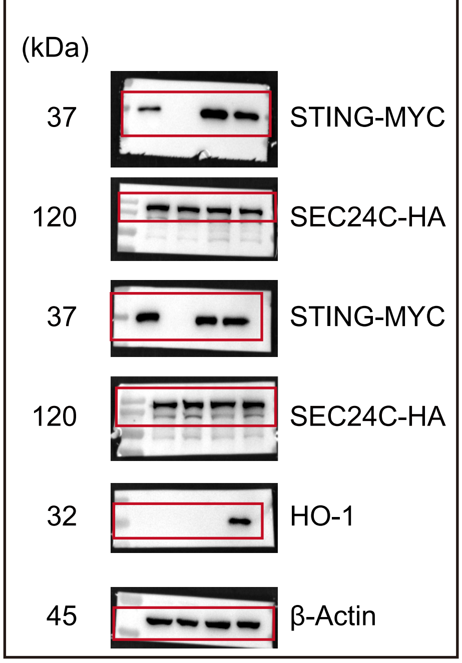

Supplement: Unedited blot and gel images [file jci-134-181044-s085.pdf]
